# Supplementary material for: Identification and Tracking of Alloreactive T Cell Clones in Rhesus Macaques Through the RM-scTCR-Seq Platform
Source: Front Immunol. 2022 Jan 26;12:804932. doi: 10.3389/fimmu.2021.804932 (PMC8825351; doi:10.3389/fimmu.2021.804932)
Supplement: Supplementary file 1 [file DataSheet_1.pdf]

.

Supplementary Table S1: V2 primer

VDJ Library from NHP 279 MLR Sample	
	V2 Primers
Estimated Number of Cells	3,210
Mean Read Pairs per Cell	21,115
Number of Cells With Productive V-J Spanning Pair	1,818
Number of Read Pairs	67,779,150
Valid Barcodes	91.0%
Q30 Bases in Barcode	95.4%
Q30 Bases in RNA Read 1	94.6%
Q30 Bases in UMI	94.8%
Reads Mapped to Any V(D)J Gene	47.1%
Reads Mapped to TRA	27.6%
Reads Mapped to TRB	19.4%
Mean Used Read Pairs per Cell	4098
Fraction Reads in Cells	47.3%
Median TRA UMIs per Cell	58
Median TRB UMIs per Cell	122
Cells With Productive V-J Spanning Pair	56.6%
Cells With Productive V-J Spanning (TRA, TRB) Pair	56.6%
Paired Clonotype Diversity	126.48
Cells With TRA Contig	95.6%
Cells With TRB Contig	87.8%
Cells With CDR3-annotated TRA Contig	93.1%
Cells With CDR3-annotated TRB Contig	84.7%
Cells With V-J Spanning TRA Contig	93.8%
Cells With V-J Spanning TRB Contig	85.0%
Cells With Productive TRA Contig	83.1%
Cells With Productive TRB Contig	73.6%

Supplementary Table 2: Cell counts and VDJ statistics

Sample ID			Cell Loading				GEX (Gene Expression) Cell Counts				
NHP_Sample_ID	Sample_Type	NHP_Donor_ID	Cells_Loaded	Expected_Recovery_If_All_Viable	Percent Viable	Expected_Cells	GEX_Cell_Count_Before_Filtering	GEX_Cell_Count_After_Filtering	Number of Read Pairs	Valid Barcodes	Q30 Bases in Barcode
R.227	Liver	R.276	14000	10000	14.9	1490	1005	441	119419557	0.964	0.959
R.227	Spleen	R.276	11000	9000	16.7	1503	1642	1037	116994014	0.967	0.957
R.276	Liver	R.279	160000	10000	78.4	7840	5285	4083	111023012	0.956	0.959
R.276	MLR	R.276	2610	2000	77.3	1546	676	387	126689249	0.964	0.958
R.276	Spleen	R.279	81000	10000	82.5	8250	3479	2810	120533868	0.966	0.959
R.276	Blood	R.276	2770	1000	30.4	304	621	424	112559636	0.968	0.96
R.279	CD3	R.279	11727	10000	72.4	7240	4717	NA	107091416	0.955	0.958
R.279	MLR	R.279	4932	4000	77.1	3084	889	823	92084798	0.954	0.958
R.279	Blood	R.279	1000000	10000	74.3	7430	1412	1256	89200161	0.95	0.959
R.312	Spleen	R.319	22500	10000	51.9	5190	5801	4671	109437654	0.961	0.959
R.319	CD3	R.319	2770	2000	67.2	1344	987	NA	100946106	0.969	0.96
R.319	MLR	R.319	14781	10000	83.7	8370	4218	2833	104368559	0.956	0.959
R.319	Blood	R.319	42000	10000	50.5	5050	7415	4853	86788851	0.962	0.959
R.312	Liver	R.319	40000	10000	58.5	5850	NA	NA	33675819	0.977	0.956
R.329	High	R.329	918000	10000	86.3	8630	NA	NA	58017375	0.968	0.945
R.329	Low	R.329	543000	10000	92.9	9290	NA	NA	51027913	0.959	0.945
R.329	Mid	R.329	69900	10000	95.3	9530	NA	NA	52076066	0.965	0.945
R.329	Blood	R.329	339000	10000	99	9900	NA	NA	52411977	0.967	0.945
R.330	High	R.330	946000	10000	89.5	8950	NA	NA	52729939	0.968	0.945
R.330	Low	R.330	189000	10000	90.1	9010	NA	NA	51875058	0.959	0.945
R.330	Mid	R.330	6910	5000	66.6	3330	NA	NA	51914507	0.953	0.945
R.330	Blood	R.330	210000	10000	95.3	9530	NA	NA	54892984	0.969	0.946
R.327	High	R.327	1710000	10000	92.3	9230	NA	NA	45506291	0.968	0.954
R.327	Low	R.327	87900	10000	77.8	7780	NA	NA	50651212	0.963	0.954
R.327	Mid	R.327	237000	10000	87.2	8720	NA	NA	48611168	0.961	0.954
R.327	Blood	R.327	266000	10000	92.8	9280	NA	NA	51218066	0.968	0.954
R.328	High	R.328	956000	10000	88.8	8880	NA	NA	145055402	0.969	0.955
R.328	Low	R.328	365000	10000	96.3	9630	NA	NA	31227869	0.961	0.954
R.328	Mid	R.328	150000	10000	95.6	9560	NA	NA	41623868	0.96	0.954
R.328	Blood	R.328	322000	10000	93.9	9390	NA	NA	42277960	0.965	0.954

NHP_Sample_ID	Sample_Type	NHP_Donor_ID	Q30 Bases in RNA Read 1	Q30 Bases in UMI	Reads Mapped to Any V(D)J Gene	Reads Mapped to TRA	Reads Mapped to TRB	Mean Used Read Pairs per Cell	Fraction Reads in Cells	Median TRA UMIs per Cell	Median TRB UMIs per Cell
R.227	Liver	R.276	0.917	0.953	0.768	0.548	0.216	59111	0.42	4	12
R.227	Spleen	R.276	0.918	0.953	0.783	0.568	0.21	49444	0.514	3	9
R.276	Liver	R.279	0.916	0.953	0.773	0.516	0.25	23040	0.487	9	13
R.276	MLR	R.276	0.917	0.952	0.801	0.573	0.222	69955	0.475	14	18
R.276	Spleen	R.279	0.922	0.953	0.752	0.508	0.231	33812	0.474	5	9
R.276	Blood	R.276	0.921	0.953	0.739	0.492	0.243	66937	0.418	1	5
R.279	CD3	R.279	0.917	0.953	0.744	0.488	0.252	19948	0.627	18	19
R.279	MLR	R.279	0.919	0.953	0.769	0.554	0.21	46683	0.502	4	8
R.279	Blood	R.279	0.921	0.953	0.731	0.505	0.223	43039	0.52	2	7
R.312	Spleen	R.319	0.918	0.954	0.776	0.534	0.238	18615	0.499	6	9
R.319	CD3	R.319	0.922	0.954	0.755	0.529	0.216	61607	0.654	13	14
R.319	MLR	R.319	0.917	0.953	0.785	0.544	0.239	21930	0.395	20	28
R.319	Blood	R.319	0.918	0.953	0.771	0.517	0.25	12083	0.444	2	6
R.312	Liver	R.319	0.895	0.953	0.88	0.731	0.148	6032	0.729	12	12
R.329	High	R.329	0.885	0.942	0.773	0.616	0.155	5602	0.561	5	9
R.329	Low	R.329	0.894	0.942	0.763	0.629	0.131	3815	0.693	18	27
R.329	Mid	R.329	0.887	0.942	0.769	0.624	0.141	15003	0.635	14	28
R.329	Blood	R.329	0.894	0.942	0.786	0.63	0.154	2621	0.553	4	8
R.330	High	R.330	0.893	0.942	0.789	0.639	0.147	3206	0.613	6	8
R.330	Low	R.330	0.901	0.942	0.761	0.633	0.124	2817	0.572	26	23
R.330	Mid	R.330	0.898	0.942	0.74	0.616	0.119	5305	0.594	16	23
R.330	Blood	R.330	0.892	0.942	0.754	0.567	0.18	4069	0.564	6	10
R.327	High	R.327	0.893	0.951	0.804	0.645	0.157	2911	0.637	8	6
R.327	Low	R.327	0.895	0.951	0.751	0.622	0.127	1020	0.478	23	0
R.327	Mid	R.327	0.91	0.951	0.756	0.622	0.13	3612	0.612	22	14
R.327	Blood	R.327	0.895	0.951	0.77	0.592	0.176	3361	0.653	6	8
R.328	High	R.328	0.905	0.951	0.799	0.643	0.154	12747	0.691	7	9
R.328	Low	R.328	0.903	0.951	0.775	0.658	0.115	1919	0.526	44	17
R.328	Mid	R.328	0.902	0.951	0.757	0.646	0.107	6399	0.566	32	24
R.328	Blood	R.328	0.899	0.951	0.774	0.6	0.171	2888	0.58	6	8

NHP_Sample_ID	Sample_Type	NHP_Donor_ID	Cells With Productive V-J Spanning Pair	Cells With Productive V-J Spanning (TRA, TRB) Pair	Paired Clonotype Diversity	Cells With TRA Contig	Cells With TRB Contig	Cells With CDR3-annotated TRA Contig	Cells With CDR3-annotated TRB Contig
R.227	Liver	R.276	0.351	0.351	195.21	0.62	0.95	0.55	0.926
R.227	Spleen	R.276	0.37	0.37	150.1	0.651	0.904	0.578	0.878
R.276	Liver	R.279	0.47	0.47	504.48	0.844	0.877	0.786	0.848
R.276	MLR	R.276	0.474	0.474	91.42	0.839	0.919	0.754	0.886
R.276	Spleen	R.279	0.398	0.398	250.63	0.729	0.864	0.688	0.843
R.276	Blood	R.276	0.328	0.328	115.71	0.572	0.95	0.5	0.922
R.279	CD3	R.279	0.81	0.81	4.72	0.982	0.936	0.968	0.914
R.279	MLR	R.279	0.406	0.406	49.05	0.661	0.888	0.614	0.877
R.279	Blood	R.279	0.47	0.47	9.96	0.64	0.946	0.591	0.941
R.312	Spleen	R.319	0.437	0.437	1715.91	0.77	0.854	0.723	0.829
R.319	CD3	R.319	0.664	0.664	17.73	0.933	0.901	0.922	0.871
R.319	MLR	R.319	0.563	0.563	730.1	0.964	0.822	0.938	0.794
R.319	Blood	R.319	0.391	0.391	32.1	0.703	0.859	0.673	0.837
R.312	Liver	R.319	0.502	0.502	517.86	0.991	0.637	0.986	0.614
R.329	High	R.329	0.464	0.464	285.78	0.85	0.85	0.802	0.825
R.329	Low	R.329	0.591	0.591	1476.54	0.984	0.838	0.963	0.808
R.329	Mid	R.329	0.549	0.549	1181.73	0.931	0.954	0.847	0.935
R.329	Blood	R.329	0.422	0.422	36.53	0.814	0.85	0.758	0.834
R.330	High	R.330	0.485	0.485	308.02	0.885	0.795	0.858	0.777
R.330	Low	R.330	0.627	0.627	892.01	0.994	0.811	0.986	0.787
R.330	Mid	R.330	0.649	0.649	148.32	0.975	0.867	0.956	0.851
R.330	Blood	R.330	0.474	0.474	21.58	0.865	0.892	0.845	0.877
R.327	High	R.327	0.45	0.45	1267.68	0.981	0.641	0.972	0.616
R.327	Low	R.327	0.192	0.192	6630.87	0.999	0.324	0.998	0.296
R.327	Mid	R.327	0.464	0.464	4464.99	0.993	0.65	0.986	0.608
R.327	Blood	R.327	0.527	0.527	282.92	0.954	0.742	0.937	0.707
R.328	High	R.328	0.56	0.56	1799.11	0.914	0.883	0.88	0.861
R.328	Low	R.328	0.503	0.503	1209.91	0.997	0.66	0.995	0.631
R.328	Mid	R.328	0.625	0.625	2013.82	0.985	0.801	0.972	0.776
R.328	Blood	R.328	0.385	0.385	254.87	0.941	0.679	0.927	0.651

NHP_Sample_ID	Sample_Type	NHP_Donor_ID	Cells With V-J Spanning TRA Contig	Cells With V-J Spanning TRB Contig	Cells With Productive TRA Contig	Cells With Productive TRB Contig	Number of Unique CDR3 Alpha Chain Regions Identified
R.227	Liver	R.276	0.579	0.934	0.459	0.893	112
R.227	Spleen	R.276	0.61	0.888	0.513	0.857	252
R.276	Liver	R.279	0.813	0.855	0.657	0.814	984
R.276	MLR	R.276	0.787	0.886	0.654	0.82	123
R.276	Spleen	R.279	0.703	0.849	0.588	0.81	582
R.276	Blood	R.276	0.55	0.933	0.433	0.894	66
R.279	CD3	R.279	0.975	0.918	0.922	0.888	381
R.279	MLR	R.279	0.629	0.875	0.556	0.85	135
R.279	Blood	R.279	0.621	0.941	0.538	0.932	95
R.312	Spleen	R.319	0.745	0.835	0.644	0.793	1535
R.319	CD3	R.319	0.919	0.887	0.815	0.849	63
R.319	MLR	R.319	0.952	0.797	0.833	0.73	1004
R.319	Blood	R.319	0.692	0.846	0.576	0.815	703
R.312	Liver	R.319	0.988	0.622	0.958	0.544	2309
R.329	High	R.329	0.828	0.827	0.697	0.767	2679
R.329	Low	R.329	0.975	0.811	0.867	0.724	1986
R.329	Mid	R.329	0.898	0.937	0.673	0.876	1119
R.329	Blood	R.329	0.79	0.835	0.654	0.769	1371
R.330	High	R.330	0.858	0.781	0.764	0.721	4323
R.330	Low	R.330	0.991	0.789	0.93	0.697	1978
R.330	Mid	R.330	0.962	0.853	0.867	0.781	2611
R.330	Blood	R.330	0.731	0.881	0.635	0.839	729
R.327	High	R.327	0.975	0.622	0.934	0.516	7257
R.327	Low	R.327	0.998	0.303	0.993	0.2	5230
R.327	Mid	R.327	0.988	0.618	0.956	0.508	5717
R.327	Blood	R.327	0.945	0.717	0.886	0.64	6019
R.328	High	R.328	0.877	0.865	0.751	0.808	5049
R.328	Low	R.328	0.995	0.637	0.974	0.529	1747
R.328	Mid	R.328	0.977	0.78	0.918	0.707	2470
R.328	Blood	R.328	0.891	0.655	0.828	0.557	5066

**NHP_Sample_ID**	**Sample_Type**	**NHP_Donor_ID**	**Number of Unique CDR3 Beta Chain Regions Identified**	**Shannon Diversity**
R.227	Liver	R.276	196	NA
R.227	Spleen	R.276	409	NA
R.276	Liver	R.279	1184	NA
R.276	MLR	R.276	145	NA
R.276	Spleen	R.279	693	NA
R.276	Blood	R.276	142	NA
R.279	CD3	R.279	403	NA
R.279	MLR	R.279	226	NA
R.279	Blood	R.279	201	NA
R.312	Spleen	R.319	1865	NA
R.319	CD3	R.319	63	NA
R.319	MLR	R.319	829	NA
R.319	Blood	R.319	846	NA
R.312	Liver	R.319	1341	NA
R.329	High	R.329	2902	7.23
R.329	Low	R.329	1745	7.16
R.329	Mid	R.329	1401	6.90
R.329	Blood	R.329	1606	NA
R.330	High	R.330	3896	7.44
R.330	Low	R.330	1658	7.00
R.330	Mid	R.330	2310	6.92
R.330	Blood	R.330	848	NA
R.327	High	R.327	3784	8.50
R.327	Low	R.327	2076	8.07
R.327	Mid	R.327	3024	8.49
R.327	Blood	R.327	4174	NA
R.328	High	R.328	5174	8.44
R.328	Low	R.328	1318	6.93
R.328	Mid	R.328	1847	7.68
R.328	Blood	R.328	2888	NA

**NHP_Sample_ID**	ID of the NHP from which the sample was obtained.
**Sample_Type**	Organ or assay from which the sample was obtained.
**NHP_Donor_ID**	ID of the NHP from which the T cells originated.
**Cells_Loaded**	Number of cells loaded into chromium controller.
**Expected_Recovery_If_All_Viable**	Expected number of cells/barcodes that would be recovered in single cell analysis, assuming all are viable.
**Percent Viable**	Estimated percentage of cells in sample that are alive.
**Expected_Cells**	Expected_Recovery_If_All_Viable * Percent Viable
**GEX_Cell_Count_Before_Filtering**	Total number of GEX barcodes found, before any QC or filtering.
**GEX_Cell_Count_After_Filtering**	Total number of GEX barcodes after QC and filtering.
**Cellranger VDJ Statistics**	Summary statistics provided by cellranger VDJ program. Please see https://support.10xgenomics.com/single-cell-vdj/software/pipelines/latest/output/metrics for details.
**Shannon Diversity**	Calculated by binning T cells into clones by nuclotide sequence of alpha chain CDR3

Supplementary Table S3: Differential Expression MLR+ vs MLR-

Test	Gene	Adjusted P Value	P Value	Average Log2 FC	MLR+ Cells Expressing Gene	Cells Expressing Gene
R.279 Liver MLR+ vs MLR-	ENSMMUG00000019371	0.000	0.000	0.41	0.362	0.048
R.279 Liver MLR+ vs MLR-	CX3CR1	0.000	0.000	0.37	0.276	0.037
R.279 Liver MLR+ vs MLR-	CAPG	0.000	0.000	0.91	0.638	0.218
R.279 Liver MLR+ vs MLR-	MAMU-DRA	0.000	0.000	0.76	0.345	0.076
R.279 Liver MLR+ vs MLR-	MAMU-DRB1	0.000	0.000	0.86	0.534	0.179
R.279 Liver MLR+ vs MLR-	IL7R	0.000	0.000	0.79	0.586	0.205
R.279 Liver MLR+ vs MLR-	DHRS7	0.000	0.000	0.66	0.621	0.252
R.279 Liver MLR+ vs MLR-	PPP2R2B	0.000	0.000	0.35	0.259	0.056
R.279 Liver MLR+ vs MLR-	S100A10	0.000	0.000	0.83	0.983	0.866
R.279 Liver MLR+ vs MLR-	BCL2A1	0.000	0.000	0.87	0.879	0.613
R.279 Liver MLR+ vs MLR-	ANXA1	0.000	0.000	1.09	0.776	0.475
R.279 Liver MLR+ vs MLR-	ENSMMUG00000060606	0.000	0.000	0.43	0.207	0.04
R.279 Liver MLR+ vs MLR-	CDKN2B	0.000	0.000	0.26	0.259	0.061
R.279 Liver MLR+ vs MLR-	LAG3	0.000	0.000	-1.16	0.207	0.575
R.279 Liver MLR+ vs MLR-	B2M	0.000	0.000	0.35	1	0.999
R.279 Liver MLR+ vs MLR-	CD52	0.000	0.000	0.57	0.983	0.945
R.279 Liver MLR+ vs MLR-	ENSMMUG00000061119	0.000	0.000	0.61	0.241	0.059
R.279 Liver MLR+ vs MLR-	SPOCK2	0.000	0.000	-0.73	0.086	0.527
R.279 Liver MLR+ vs MLR-	ENSMMUG00000056183	0.000	0.000	0.26	0.172	0.034
R.279 Liver MLR+ vs MLR-	GZMA	0.000	0.000	-2.62	0.569	0.752
R.279 Liver MLR+ vs MLR-	KLRD1	0.000	0.000	0.63	0.741	0.409
R.279 Liver MLR+ vs MLR-	PRF1	0.000	0.000	-1.34	0.517	0.728
R.279 Liver MLR+ vs MLR-	EFHD2	0.000	0.000	0.69	0.776	0.5
R.279 Liver MLR+ vs MLR-	MATK	0.001	0.000	0.25	0.293	0.086
R.279 Liver MLR+ vs MLR-	CD74	0.001	0.000	1.15	0.81	0.507
R.279 Liver MLR+ vs MLR-	ENSMMUG00000060287	0.001	0.000	0.41	0.983	0.989
R.279 Liver MLR+ vs MLR-	SERPINA1	0.002	0.000	0.60	0.345	0.122
R.279 Liver MLR+ vs MLR-	ENSMMUG00000064139	0.003	0.000	0.42	1	0.997
R.279 Liver MLR+ vs MLR-	PLIN2	0.003	0.000	0.56	0.845	0.614
R.279 Liver MLR+ vs MLR-	RPL12	0.003	0.000	-0.48	0.983	0.996
R.279 Liver MLR+ vs MLR-	CD96	0.003	0.000	0.37	0.466	0.192
R.279 Liver MLR+ vs MLR-	TESC	0.004	0.000	0.47	0.69	0.393
R.279 Liver MLR+ vs MLR-	RPS27L	0.005	0.000	0.65	0.741	0.538
R.279 Liver MLR+ vs MLR-	ENSMMUG00000065017	0.012	0.000	0.46	0.138	0.028
R.279 Liver MLR+ vs MLR-	FAM3C	0.015	0.000	-0.73	0.017	0.323
R.279 Liver MLR+ vs MLR-	CD7	0.016	0.000	-0.91	0.19	0.509
R.279 Liver MLR+ vs MLR-	TAPBPL	0.016	0.000	-0.62	0.448	0.727
R.279 Liver MLR+ vs MLR-	SCML4	0.024	0.000	0.48	0.534	0.255
R.279 Liver MLR+ vs MLR-	CD6	0.040	0.000	0.51	0.534	0.286
R.279 Liver MLR+ vs MLR-	UBASH3A	0.048	0.000	0.25	0.362	0.142
R.279 Liver MLR+ vs MLR-	WTAP	0.067	0.000	0.37	0.724	0.446
R.279 Liver MLR+ vs MLR-	AQR	0.175	0.000	0.27	0.328	0.133
R.279 Liver MLR+ vs MLR-	UBASH3B	0.227	0.000	-0.47	0.121	0.393
R.279 Liver MLR+ vs MLR-	TUBA4A	0.229	0.000	0.51	0.724	0.487
R.279 Liver MLR+ vs MLR-	FYB1	0.257	0.000	0.43	0.793	0.582
R.279 Liver MLR+ vs MLR-	CTLA4	0.294	0.000	-0.69	0.138	0.412
R.279 Liver MLR+ vs MLR-	EML4	0.323	0.000	0.47	0.69	0.471
R.279 Liver MLR+ vs MLR-	CXCR4	0.370	0.000	0.65	0.793	0.613

R.279 Liver MLR+ vs MLR-	RAP1B	0.428	0.000	0.43	1	0.802
R.279 Liver MLR+ vs MLR-	CD99	0.459	0.000	0.44	0.879	0.651
R.279 Liver MLR+ vs MLR-	ENSMMUG00000004441	0.490	0.000	0.44	1	0.918
R.279 Liver MLR+ vs MLR-	PCED1B	0.512	0.000	-0.45	0.069	0.322
R.279 Liver MLR+ vs MLR-	SLAMF7	0.540	0.000	0.29	0.414	0.197
R.279 Liver MLR+ vs MLR-	TMSB10	0.546	0.000	0.37	1	0.997
R.279 Liver MLR+ vs MLR-	KLF3	0.596	0.000	0.30	0.293	0.12
R.279 Liver MLR+ vs MLR-	ENTPD1	0.642	0.000	-0.42	0.017	0.252
R.279 Liver MLR+ vs MLR-	CCNL1	0.684	0.000	0.45	0.862	0.663
R.279 Liver MLR+ vs MLR-	BTG1	0.747	0.000	0.47	0.897	0.75
R.279 Liver MLR+ vs MLR-	RGS9	1.000	0.000	0.39	0.517	0.294
R.279 Liver MLR+ vs MLR-	IFI6	1.000	0.000	0.52	0.793	0.624
R.279 Liver MLR+ vs MLR-	PIK3AP1	1.000	0.000	-0.38	0.052	0.287
R.279 Liver MLR+ vs MLR-	UPP1	1.000	0.000	-0.41	0.086	0.331
R.279 Liver MLR+ vs MLR-	FGL2	1.000	0.000	0.28	0.259	0.103
R.279 Liver MLR+ vs MLR-	LITAF	1.000	0.000	-0.58	0.19	0.431
R.279 Liver MLR+ vs MLR-	CD38	1.000	0.000	-0.52	0.31	0.551
R.279 Liver MLR+ vs MLR-	C15orf48	1.000	0.000	0.26	0.345	0.157
R.279 Liver MLR+ vs MLR-	STAMBPL1	1.000	0.000	0.41	0.569	0.352
R.279 Liver MLR+ vs MLR-	PLEK	1.000	0.000	0.26	0.328	0.151
R.279 Liver MLR+ vs MLR-	ETS2	1.000	0.000	0.44	0.345	0.172
R.279 Liver MLR+ vs MLR-	ATF3	1.000	0.000	0.34	0.534	0.302
R.279 Liver MLR+ vs MLR-	ANXA5	1.000	0.000	0.37	0.534	0.31
R.279 Liver MLR+ vs MLR-	TAGAP	1.000	0.000	0.31	0.69	0.438
R.279 Liver MLR+ vs MLR-	RPL28	1.000	0.000	-0.32	1	0.993
R.279 Liver MLR+ vs MLR-	UBE2F	1.000	0.000	-0.41	0.121	0.352
R.279 Liver MLR+ vs MLR-	CMA1	1.000	0.000	0.34	0.328	0.159
R.279 Liver MLR+ vs MLR-	DUSP16	1.000	0.000	-0.33	0.017	0.213
R.279 Liver MLR+ vs MLR-	ENSMMUG00000064120	1.000	0.000	0.26	1	0.993
R.279 Liver MLR+ vs MLR-	RAB7A	1.000	0.000	0.29	0.603	0.377
R.279 Liver MLR+ vs MLR-	MAMU-F	1.000	0.000	0.31	0.379	0.198
R.279 Liver MLR+ vs MLR-	SQOR	1.000	0.000	0.26	0.31	0.15
R.279 Liver MLR+ vs MLR-	ENSMMUG00000050862	1.000	0.000	-0.77	0.069	0.282
R.279 Liver MLR+ vs MLR-	RPS12	1.000	0.000	-0.28	1	0.995
R.279 Liver MLR+ vs MLR-	AHNAK	1.000	0.000	0.63	0.655	0.512
R.279 Liver MLR+ vs MLR-	IFI27L2	1.000	0.000	0.28	0.948	0.849
R.279 Liver MLR+ vs MLR-	RPS5	1.000	0.000	-0.26	1	0.993
R.279 Liver MLR+ vs MLR-	RNF19B	1.000	0.000	0.26	0.414	0.22
R.279 Liver MLR+ vs MLR-	EMP3	1.000	0.000	0.38	0.914	0.825
R.279 Liver MLR+ vs MLR-	PDCD1	1.000	0.000	-0.50	0.155	0.364
R.279 Liver MLR+ vs MLR-	LCP1	1.000	0.000	0.32	0.931	0.849
R.279 Liver MLR+ vs MLR-	ARL6IP5	1.000	0.001	0.27	0.724	0.475
R.279 Liver MLR+ vs MLR-	PPP1R15A	1.000	0.001	0.43	0.862	0.696
R.279 Liver MLR+ vs MLR-	RPL37A	1.000	0.001	-0.26	1	0.994
R.279 Liver MLR+ vs MLR-	ENSMMUG00000039070	1.000	0.001	-0.36	0.155	0.382
R.279 Liver MLR+ vs MLR-	ANTXR2	1.000	0.001	0.31	0.19	0.075
R.279 Liver MLR+ vs MLR-	STK39	1.000	0.001	0.25	0.5	0.299
R.279 Liver MLR+ vs MLR-	NTRK1	1.000	0.001	-0.32	0	0.167
R.279 Liver MLR+ vs MLR-	NR3C1	1.000	0.001	0.29	0.379	0.208
R.279 Liver MLR+ vs MLR-	ENO1	1.000	0.001	-0.58	0.759	0.852
R.279 Liver MLR+ vs MLR-	ABRACL	1.000	0.001	0.31	0.845	0.668
R.279 Liver MLR+ vs MLR-	TIGAR	1.000	0.001	-0.46	0.345	0.54

R.279 Liver MLR+ vs MLR-	NEDD9	1.000	0.001	0.34	0.707	0.528
R.279 Liver MLR+ vs MLR-	ENSMMUG00000064232	1.000	0.001	0.36	0.517	0.336
R.279 Liver MLR+ vs MLR-	MYADM	1.000	0.001	0.35	0.552	0.363
R.279 Liver MLR+ vs MLR-	IFI27	1.000	0.001	0.53	0.69	0.578
R.279 Liver MLR+ vs MLR-	ENSMMUG00000058581	1.000	0.001	0.47	0.845	0.641
R.279 Liver MLR+ vs MLR-	RACK1	1.000	0.001	-0.29	1	0.991
R.279 Liver MLR+ vs MLR-	COA1	1.000	0.001	0.42	0.397	0.236
R.279 Liver MLR+ vs MLR-	IGF2R	1.000	0.001	-0.38	0.121	0.315
R.279 Liver MLR+ vs MLR-	SH2D1A	1.000	0.001	0.29	0.414	0.24
R.279 Liver MLR+ vs MLR-	IL2RA	1.000	0.001	-0.70	0.138	0.334
R.279 Liver MLR+ vs MLR-	AKR1B1	1.000	0.001	-0.26	0.069	0.259
R.279 Liver MLR+ vs MLR-	HAVCR2	1.000	0.001	-0.28	0.034	0.207
R.279 Liver MLR+ vs MLR-	HSD17B4	1.000	0.001	0.26	0.431	0.252
R.279 Liver MLR+ vs MLR-	HDLBP	1.000	0.001	-0.34	0.138	0.334
R.279 Liver MLR+ vs MLR-	LGALS3	1.000	0.001	0.47	0.828	0.708
R.279 Liver MLR+ vs MLR-	PPP1R12A	1.000	0.002	0.31	0.621	0.417
R.279 Liver MLR+ vs MLR-	SRSF7	1.000	0.002	0.33	0.793	0.696
R.279 Liver MLR+ vs MLR-	MYO1E	1.000	0.002	-0.34	0.069	0.243
R.279 Liver MLR+ vs MLR-	H2AJ	1.000	0.002	-0.35	0.5	0.672
R.279 Liver MLR+ vs MLR-	TNFRSF1B	1.000	0.002	-0.45	0.638	0.755
R.279 Liver MLR+ vs MLR-	ZFP36	1.000	0.002	0.38	0.914	0.772
R.279 Liver MLR+ vs MLR-	CRIP1	1.000	0.002	0.42	0.983	0.952
R.279 Liver MLR+ vs MLR-	SLBP	1.000	0.002	0.30	0.586	0.408
R.279 Liver MLR+ vs MLR-	CDKN1A	1.000	0.002	0.39	0.552	0.364
R.279 Liver MLR+ vs MLR-	PRDX6	1.000	0.002	0.34	0.879	0.763
R.279 Liver MLR+ vs MLR-	BATF	1.000	0.002	0.25	0.517	0.319
R.279 Liver MLR+ vs MLR-	MRPL4	1.000	0.002	-0.26	0.069	0.241
R.279 Liver MLR+ vs MLR-	SSR1	1.000	0.002	-0.30	0.19	0.386
R.279 Liver MLR+ vs MLR-	STAT3	1.000	0.002	-0.33	0.19	0.382
R.279 Liver MLR+ vs MLR-	SERPINE2	1.000	0.002	-0.45	0	0.14
R.279 Liver MLR+ vs MLR-	CLTC	1.000	0.002	0.26	0.466	0.3
R.279 Liver MLR+ vs MLR-	USP3	1.000	0.002	0.31	0.345	0.197
R.279 Liver MLR+ vs MLR-	CCL5	1.000	0.002	0.40	0.862	0.706
R.279 Liver MLR+ vs MLR-	ENSMMUG00000003532	1.000	0.002	-0.47	0.448	0.643
R.279 Liver MLR+ vs MLR-	ARRB2	1.000	0.002	0.34	0.69	0.533
R.279 Liver MLR+ vs MLR-	ALOX5AP	1.000	0.002	0.27	0.241	0.114
R.279 Liver MLR+ vs MLR-	PLAC8	1.000	0.003	-0.67	0.259	0.437
R.279 Liver MLR+ vs MLR-	SERTAD1	1.000	0.003	0.36	0.724	0.568
R.279 Liver MLR+ vs MLR-	LMO4	1.000	0.003	-0.39	0.155	0.329
R.279 Liver MLR+ vs MLR-	IL4R	1.000	0.003	-0.27	0.207	0.418
R.279 Liver MLR+ vs MLR-	KIR2DL4	1.000	0.003	-0.42	0.017	0.158
R.279 Liver MLR+ vs MLR-	PTPN7	1.000	0.003	-0.27	0.121	0.303
R.279 Liver MLR+ vs MLR-	S100A4	1.000	0.003	0.40	0.983	0.93
R.279 Liver MLR+ vs MLR-	ENSMMUG00000053735	1.000	0.003	-0.32	0.293	0.476
R.279 Liver MLR+ vs MLR-	CA6	1.000	0.003	-0.35	0.086	0.251
R.279 Liver MLR+ vs MLR-	IDH2	1.000	0.003	0.43	0.69	0.628
R.279 Liver MLR+ vs MLR-	TIMM8B	1.000	0.003	-0.25	0.069	0.229
R.279 Liver MLR+ vs MLR-	TNFSF11	1.000	0.004	-0.29	0	0.129
R.279 Liver MLR+ vs MLR-	CD48	1.000	0.004	0.35	0.983	0.891
R.279 Liver MLR+ vs MLR-	EIF5B	1.000	0.004	-0.27	0.103	0.269
R.279 Liver MLR+ vs MLR-	RNF181	1.000	0.004	-0.28	0.241	0.419
R.279 Liver MLR+ vs MLR-	SQSTM1	1.000	0.004	0.34	0.862	0.656

R.279 Liver MLR+ vs MLR-	NKG7	1.000	0.004	0.27	0.966	0.894
R.279 Liver MLR+ vs MLR-	JUND	1.000	0.004	0.26	0.741	0.561
R.279 Liver MLR+ vs MLR-	OSBPL2	1.000	0.004	0.28	0.672	0.515
R.279 Liver MLR+ vs MLR-	ITM2A	1.000	0.004	0.26	0.448	0.293
R.279 Liver MLR+ vs MLR-	LYST	1.000	0.005	-0.30	0.172	0.344
R.279 Liver MLR+ vs MLR-	TBC1D25	1.000	0.005	0.29	0.5	0.338
R.279 Liver MLR+ vs MLR-	MGST3	1.000	0.005	0.27	0.414	0.256
R.279 Liver MLR+ vs MLR-	PLAAT4	1.000	0.005	0.50	0.776	0.671
R.279 Liver MLR+ vs MLR-	PMAIP1	1.000	0.005	0.35	0.621	0.458
R.279 Liver MLR+ vs MLR-	ISG20	1.000	0.005	0.33	0.862	0.67
R.279 Liver MLR+ vs MLR-	PSMB9	1.000	0.005	0.33	0.897	0.842
R.279 Liver MLR+ vs MLR-	ENSMMUG00000014786	1.000	0.005	-0.27	0.983	0.989
R.279 Liver MLR+ vs MLR-	SUB1	1.000	0.005	0.38	0.776	0.675
R.279 Liver MLR+ vs MLR-	RGS2	1.000	0.005	-0.26	0.155	0.345
R.279 Liver MLR+ vs MLR-	SRSF5	1.000	0.005	0.27	0.879	0.701
R.279 Liver MLR+ vs MLR-	GNA13	1.000	0.005	0.32	0.534	0.401
R.279 Liver MLR+ vs MLR-	ENSMMUG00000053539	1.000	0.006	0.26	0.379	0.222
R.279 Liver MLR+ vs MLR-	ENSMMUG00000045208	1.000	0.006	0.25	0.862	0.78
R.279 Liver MLR+ vs MLR-	PGK1	1.000	0.006	-0.36	0.569	0.682
R.279 Liver MLR+ vs MLR-	CLTB	1.000	0.006	-0.26	0.138	0.297
R.279 Liver MLR+ vs MLR-	NUDC	1.000	0.007	-0.34	0.397	0.535
R.279 Liver MLR+ vs MLR-	PARK7	1.000	0.007	-0.28	0.345	0.508
R.279 Liver MLR+ vs MLR-	HDAC7	1.000	0.007	-0.26	0.121	0.275
R.279 Liver MLR+ vs MLR-	ARF1	1.000	0.007	0.29	0.862	0.81
R.279 Liver MLR+ vs MLR-	PELI1	1.000	0.007	-0.35	0.293	0.456
R.279 Liver MLR+ vs MLR-	DUSP4	1.000	0.007	-0.26	0.138	0.307
R.279 Liver MLR+ vs MLR-	ENSMMUG00000013779	1.000	0.007	-0.52	0	0.113
R.279 Liver MLR+ vs MLR-	GLIPR2	1.000	0.007	0.27	0.621	0.47
R.279 Liver MLR+ vs MLR-	RPL4	1.000	0.007	-0.25	0.966	0.983
R.279 Liver MLR+ vs MLR-	TSC22D3	1.000	0.007	0.35	0.828	0.738
R.279 Liver MLR+ vs MLR-	FLNA	1.000	0.007	0.48	0.707	0.624
R.279 Liver MLR+ vs MLR-	FNBP1	1.000	0.007	0.29	0.759	0.601
R.279 Liver MLR+ vs MLR-	PDE4B	1.000	0.007	0.28	0.517	0.349
R.279 Liver MLR+ vs MLR-	RASSF5	1.000	0.007	0.26	0.534	0.386
R.279 Liver MLR+ vs MLR-	ANXA6	1.000	0.007	-0.33	0.448	0.6
R.279 Liver MLR+ vs MLR-	GSTP1	1.000	0.007	-0.27	0.931	0.925
R.279 Liver MLR+ vs MLR-	EMC3	1.000	0.007	0.26	0.397	0.258
R.279 Liver MLR+ vs MLR-	PSME1	1.000	0.007	0.26	0.931	0.919
R.279 Liver MLR+ vs MLR-	AHI1	1.000	0.008	-0.34	0.241	0.392
R.279 Liver MLR+ vs MLR-	TTC39C	1.000	0.008	0.25	0.397	0.249
R.279 Liver MLR+ vs MLR-	ITGB7	1.000	0.008	0.26	0.483	0.323
R.279 Liver MLR+ vs MLR-	ATP5F1D	1.000	0.008	-0.26	0.828	0.844
R.279 Liver MLR+ vs MLR-	PEBP1	1.000	0.008	-0.28	0.379	0.566
R.279 Liver MLR+ vs MLR-	ZC3H10	1.000	0.008	-0.30	0.845	0.894
R.279 Liver MLR+ vs MLR-	CCDC85B	1.000	0.009	-0.28	0.224	0.381
R.279 Liver MLR+ vs MLR-	APBB1IP	1.000	0.009	0.26	0.672	0.481
R.279 Liver MLR+ vs MLR-	MIF	1.000	0.009	-0.38	0.759	0.803
R.279 Liver MLR+ vs MLR-	ENSMMUG00000061128	1.000	0.009	0.26	0.776	0.582
R.279 Liver MLR+ vs MLR-	ENSMMUG00000006499	1.000	0.009	-0.26	0.19	0.347
R.279 Liver MLR+ vs MLR-	CD37	1.000	0.010	0.28	0.707	0.559
R.279 Liver MLR+ vs MLR-	DNAJB6	1.000	0.010	0.26	0.621	0.48
R.279 Liver MLR+ vs MLR-	ENSMMUG00000043332	1.000	0.010	0.25	0.81	0.631

R.279 Liver MLR+ vs MLR-	ICOS	1.000	0.010	-0.32	0.207	0.369
R.279 Liver MLR+ vs MLR-	IRF8	1.000	0.010	-0.33	0.259	0.422
R.279 Liver MLR+ vs MLR-	TANK	1.000	0.011	0.27	0.397	0.257
R.279 Liver MLR+ vs MLR-	XCL1	1.000	0.011	-0.75	0.034	0.151
R.279 Liver MLR+ vs MLR-	DNAJC3	1.000	0.012	-0.26	0.276	0.433
R.279 Liver MLR+ vs MLR-	SLC25A4	1.000	0.012	0.27	0.466	0.332
R.279 Liver MLR+ vs MLR-	NDUFB9	1.000	0.012	-0.30	0.448	0.565
R.279 Liver MLR+ vs MLR-	CD8A	1.000	0.013	-0.36	0.672	0.766
R.279 Liver MLR+ vs MLR-	TUBB2B	1.000	0.013	0.38	0.638	0.545
R.279 Liver MLR+ vs MLR-	PARP6	1.000	0.014	-0.36	0.466	0.57
R.279 Liver MLR+ vs MLR-	PTGER4	1.000	0.014	0.33	0.414	0.279
R.279 Liver MLR+ vs MLR-	GBP3	1.000	0.014	0.34	0.69	0.552
R.279 Liver MLR+ vs MLR-	GATA3	1.000	0.014	-0.29	0.172	0.318
R.279 Liver MLR+ vs MLR-	ENSMMUG00000003854	1.000	0.014	0.29	0.655	0.517
R.279 Liver MLR+ vs MLR-	CLEC2D	1.000	0.015	0.26	0.914	0.737
R.279 Liver MLR+ vs MLR-	PDIA6	1.000	0.015	-0.34	0.345	0.473
R.279 Liver MLR+ vs MLR-	MRPL10	1.000	0.016	0.26	0.448	0.325
R.279 Liver MLR+ vs MLR-	ZYX	1.000	0.016	0.26	0.638	0.52
R.279 Liver MLR+ vs MLR-	RRAD	1.000	0.016	0.30	0.448	0.313
R.279 Liver MLR+ vs MLR-	OSR2	1.000	0.016	0.26	0.224	0.126
R.279 Liver MLR+ vs MLR-	HK3	1.000	0.017	-0.29	0.052	0.169
R.279 Liver MLR+ vs MLR-	COX3	1.000	0.019	-0.50	0.776	0.865
R.279 Liver MLR+ vs MLR-	NPC2	1.000	0.019	-0.26	0.069	0.187
R.279 Liver MLR+ vs MLR-	CD69	1.000	0.020	0.28	0.828	0.72
R.279 Liver MLR+ vs MLR-	PLK3	1.000	0.020	0.32	0.276	0.168
R.279 Liver MLR+ vs MLR-	LCP2	1.000	0.020	0.32	0.586	0.465
R.279 Liver MLR+ vs MLR-	CIB1	1.000	0.021	0.30	0.655	0.556
R.279 Liver MLR+ vs MLR-	COX1	1.000	0.021	-0.46	0.776	0.841
R.279 Liver MLR+ vs MLR-	C1QBP	1.000	0.021	-0.27	0.259	0.395
R.279 Liver MLR+ vs MLR-	IRF1	1.000	0.022	0.27	0.793	0.681
R.279 Liver MLR+ vs MLR-	ITGAX	1.000	0.022	-0.30	0.086	0.205
R.279 Liver MLR+ vs MLR-	ENSMMUG00000061125	1.000	0.022	-0.26	0.121	0.247
R.279 Liver MLR+ vs MLR-	CANX	1.000	0.023	-0.34	0.362	0.463
R.279 Liver MLR+ vs MLR-	PDIA4	1.000	0.023	-0.34	0.224	0.35
R.279 Liver MLR+ vs MLR-	IRF4	1.000	0.024	-0.38	0.293	0.413
R.279 Liver MLR+ vs MLR-	HTATSF1	1.000	0.024	0.26	0.362	0.249
R.279 Liver MLR+ vs MLR-	PPIB	1.000	0.025	-0.28	0.897	0.851
R.279 Liver MLR+ vs MLR-	FOS	1.000	0.025	0.28	0.828	0.653
R.279 Liver MLR+ vs MLR-	RGS1	1.000	0.025	0.33	0.724	0.61
R.279 Liver MLR+ vs MLR-	CD44	1.000	0.025	0.25	0.793	0.735
R.279 Liver MLR+ vs MLR-	PFKFB3	1.000	0.026	0.26	0.31	0.201
R.279 Liver MLR+ vs MLR-	PSMB3	1.000	0.027	-0.26	0.483	0.585
R.279 Liver MLR+ vs MLR-	GZMK	1.000	0.030	-0.26	0.345	0.52
R.279 Liver MLR+ vs MLR-	ANXA2	1.000	0.030	0.30	0.776	0.735
R.279 Liver MLR+ vs MLR-	SH3BGRL	1.000	0.032	0.25	0.5	0.363
R.279 Liver MLR+ vs MLR-	ENSMMUG00000057791	1.000	0.032	-0.34	0.017	0.101
R.279 Liver MLR+ vs MLR-	SERPINB1	1.000	0.033	-0.52	0.621	0.67
R.279 Liver MLR+ vs MLR-	ENSMMUG00000015272	1.000	0.034	-0.29	0.466	0.56
R.279 Liver MLR+ vs MLR-	THY1	1.000	0.037	-0.25	0.224	0.354
R.279 Liver MLR+ vs MLR-	TAF15	1.000	0.040	0.26	0.569	0.464
R.279 Liver MLR+ vs MLR-	BST2	1.000	0.040	-0.28	0.69	0.741
R.279 Liver MLR+ vs MLR-	H1FX	1.000	0.042	-0.28	0.276	0.397

R.279 Liver MLR+ vs MLR-	TBCC	1.000	0.044	0.35	0.5	0.414
R.279 Liver MLR+ vs MLR-	SH3KBP1	1.000	0.046	0.27	0.638	0.597
R.279 Liver MLR+ vs MLR-	SAMSN1	1.000	0.046	-0.29	0.31	0.404
R.279 Liver MLR+ vs MLR-	DBI	1.000	0.047	0.28	0.707	0.656
R.279 Liver MLR+ vs MLR-	ENSMMUG00000013256	1.000	0.048	0.27	0.655	0.549
R.279 Liver MLR+ vs MLR-	TXNIP	1.000	0.049	0.36	0.345	0.246
R.279 Liver MLR+ vs MLR-	GZMB	1.000	0.054	-0.62	0.897	0.871
R.279 Liver MLR+ vs MLR-	ENSMMUG00000056515	1.000	0.058	-0.79	0.086	0.176
R.279 Liver MLR+ vs MLR-	SPN	1.000	0.065	0.35	0.448	0.383
R.279 Liver MLR+ vs MLR-	KLF2	1.000	0.065	0.29	0.724	0.601
R.279 Liver MLR+ vs MLR-	SDF2L1	1.000	0.066	-0.26	0.31	0.41
R.279 Liver MLR+ vs MLR-	LSM14A	1.000	0.067	0.25	0.5	0.399
R.279 Liver MLR+ vs MLR-	JUNB	1.000	0.068	0.31	0.466	0.369
R.279 Liver MLR+ vs MLR-	TRAC	1.000	0.087	-0.27	0.655	0.663
R.279 Liver MLR+ vs MLR-	GPR183	1.000	0.091	-0.37	0.224	0.323
R.279 Liver MLR+ vs MLR-	ENSMMUG00000063583	1.000	0.094	-0.61	1	0.886
R.279 Liver MLR+ vs MLR-	ATP6	1.000	0.095	-0.39	0.862	0.878
R.279 Liver MLR+ vs MLR-	CGA	1.000	0.095	-0.61	0.086	0.163
R.279 Liver MLR+ vs MLR-	FABP5	1.000	0.099	0.30	0.345	0.456
R.279 Liver MLR+ vs MLR-	SELL	1.000	0.114	-0.27	0.086	0.158
R.279 Liver MLR+ vs MLR-	MT2A	1.000	0.116	0.26	0.483	0.6
R.279 Liver MLR+ vs MLR-	TPI1	1.000	0.138	-0.25	0.81	0.825
R.279 Liver MLR+ vs MLR-	CTSW	1.000	0.139	-0.36	0.293	0.358
R.279 Liver MLR+ vs MLR-	HSP90B1	1.000	0.153	-0.34	0.81	0.771
R.279 Liver MLR+ vs MLR-	GADD45B	1.000	0.155	0.47	0.621	0.564
R.279 Liver MLR+ vs MLR-	TIGIT	1.000	0.181	-0.28	0.5	0.539
R.279 Liver MLR+ vs MLR-	RGCC	1.000	0.187	0.38	0.793	0.759
R.279 Liver MLR+ vs MLR-	CCL3	1.000	0.191	-0.47	0.483	0.547
R.279 Liver MLR+ vs MLR-	JUN	1.000	0.194	0.35	0.828	0.79
R.279 Liver MLR+ vs MLR-	CTSC	1.000	0.212	-0.30	0.5	0.547
R.279 Liver MLR+ vs MLR-	PCLAF	1.000	0.217	0.25	0.31	0.252
R.279 Liver MLR+ vs MLR-	ENSMMUG00000062077	1.000	0.415	-1.21	1	0.999
R.279 Liver MLR+ vs MLR-	ENSMMUG00000051385	1.000	0.456	0.36	0.172	0.145
R.279 Liver MLR+ vs MLR-	COTL1	1.000	0.463	0.27	0.793	0.857
R.279 Liver MLR+ vs MLR-	TYROBP	1.000	0.469	-0.36	0.103	0.128
R.279 Liver MLR+ vs MLR-	ENSMMUG00000062894	1.000	0.496	-0.33	0.638	0.6
R.279 Liver MLR+ vs MLR-	ZFP36L1	1.000	0.557	0.28	0.569	0.577
R.279 Liver MLR+ vs MLR-	HIST2H3A	1.000	0.713	-0.33	0.138	0.155
R.279 Liver MLR+ vs MLR-	STMN1	1.000	0.751	0.43	0.483	0.519
R.279 Liver MLR+ vs MLR-	RRM2	1.000	0.802	0.27	0.328	0.333
R.279 Liver MLR+ vs MLR-	COX2	1.000	0.838	-0.28	0.741	0.754
R.279 Spleen MLR+ vs MLR-	CDKN2C	0.000	0.000	0.30	0.216	0.028
R.279 Spleen MLR+ vs MLR-	GOLIM4	0.000	0.000	0.42	0.432	0.103
R.279 Spleen MLR+ vs MLR-	CD96	0.000	0.000	0.50	0.595	0.192
R.279 Spleen MLR+ vs MLR-	CD7	0.000	0.000	-1.47	0.135	0.653
R.279 Spleen MLR+ vs MLR-	PPP2R2B	0.000	0.000	0.31	0.351	0.075
R.279 Spleen MLR+ vs MLR-	MAMU-DRB1	0.000	0.000	0.72	0.649	0.227
R.279 Spleen MLR+ vs MLR-	CLIC5	0.000	0.000	0.48	0.405	0.103
R.279 Spleen MLR+ vs MLR-	ENSMMUG00000065017	0.000	0.000	0.76	0.189	0.027
R.279 Spleen MLR+ vs MLR-	S100A10	0.000	0.000	0.98	0.946	0.821
R.279 Spleen MLR+ vs MLR-	S100A4	0.000	0.000	0.99	0.973	0.719
R.279 Spleen MLR+ vs MLR-	ENSMMUG00000019371	0.001	0.000	0.31	0.297	0.068

R.279 Spleen MLR+ vs MLR-	RPS27A.1	0.002	0.000	-0.58	1	0.996
R.279 Spleen MLR+ vs MLR-	NKG7	0.002	0.000	0.82	1	0.688
R.279 Spleen MLR+ vs MLR-	SH3BP5	0.002	0.000	0.54	0.486	0.167
R.279 Spleen MLR+ vs MLR-	TSPAN2	0.002	0.000	0.33	0.324	0.082
R.279 Spleen MLR+ vs MLR-	ANXA1	0.002	0.000	0.99	0.892	0.573
R.279 Spleen MLR+ vs MLR-	ENSMMUG00000052609	0.003	0.000	-0.61	1	0.995
R.279 Spleen MLR+ vs MLR-	EFHD2	0.004	0.000	0.83	0.811	0.49
R.279 Spleen MLR+ vs MLR-	ENSMMUG00000060382	0.005	0.000	0.48	0.459	0.157
R.279 Spleen MLR+ vs MLR-	RPS3A	0.007	0.000	-0.53	1	0.996
R.279 Spleen MLR+ vs MLR-	RPS13	0.012	0.000	-0.58	1	0.994
R.279 Spleen MLR+ vs MLR-	RGS9	0.013	0.000	0.53	0.676	0.305
R.279 Spleen MLR+ vs MLR-	SPOCK2	0.016	0.000	-0.97	0	0.413
R.279 Spleen MLR+ vs MLR-	HABP4	0.016	0.000	0.29	0.243	0.056
R.279 Spleen MLR+ vs MLR-	IL7R	0.032	0.000	0.90	0.811	0.439
R.279 Spleen MLR+ vs MLR-	CST7	0.035	0.000	0.60	0.973	0.616
R.279 Spleen MLR+ vs MLR-	CCL5	0.036	0.000	0.75	0.946	0.529
R.279 Spleen MLR+ vs MLR-	GZMB	0.039	0.000	0.75	0.838	0.489
R.279 Spleen MLR+ vs MLR-	ITGB2	0.045	0.000	0.58	0.946	0.688
R.279 Spleen MLR+ vs MLR-	SLCO4C1	0.048	0.000	0.26	0.189	0.039
R.279 Spleen MLR+ vs MLR-	B2M	0.049	0.000	0.33	1	0.997
R.279 Spleen MLR+ vs MLR-	CENPU	0.050	0.000	0.25	0.189	0.038
R.279 Spleen MLR+ vs MLR-	CDT1	0.050	0.000	0.32	0.297	0.083
R.279 Spleen MLR+ vs MLR-	ENSMMUG00000063583	0.065	0.000	0.67	0.946	0.651
R.279 Spleen MLR+ vs MLR-	ENSMMUG00000053403	0.065	0.000	0.28	0.162	0.03
R.279 Spleen MLR+ vs MLR-	NCAPG	0.069	0.000	0.38	0.243	0.059
R.279 Spleen MLR+ vs MLR-	RPL28	0.078	0.000	-0.53	1	0.996
R.279 Spleen MLR+ vs MLR-	ENSMMUG00000004441	0.084	0.000	0.63	1	0.882
R.279 Spleen MLR+ vs MLR-	RPL24	0.093	0.000	-0.48	1	0.991
R.279 Spleen MLR+ vs MLR-	CX3CR1	0.095	0.000	0.51	0.243	0.062
R.279 Spleen MLR+ vs MLR-	GNG5	0.097	0.000	0.55	0.865	0.597
R.279 Spleen MLR+ vs MLR-	ACD	0.099	0.000	0.27	0.27	0.074
R.279 Spleen MLR+ vs MLR-	GPR183	0.137	0.000	-1.10	0.081	0.467
R.279 Spleen MLR+ vs MLR-	DHRS7	0.144	0.000	0.48	0.541	0.239
R.279 Spleen MLR+ vs MLR-	SMC6	0.154	0.000	0.25	0.324	0.1
R.279 Spleen MLR+ vs MLR-	RPS4X	0.157	0.000	-0.42	1	0.996
R.279 Spleen MLR+ vs MLR-	AUH	0.158	0.000	0.26	0.324	0.1
R.279 Spleen MLR+ vs MLR-	CKS1B	0.168	0.000	0.43	0.324	0.104
R.279 Spleen MLR+ vs MLR-	RPS24	0.231	0.000	-0.44	1	0.991
R.279 Spleen MLR+ vs MLR-	KLRD1	0.255	0.000	0.58	0.811	0.449
R.279 Spleen MLR+ vs MLR-	RPL10A	0.262	0.000	-0.56	1	0.992
R.279 Spleen MLR+ vs MLR-	RPLP1	0.291	0.000	-0.46	1	0.994
R.279 Spleen MLR+ vs MLR-	OSR2	0.332	0.000	0.40	0.27	0.08
R.279 Spleen MLR+ vs MLR-	ENSMMUG00000063637	0.492	0.000	-0.46	1	0.994
R.279 Spleen MLR+ vs MLR-	RPL13	0.494	0.000	-0.39	1	0.997
R.279 Spleen MLR+ vs MLR-	EMP3	0.496	0.000	0.62	0.919	0.758
R.279 Spleen MLR+ vs MLR-	RPS16	0.517	0.000	-0.43	1	0.993
R.279 Spleen MLR+ vs MLR-	CMA1	0.544	0.000	0.40	0.324	0.111
R.279 Spleen MLR+ vs MLR-	CDCA8	0.578	0.000	0.28	0.243	0.069
R.279 Spleen MLR+ vs MLR-	RPS8	0.590	0.000	-0.45	1	0.997
R.279 Spleen MLR+ vs MLR-	EEF1A1	0.659	0.000	-0.41	1	0.996
R.279 Spleen MLR+ vs MLR-	NUSAP1	0.854	0.000	0.28	0.243	0.069
R.279 Spleen MLR+ vs MLR-	RPS12	0.993	0.000	-0.38	1	0.998

R.279 Spleen MLR+ vs MLR-	RPS14	1.000	0.000	-0.40	1	0.992
R.279 Spleen MLR+ vs MLR-	CDK1	1.000	0.000	0.50	0.243	0.077
R.279 Spleen MLR+ vs MLR-	ZBTB38	1.000	0.000	0.27	0.459	0.192
R.279 Spleen MLR+ vs MLR-	PRKAA1	1.000	0.000	0.29	0.324	0.116
R.279 Spleen MLR+ vs MLR-	IDH2	1.000	0.000	0.45	0.811	0.498
R.279 Spleen MLR+ vs MLR-	MID1IP1	1.000	0.000	0.29	0.27	0.088
R.279 Spleen MLR+ vs MLR-	SMC2	1.000	0.000	0.38	0.297	0.104
R.279 Spleen MLR+ vs MLR-	RPS17	1.000	0.000	-0.48	0.838	0.94
R.279 Spleen MLR+ vs MLR-	RACK1	1.000	0.000	-0.42	1	0.992
R.279 Spleen MLR+ vs MLR-	SPC25	1.000	0.000	0.34	0.189	0.05
R.279 Spleen MLR+ vs MLR-	RHOH	1.000	0.000	-0.57	0.135	0.452
R.279 Spleen MLR+ vs MLR-	ARRB2	1.000	0.000	0.44	0.676	0.418
R.279 Spleen MLR+ vs MLR-	RPS3	1.000	0.000	-0.42	1	0.992
R.279 Spleen MLR+ vs MLR-	PLAC8	1.000	0.000	-0.88	0.324	0.627
R.279 Spleen MLR+ vs MLR-	ENSMMUG00000061750	1.000	0.000	0.31	0.243	0.076
R.279 Spleen MLR+ vs MLR-	RPL4	1.000	0.000	-0.46	0.973	0.981
R.279 Spleen MLR+ vs MLR-	SLC11A1	1.000	0.000	0.33	0.378	0.157
R.279 Spleen MLR+ vs MLR-	RPL37A	1.000	0.000	-0.37	1	0.997
R.279 Spleen MLR+ vs MLR-	RPL22	1.000	0.000	-0.40	1	0.989
R.279 Spleen MLR+ vs MLR-	HNRNPA2B1	1.000	0.000	0.50	0.919	0.896
R.279 Spleen MLR+ vs MLR-	CLIC1	1.000	0.000	0.58	0.892	0.702
R.279 Spleen MLR+ vs MLR-	ENSMMUG00000014786	1.000	0.000	-0.38	1	0.992
R.279 Spleen MLR+ vs MLR-	RPS25	1.000	0.000	-0.40	1	0.99
R.279 Spleen MLR+ vs MLR-	SMC4	1.000	0.000	0.48	0.405	0.181
R.279 Spleen MLR+ vs MLR-	RPA2	1.000	0.000	0.31	0.351	0.142
R.279 Spleen MLR+ vs MLR-	CPD	1.000	0.000	0.47	0.622	0.341
R.279 Spleen MLR+ vs MLR-	GCN1	1.000	0.000	-0.43	1	0.995
R.279 Spleen MLR+ vs MLR-	RPL6	1.000	0.000	-0.37	1	0.997
R.279 Spleen MLR+ vs MLR-	ENSMMUG00000063609	1.000	0.000	0.43	0.865	0.719
R.279 Spleen MLR+ vs MLR-	HOPX	1.000	0.000	0.44	0.757	0.444
R.279 Spleen MLR+ vs MLR-	GSTK1	1.000	0.000	0.37	0.649	0.347
R.279 Spleen MLR+ vs MLR-	TYMS	1.000	0.000	0.46	0.243	0.08
R.279 Spleen MLR+ vs MLR-	EED	1.000	0.000	0.37	0.541	0.271
R.279 Spleen MLR+ vs MLR-	RPL30	1.000	0.000	-0.39	1	0.994
R.279 Spleen MLR+ vs MLR-	SERPINA1	1.000	0.000	0.57	0.541	0.283
R.279 Spleen MLR+ vs MLR-	CCNA2	1.000	0.000	0.31	0.243	0.079
R.279 Spleen MLR+ vs MLR-	RPS29	1.000	0.000	-0.32	1	0.993
R.279 Spleen MLR+ vs MLR-	TRIM25	1.000	0.000	0.35	0.432	0.197
R.279 Spleen MLR+ vs MLR-	ENSMMUG00000003867	1.000	0.000	-0.37	1	0.997
R.279 Spleen MLR+ vs MLR-	CRIP1	1.000	0.000	0.54	0.973	0.911
R.279 Spleen MLR+ vs MLR-	SLC25A6	1.000	0.000	-0.46	0.838	0.942
R.279 Spleen MLR+ vs MLR-	RPL8	1.000	0.000	-0.37	0.973	0.998
R.279 Spleen MLR+ vs MLR-	AHNAK	1.000	0.000	0.68	0.73	0.489
R.279 Spleen MLR+ vs MLR-	FGFBP2	1.000	0.000	0.27	0.27	0.096
R.279 Spleen MLR+ vs MLR-	GPS2	1.000	0.000	0.29	0.459	0.218
R.279 Spleen MLR+ vs MLR-	RPS7	1.000	0.000	-0.34	1	0.994
R.279 Spleen MLR+ vs MLR-	ENSMMUG00000056183	1.000	0.000	0.26	0.189	0.056
R.279 Spleen MLR+ vs MLR-	LARP7	1.000	0.000	0.30	0.378	0.166
R.279 Spleen MLR+ vs MLR-	RPL27	1.000	0.000	-0.36	1	0.992
R.279 Spleen MLR+ vs MLR-	CBFB	1.000	0.000	0.27	0.351	0.147
R.279 Spleen MLR+ vs MLR-	RPL23A	1.000	0.000	-0.44	0.892	0.923
R.279 Spleen MLR+ vs MLR-	ENSMMUG00000003130	1.000	0.000	0.26	0.514	0.251

R.279 Spleen MLR+ vs MLR-	PRF1	1.000	0.001	0.37	0.649	0.359
R.279 Spleen MLR+ vs MLR-	LRRC8C	1.000	0.001	0.36	0.486	0.248
R.279 Spleen MLR+ vs MLR-	RPL32	1.000	0.001	-0.34	1	0.996
R.279 Spleen MLR+ vs MLR-	LTB	1.000	0.001	-0.75	0.378	0.643
R.279 Spleen MLR+ vs MLR-	RPL10	1.000	0.001	-0.30	1	0.997
R.279 Spleen MLR+ vs MLR-	RPL7A	1.000	0.001	-0.36	1	0.994
R.279 Spleen MLR+ vs MLR-	RPS26	1.000	0.001	-0.31	1	0.995
R.279 Spleen MLR+ vs MLR-	RRM2	1.000	0.001	1.06	0.27	0.107
R.279 Spleen MLR+ vs MLR-	EIF3K	1.000	0.001	-0.41	0.811	0.944
R.279 Spleen MLR+ vs MLR-	SSBP4	1.000	0.001	0.35	0.622	0.362
R.279 Spleen MLR+ vs MLR-	ETS2	1.000	0.001	0.40	0.351	0.153
R.279 Spleen MLR+ vs MLR-	CCL4L1	1.000	0.001	0.63	0.703	0.417
R.279 Spleen MLR+ vs MLR-	PPP1CC	1.000	0.001	-0.61	0.459	0.65
R.279 Spleen MLR+ vs MLR-	RPS9	1.000	0.001	-0.31	1	0.997
R.279 Spleen MLR+ vs MLR-	ISG20	1.000	0.001	0.35	0.892	0.641
R.279 Spleen MLR+ vs MLR-	LASP1	1.000	0.001	0.38	0.514	0.317
R.279 Spleen MLR+ vs MLR-	PLP2	1.000	0.001	0.47	0.703	0.479
R.279 Spleen MLR+ vs MLR-	RAP1GDS1	1.000	0.001	0.29	0.378	0.173
R.279 Spleen MLR+ vs MLR-	ENSMMUG00000062350	1.000	0.001	-0.28	1	0.997
R.279 Spleen MLR+ vs MLR-	ID3	1.000	0.001	-0.78	0.054	0.297
R.279 Spleen MLR+ vs MLR-	HMGB2	1.000	0.001	0.99	0.676	0.486
R.279 Spleen MLR+ vs MLR-	RPL9	1.000	0.001	-0.34	1	0.993
R.279 Spleen MLR+ vs MLR-	RPS5	1.000	0.001	-0.33	1	0.995
R.279 Spleen MLR+ vs MLR-	ENSMMUG00000005593	1.000	0.001	-0.29	1	0.996
R.279 Spleen MLR+ vs MLR-	RPL12	1.000	0.001	-0.39	1	0.995
R.279 Spleen MLR+ vs MLR-	ENSMMUG00000021023	1.000	0.001	0.33	0.189	0.06
R.279 Spleen MLR+ vs MLR-	PRR13	1.000	0.001	0.36	0.946	0.698
R.279 Spleen MLR+ vs MLR-	MKI67	1.000	0.001	0.64	0.405	0.195
R.279 Spleen MLR+ vs MLR-	TRAPPC4	1.000	0.001	0.31	0.514	0.274
R.279 Spleen MLR+ vs MLR-	NACA	1.000	0.001	-0.37	1	0.99
R.279 Spleen MLR+ vs MLR-	KIF20B	1.000	0.001	0.31	0.297	0.123
R.279 Spleen MLR+ vs MLR-	GLIPR2	1.000	0.001	0.42	0.73	0.494
R.279 Spleen MLR+ vs MLR-	H1-5	1.000	0.001	0.35	0.162	0.049
R.279 Spleen MLR+ vs MLR-	ND6	1.000	0.001	-0.45	0.054	0.29
R.279 Spleen MLR+ vs MLR-	GPX4	1.000	0.001	0.47	0.811	0.658
R.279 Spleen MLR+ vs MLR-	CDKN1A	1.000	0.002	0.48	0.514	0.298
R.279 Spleen MLR+ vs MLR-	RAB27A	1.000	0.002	0.26	0.432	0.217
R.279 Spleen MLR+ vs MLR-	FLNA	1.000	0.002	0.58	0.73	0.546
R.279 Spleen MLR+ vs MLR-	RPL18	1.000	0.002	-0.29	1	0.994
R.279 Spleen MLR+ vs MLR-	RPL13A	1.000	0.002	-0.30	1	0.995
R.279 Spleen MLR+ vs MLR-	IFI27L2	1.000	0.002	0.37	0.919	0.845
R.279 Spleen MLR+ vs MLR-	ST3GAL1	1.000	0.002	0.38	0.405	0.212
R.279 Spleen MLR+ vs MLR-	PTTG1	1.000	0.002	0.48	0.405	0.2
R.279 Spleen MLR+ vs MLR-	UBTF	1.000	0.002	0.27	0.351	0.166
R.279 Spleen MLR+ vs MLR-	RBBP8	1.000	0.002	0.26	0.243	0.094
R.279 Spleen MLR+ vs MLR-	RPL15	1.000	0.002	-0.33	1	0.982
R.279 Spleen MLR+ vs MLR-	AP3B1	1.000	0.002	0.36	0.378	0.202
R.279 Spleen MLR+ vs MLR-	H1-4	1.000	0.002	0.57	0.459	0.262
R.279 Spleen MLR+ vs MLR-	MAMU-E	1.000	0.002	0.33	1	0.992
R.279 Spleen MLR+ vs MLR-	CD99	1.000	0.002	0.47	0.703	0.506
R.279 Spleen MLR+ vs MLR-	ENSMMUG00000016898	1.000	0.002	0.62	0.649	0.413
R.279 Spleen MLR+ vs MLR-	CLTA	1.000	0.003	0.42	0.622	0.436

R.279 Spleen MLR+ vs MLR-	RPS28	1.000	0.003	-0.30	1	0.993
R.279 Spleen MLR+ vs MLR-	ENSMMUG00000058581	1.000	0.003	0.62	0.892	0.742
R.279 Spleen MLR+ vs MLR-	TUBA1A	1.000	0.003	0.76	0.811	0.805
R.279 Spleen MLR+ vs MLR-	MAMU-DRA	1.000	0.003	0.47	0.243	0.101
R.279 Spleen MLR+ vs MLR-	CENPW	1.000	0.003	0.29	0.216	0.082
R.279 Spleen MLR+ vs MLR-	RPL17	1.000	0.003	-0.35	1	0.988
R.279 Spleen MLR+ vs MLR-	RPL3	1.000	0.003	-0.28	1	0.996
R.279 Spleen MLR+ vs MLR-	TRAF3IP3	1.000	0.003	0.30	0.676	0.423
R.279 Spleen MLR+ vs MLR-	HELZ	1.000	0.003	0.29	0.351	0.172
R.279 Spleen MLR+ vs MLR-	RGS2	1.000	0.003	-0.59	0.027	0.234
R.279 Spleen MLR+ vs MLR-	NUDT3	1.000	0.003	-0.27	1	0.994
R.279 Spleen MLR+ vs MLR-	KIFC1	1.000	0.003	0.26	0.297	0.131
R.279 Spleen MLR+ vs MLR-	LEF1	1.000	0.003	-0.43	0.027	0.232
R.279 Spleen MLR+ vs MLR-	CLEC2D	1.000	0.003	0.32	0.838	0.673
R.279 Spleen MLR+ vs MLR-	RPL27A	1.000	0.003	-0.38	0.946	0.952
R.279 Spleen MLR+ vs MLR-	ENSMMUG00000022075	1.000	0.003	-0.37	0.297	0.555
R.279 Spleen MLR+ vs MLR-	CEP57	1.000	0.003	0.28	0.324	0.154
R.279 Spleen MLR+ vs MLR-	FOS	1.000	0.004	-0.77	0.703	0.84
R.279 Spleen MLR+ vs MLR-	NME1	1.000	0.004	-0.39	0.838	0.956
R.279 Spleen MLR+ vs MLR-	RPS21	1.000	0.004	-0.26	1	0.995
R.279 Spleen MLR+ vs MLR-	CSTB	1.000	0.004	0.28	0.541	0.321
R.279 Spleen MLR+ vs MLR-	RPL5	1.000	0.004	-0.30	1	0.992
R.279 Spleen MLR+ vs MLR-	TPST2	1.000	0.004	0.32	0.541	0.335
R.279 Spleen MLR+ vs MLR-	CAPN2	1.000	0.004	0.26	0.432	0.235
R.279 Spleen MLR+ vs MLR-	JPT1	1.000	0.004	0.37	0.946	0.87
R.279 Spleen MLR+ vs MLR-	CCR7	1.000	0.004	-0.59	0.081	0.299
R.279 Spleen MLR+ vs MLR-	USP36	1.000	0.004	-0.34	0.054	0.261
R.279 Spleen MLR+ vs MLR-	APMAP	1.000	0.004	0.26	0.351	0.177
R.279 Spleen MLR+ vs MLR-	IPO7	1.000	0.004	0.26	0.378	0.198
R.279 Spleen MLR+ vs MLR-	SRI	1.000	0.004	0.32	0.73	0.501
R.279 Spleen MLR+ vs MLR-	CTLA4	1.000	0.005	-0.42	0	0.181
R.279 Spleen MLR+ vs MLR-	G3BP2	1.000	0.005	-0.50	0.378	0.582
R.279 Spleen MLR+ vs MLR-	HPCAL1	1.000	0.005	-0.32	0.081	0.291
R.279 Spleen MLR+ vs MLR-	TMED10	1.000	0.005	-0.37	0.189	0.405
R.279 Spleen MLR+ vs MLR-	KLF6	1.000	0.005	-0.44	0.622	0.851
R.279 Spleen MLR+ vs MLR-	NFKB1	1.000	0.005	-0.40	0.135	0.346
R.279 Spleen MLR+ vs MLR-	EPC1	1.000	0.005	-0.29	0.054	0.264
R.279 Spleen MLR+ vs MLR-	PCLAF	1.000	0.005	0.36	0.216	0.086
R.279 Spleen MLR+ vs MLR-	MAPK1	1.000	0.006	0.26	0.459	0.26
R.279 Spleen MLR+ vs MLR-	SELL	1.000	0.006	-0.41	0.135	0.373
R.279 Spleen MLR+ vs MLR-	PPIA	1.000	0.006	0.33	1	0.99
R.279 Spleen MLR+ vs MLR-	ENSMMUG00000017890	1.000	0.006	-0.35	0.162	0.371
R.279 Spleen MLR+ vs MLR-	MTPN	1.000	0.006	0.31	0.595	0.395
R.279 Spleen MLR+ vs MLR-	RAP1B	1.000	0.007	0.39	0.811	0.768
R.279 Spleen MLR+ vs MLR-	RNASEH2A	1.000	0.007	0.26	0.216	0.093
R.279 Spleen MLR+ vs MLR-	TGFBR3	1.000	0.007	0.32	0.486	0.288
R.279 Spleen MLR+ vs MLR-	ENSMMUG00000013429	1.000	0.007	-0.40	1	0.996
R.279 Spleen MLR+ vs MLR-	RPA3	1.000	0.007	0.39	0.703	0.523
R.279 Spleen MLR+ vs MLR-	PRPF6	1.000	0.007	0.27	0.459	0.269
R.279 Spleen MLR+ vs MLR-	MT2A	1.000	0.008	0.44	0.595	0.411
R.279 Spleen MLR+ vs MLR-	SOCS3	1.000	0.008	-0.30	0.081	0.284
R.279 Spleen MLR+ vs MLR-	TRAPPC3	1.000	0.008	0.26	0.432	0.246

R.279 Spleen MLR+ vs MLR-	MLF2	1.000	0.008	-0.32	0.162	0.365
R.279 Spleen MLR+ vs MLR-	RPS11	1.000	0.008	-0.30	1	0.995
R.279 Spleen MLR+ vs MLR-	COX7A1	1.000	0.008	-0.25	0	0.16
R.279 Spleen MLR+ vs MLR-	ANXA2	1.000	0.008	0.49	0.676	0.516
R.279 Spleen MLR+ vs MLR-	DNAJC1	1.000	0.008	0.28	0.486	0.295
R.279 Spleen MLR+ vs MLR-	ENSMMUG00000004633	1.000	0.008	0.26	0.243	0.112
R.279 Spleen MLR+ vs MLR-	IFI6	1.000	0.008	0.26	0.784	0.619
R.279 Spleen MLR+ vs MLR-	SH3KBP1	1.000	0.009	0.39	0.703	0.57
R.279 Spleen MLR+ vs MLR-	CMTR1	1.000	0.009	0.27	0.324	0.166
R.279 Spleen MLR+ vs MLR-	FLOT1	1.000	0.009	0.44	0.541	0.36
R.279 Spleen MLR+ vs MLR-	RNF166	1.000	0.009	0.27	0.297	0.154
R.279 Spleen MLR+ vs MLR-	DNMT1	1.000	0.009	0.43	0.405	0.247
R.279 Spleen MLR+ vs MLR-	CSRNP1	1.000	0.010	-0.44	0.405	0.581
R.279 Spleen MLR+ vs MLR-	HSPA8	1.000	0.010	0.50	0.973	0.98
R.279 Spleen MLR+ vs MLR-	GTF2I	1.000	0.010	-0.29	0.081	0.267
R.279 Spleen MLR+ vs MLR-	PLCG1	1.000	0.010	-0.26	0.027	0.195
R.279 Spleen MLR+ vs MLR-	CD38	1.000	0.010	-0.45	0.27	0.45
R.279 Spleen MLR+ vs MLR-	SSU72	1.000	0.010	-0.30	0.108	0.299
R.279 Spleen MLR+ vs MLR-	EIF4EBP2	1.000	0.010	0.29	0.351	0.191
R.279 Spleen MLR+ vs MLR-	FBL	1.000	0.010	-0.33	0.189	0.39
R.279 Spleen MLR+ vs MLR-	ARPC5L	1.000	0.010	0.31	0.757	0.561
R.279 Spleen MLR+ vs MLR-	AIMP1	1.000	0.011	-0.33	0.162	0.354
R.279 Spleen MLR+ vs MLR-	INPP4B	1.000	0.011	-0.34	0.135	0.33
R.279 Spleen MLR+ vs MLR-	ENSMMUG00000057791	1.000	0.011	-0.50	0	0.15
R.279 Spleen MLR+ vs MLR-	MAMU-F	1.000	0.011	0.28	0.432	0.265
R.279 Spleen MLR+ vs MLR-	MCM7	1.000	0.011	0.31	0.243	0.116
R.279 Spleen MLR+ vs MLR-	RABAC1	1.000	0.011	0.38	0.595	0.425
R.279 Spleen MLR+ vs MLR-	SUB1	1.000	0.011	0.30	0.865	0.702
R.279 Spleen MLR+ vs MLR-	CA6	1.000	0.011	-0.41	0.027	0.189
R.279 Spleen MLR+ vs MLR-	CALR	1.000	0.012	-0.41	0.622	0.804
R.279 Spleen MLR+ vs MLR-	TCF7	1.000	0.012	-0.37	0.162	0.354
R.279 Spleen MLR+ vs MLR-	SYTL2	1.000	0.013	0.37	0.378	0.228
R.279 Spleen MLR+ vs MLR-	ADSL	1.000	0.013	-0.30	0.081	0.258
R.279 Spleen MLR+ vs MLR-	SDHAF2	1.000	0.013	-0.27	0.081	0.258
R.279 Spleen MLR+ vs MLR-	DGKA	1.000	0.013	-0.30	0.108	0.296
R.279 Spleen MLR+ vs MLR-	WBP11	1.000	0.013	-0.29	0.108	0.289
R.279 Spleen MLR+ vs MLR-	SLC2A3	1.000	0.014	-0.57	0.405	0.559
R.279 Spleen MLR+ vs MLR-	LCP1	1.000	0.014	0.28	0.838	0.767
R.279 Spleen MLR+ vs MLR-	LAG3	1.000	0.014	-0.50	0.081	0.255
R.279 Spleen MLR+ vs MLR-	SH2D2A	1.000	0.014	0.32	0.514	0.35
R.279 Spleen MLR+ vs MLR-	CKS2	1.000	0.014	0.29	0.541	0.354
R.279 Spleen MLR+ vs MLR-	RBM39	1.000	0.014	0.30	0.919	0.775
R.279 Spleen MLR+ vs MLR-	ALDOA	1.000	0.015	-0.38	0.784	0.836
R.279 Spleen MLR+ vs MLR-	CASP3	1.000	0.015	-0.30	0.135	0.322
R.279 Spleen MLR+ vs MLR-	SKAP1	1.000	0.015	0.26	0.865	0.651
R.279 Spleen MLR+ vs MLR-	PSMC5	1.000	0.015	-0.28	0.189	0.383
R.279 Spleen MLR+ vs MLR-	APOBEC3G	1.000	0.016	0.30	0.541	0.362
R.279 Spleen MLR+ vs MLR-	RRM1	1.000	0.016	0.29	0.351	0.201
R.279 Spleen MLR+ vs MLR-	DUSP2	1.000	0.016	-0.63	0.378	0.53
R.279 Spleen MLR+ vs MLR-	MRPL23	1.000	0.016	-0.33	0.351	0.551
R.279 Spleen MLR+ vs MLR-	ICOS	1.000	0.016	-0.43	0.135	0.31
R.279 Spleen MLR+ vs MLR-	MBNL1	1.000	0.016	-0.32	0.595	0.762

R.279 Spleen MLR+ vs MLR-	ATP1A1	1.000	0.017	0.30	0.486	0.326
R.279 Spleen MLR+ vs MLR-	SFRP5	1.000	0.017	-0.30	0	0.134
R.279 Spleen MLR+ vs MLR-	CCNI	1.000	0.017	-0.35	0.378	0.552
R.279 Spleen MLR+ vs MLR-	SERPINB1	1.000	0.017	0.38	0.486	0.343
R.279 Spleen MLR+ vs MLR-	ENSMMUG00000002320	1.000	0.017	-0.36	0.946	0.986
R.279 Spleen MLR+ vs MLR-	ENSMMUG00000008604	1.000	0.018	-0.26	0.054	0.213
R.279 Spleen MLR+ vs MLR-	ENSMMUG00000006283	1.000	0.018	-0.34	0.838	0.81
R.279 Spleen MLR+ vs MLR-	CD164	1.000	0.018	-0.32	0.432	0.608
R.279 Spleen MLR+ vs MLR-	FOSB	1.000	0.018	-0.54	0.622	0.772
R.279 Spleen MLR+ vs MLR-	CYLD	1.000	0.018	-0.27	0.189	0.386
R.279 Spleen MLR+ vs MLR-	UBE2F	1.000	0.018	-0.27	0.054	0.212
R.279 Spleen MLR+ vs MLR-	YWHAB	1.000	0.018	0.28	0.892	0.749
R.279 Spleen MLR+ vs MLR-	PIM2	1.000	0.018	-0.27	0.054	0.211
R.279 Spleen MLR+ vs MLR-	LENG8	1.000	0.018	-0.26	0.081	0.247
R.279 Spleen MLR+ vs MLR-	CENPM	1.000	0.019	0.31	0.216	0.099
R.279 Spleen MLR+ vs MLR-	RCSD1	1.000	0.019	-0.29	0.162	0.345
R.279 Spleen MLR+ vs MLR-	GALNS	1.000	0.019	-0.35	0.378	0.55
R.279 Spleen MLR+ vs MLR-	STK10	1.000	0.019	0.25	0.514	0.339
R.279 Spleen MLR+ vs MLR-	ENSMMUG00000012140	1.000	0.020	0.30	1	0.997
R.279 Spleen MLR+ vs MLR-	COMMD6	1.000	0.020	-0.33	0.568	0.733
R.279 Spleen MLR+ vs MLR-	CASP4	1.000	0.021	-0.25	0.081	0.242
R.279 Spleen MLR+ vs MLR-	STING1	1.000	0.021	-0.27	0.054	0.206
R.279 Spleen MLR+ vs MLR-	ENSMMUG00000003412	1.000	0.021	-0.26	1	0.995
R.279 Spleen MLR+ vs MLR-	PRRC2C	1.000	0.021	-0.32	0.486	0.64
R.279 Spleen MLR+ vs MLR-	CEMIP2	1.000	0.021	-0.25	0.162	0.35
R.279 Spleen MLR+ vs MLR-	ORAI1	1.000	0.022	0.29	0.622	0.465
R.279 Spleen MLR+ vs MLR-	CD74	1.000	0.022	0.52	0.703	0.602
R.279 Spleen MLR+ vs MLR-	USP1	1.000	0.022	0.28	0.378	0.229
R.279 Spleen MLR+ vs MLR-	PELI1	1.000	0.022	-0.38	0.216	0.375
R.279 Spleen MLR+ vs MLR-	CD28	1.000	0.022	-0.28	0.054	0.204
R.279 Spleen MLR+ vs MLR-	PM20D2	1.000	0.022	-0.29	0.676	0.847
R.279 Spleen MLR+ vs MLR-	BIRC5	1.000	0.022	0.26	0.162	0.07
R.279 Spleen MLR+ vs MLR-	DGKZ	1.000	0.022	0.26	0.568	0.399
R.279 Spleen MLR+ vs MLR-	IL10RA	1.000	0.022	0.30	0.568	0.398
R.279 Spleen MLR+ vs MLR-	ENSMMUG00000056515	1.000	0.023	-0.83	0	0.124
R.279 Spleen MLR+ vs MLR-	TOP2A	1.000	0.023	0.28	0.189	0.084
R.279 Spleen MLR+ vs MLR-	TUBB	1.000	0.023	0.78	0.622	0.521
R.279 Spleen MLR+ vs MLR-	EEF1G	1.000	0.023	-0.31	0.865	0.894
R.279 Spleen MLR+ vs MLR-	RAB1B	1.000	0.023	0.34	0.595	0.441
R.279 Spleen MLR+ vs MLR-	ENSMMUG00000051392	1.000	0.023	0.58	0.595	0.519
R.279 Spleen MLR+ vs MLR-	PHIP	1.000	0.023	0.29	0.405	0.267
R.279 Spleen MLR+ vs MLR-	HBP1	1.000	0.023	-0.26	0.135	0.31
R.279 Spleen MLR+ vs MLR-	JUNB	1.000	0.024	-0.42	0.405	0.595
R.279 Spleen MLR+ vs MLR-	ENSMMUG00000065238	1.000	0.024	0.55	0.216	0.107
R.279 Spleen MLR+ vs MLR-	TBC1D10B	1.000	0.024	-0.35	0.541	0.646
R.279 Spleen MLR+ vs MLR-	BHLHE40	1.000	0.024	0.33	0.784	0.598
R.279 Spleen MLR+ vs MLR-	NDFIP1	1.000	0.025	-0.32	0.405	0.574
R.279 Spleen MLR+ vs MLR-	CUTA	1.000	0.025	-0.32	0.351	0.572
R.279 Spleen MLR+ vs MLR-	FOSL2	1.000	0.025	-0.31	0.135	0.295
R.279 Spleen MLR+ vs MLR-	BIRC2	1.000	0.025	-0.30	0.189	0.354
R.279 Spleen MLR+ vs MLR-	CD83	1.000	0.026	-0.61	0.324	0.479
R.279 Spleen MLR+ vs MLR-	FTH1	1.000	0.027	-0.37	0.838	0.918

R.279 Spleen MLR+ vs MLR-	APOL2	1.000	0.027	0.26	0.622	0.428
R.279 Spleen MLR+ vs MLR-	UBE2B	1.000	0.028	0.30	0.622	0.475
R.279 Spleen MLR+ vs MLR-	KLF3	1.000	0.028	0.28	0.378	0.241
R.279 Spleen MLR+ vs MLR-	SPCS2	1.000	0.028	0.30	0.541	0.376
R.279 Spleen MLR+ vs MLR-	KIFBP	1.000	0.029	-0.25	0.162	0.347
R.279 Spleen MLR+ vs MLR-	EGLN3	1.000	0.029	-0.27	0.054	0.195
R.279 Spleen MLR+ vs MLR-	ALYREF	1.000	0.029	0.36	0.541	0.386
R.279 Spleen MLR+ vs MLR-	DEK	1.000	0.030	0.37	0.622	0.47
R.279 Spleen MLR+ vs MLR-	NR4A2	1.000	0.030	-0.51	0.703	0.774
R.279 Spleen MLR+ vs MLR-	H2AFX	1.000	0.030	0.53	0.486	0.336
R.279 Spleen MLR+ vs MLR-	SPN	1.000	0.030	0.28	0.514	0.351
R.279 Spleen MLR+ vs MLR-	ENSMMUG00000002324	1.000	0.031	-0.28	0.189	0.347
R.279 Spleen MLR+ vs MLR-	ETS1	1.000	0.031	-0.30	0.649	0.77
R.279 Spleen MLR+ vs MLR-	ENSMMUG00000064232	1.000	0.032	-0.27	0.162	0.325
R.279 Spleen MLR+ vs MLR-	STMN1	1.000	0.033	1.16	0.459	0.348
R.279 Spleen MLR+ vs MLR-	LGALS3	1.000	0.033	0.26	0.703	0.499
R.279 Spleen MLR+ vs MLR-	ATF2	1.000	0.034	-0.27	0.135	0.287
R.279 Spleen MLR+ vs MLR-	ENSMMUG00000060689	1.000	0.034	0.29	0.108	0.039
R.279 Spleen MLR+ vs MLR-	REL	1.000	0.034	-0.35	0.378	0.529
R.279 Spleen MLR+ vs MLR-	CD3G	1.000	0.036	0.29	0.811	0.769
R.279 Spleen MLR+ vs MLR-	UCP2	1.000	0.037	0.46	0.757	0.694
R.279 Spleen MLR+ vs MLR-	HSD17B11	1.000	0.037	0.31	0.405	0.269
R.279 Spleen MLR+ vs MLR-	ATP5F1C	1.000	0.038	-0.29	0.351	0.496
R.279 Spleen MLR+ vs MLR-	GPRIN3	1.000	0.038	-0.25	0.108	0.252
R.279 Spleen MLR+ vs MLR-	SRSF5	1.000	0.038	-0.27	0.595	0.722
R.279 Spleen MLR+ vs MLR-	ID2	1.000	0.039	-0.48	0.514	0.65
R.279 Spleen MLR+ vs MLR-	B3GNT2	1.000	0.039	-0.30	0.216	0.362
R.279 Spleen MLR+ vs MLR-	MVP	1.000	0.040	0.27	0.541	0.385
R.279 Spleen MLR+ vs MLR-	ARF4	1.000	0.041	-0.27	0.459	0.607
R.279 Spleen MLR+ vs MLR-	VIM	1.000	0.041	0.37	0.946	0.91
R.279 Spleen MLR+ vs MLR-	RPL31	1.000	0.041	-0.32	0.514	0.604
R.279 Spleen MLR+ vs MLR-	TALDO1	1.000	0.042	-0.27	0.324	0.478
R.279 Spleen MLR+ vs MLR-	ATP5MJ	1.000	0.042	0.25	0.973	0.843
R.279 Spleen MLR+ vs MLR-	MAGEH1	1.000	0.043	-0.33	0.108	0.245
R.279 Spleen MLR+ vs MLR-	RBM38	1.000	0.043	-0.38	0.459	0.571
R.279 Spleen MLR+ vs MLR-	ZFP36	1.000	0.043	-0.40	0.865	0.913
R.279 Spleen MLR+ vs MLR-	PECAM1	1.000	0.044	-0.25	0.135	0.283
R.279 Spleen MLR+ vs MLR-	RNF167	1.000	0.044	0.29	0.973	0.811
R.279 Spleen MLR+ vs MLR-	RGS16	1.000	0.045	0.47	0.243	0.143
R.279 Spleen MLR+ vs MLR-	HIST1H2AE	1.000	0.046	0.36	0.216	0.112
R.279 Spleen MLR+ vs MLR-	CCDC82	1.000	0.046	0.32	0.405	0.278
R.279 Spleen MLR+ vs MLR-	MIF	1.000	0.046	-0.33	0.595	0.735
R.279 Spleen MLR+ vs MLR-	EIF3E	1.000	0.046	-0.29	0.649	0.715
R.279 Spleen MLR+ vs MLR-	PIM3	1.000	0.046	-0.30	0.108	0.242
R.279 Spleen MLR+ vs MLR-	MGST3	1.000	0.047	0.26	0.459	0.334
R.279 Spleen MLR+ vs MLR-	TAF15	1.000	0.049	0.35	0.568	0.418
R.279 Spleen MLR+ vs MLR-	TKT	1.000	0.052	-0.25	0.243	0.39
R.279 Spleen MLR+ vs MLR-	ENSMMUG00000006499	1.000	0.053	-0.27	0.216	0.354
R.279 Spleen MLR+ vs MLR-	ENSMMUG00000017466	1.000	0.053	0.35	0.784	0.684
R.279 Spleen MLR+ vs MLR-	GNB2	1.000	0.054	0.27	0.676	0.582
R.279 Spleen MLR+ vs MLR-	HNRNPAB	1.000	0.054	0.38	0.541	0.444
R.279 Spleen MLR+ vs MLR-	RPS27L	1.000	0.054	0.47	0.595	0.557

R.279 Spleen MLR+ vs MLR-	CNN2	1.000	0.054	-0.30	0.514	0.613
R.279 Spleen MLR+ vs MLR-	LSM4	1.000	0.054	-0.26	0.27	0.42
R.279 Spleen MLR+ vs MLR-	ACAP1	1.000	0.054	-0.27	0.324	0.458
R.279 Spleen MLR+ vs MLR-	ATF3	1.000	0.055	0.30	0.405	0.264
R.279 Spleen MLR+ vs MLR-	TOMM20	1.000	0.056	-0.32	0.541	0.634
R.279 Spleen MLR+ vs MLR-	ENSMMUG00000050862	1.000	0.058	-0.64	0.081	0.199
R.279 Spleen MLR+ vs MLR-	TAF7	1.000	0.059	0.26	0.568	0.437
R.279 Spleen MLR+ vs MLR-	LMNB1	1.000	0.059	0.26	0.297	0.186
R.279 Spleen MLR+ vs MLR-	AIP	1.000	0.061	-0.25	0.351	0.492
R.279 Spleen MLR+ vs MLR-	TMEM123	1.000	0.065	-0.28	0.324	0.461
R.279 Spleen MLR+ vs MLR-	HNRNPDL	1.000	0.065	-0.26	0.838	0.855
R.279 Spleen MLR+ vs MLR-	TMEM258	1.000	0.068	0.30	0.784	0.681
R.279 Spleen MLR+ vs MLR-	NR4A3	1.000	0.068	-0.30	0.162	0.284
R.279 Spleen MLR+ vs MLR-	STX11	1.000	0.072	0.27	0.459	0.336
R.279 Spleen MLR+ vs MLR-	ARHGAP9	1.000	0.073	-0.28	0.297	0.426
R.279 Spleen MLR+ vs MLR-	ENSMMUG00000061128	1.000	0.073	0.33	0.622	0.504
R.279 Spleen MLR+ vs MLR-	TIGAR	1.000	0.075	-0.26	0.189	0.327
R.279 Spleen MLR+ vs MLR-	CYTIP	1.000	0.077	0.27	0.622	0.534
R.279 Spleen MLR+ vs MLR-	SLC9A3R1	1.000	0.081	0.26	0.622	0.481
R.279 Spleen MLR+ vs MLR-	PPDPF	1.000	0.086	0.31	0.622	0.5
R.279 Spleen MLR+ vs MLR-	LPXN	1.000	0.087	-0.25	0.27	0.383
R.279 Spleen MLR+ vs MLR-	SAP18	1.000	0.087	0.26	0.703	0.618
R.279 Spleen MLR+ vs MLR-	HOOK3	1.000	0.090	0.26	0.459	0.364
R.279 Spleen MLR+ vs MLR-	PSMB10	1.000	0.092	-0.26	0.351	0.462
R.279 Spleen MLR+ vs MLR-	CXCR4	1.000	0.092	0.34	0.838	0.726
R.279 Spleen MLR+ vs MLR-	NMT1	1.000	0.094	0.33	0.27	0.184
R.279 Spleen MLR+ vs MLR-	RNASEH2B	1.000	0.096	0.29	0.324	0.234
R.279 Spleen MLR+ vs MLR-	PCNA	1.000	0.103	0.30	0.459	0.335
R.279 Spleen MLR+ vs MLR-	TYROBP	1.000	0.106	-0.31	0.054	0.15
R.279 Spleen MLR+ vs MLR-	C11H12orf57	1.000	0.106	-0.26	0.757	0.785
R.279 Spleen MLR+ vs MLR-	LIMD2	1.000	0.106	-0.27	0.595	0.648
R.279 Spleen MLR+ vs MLR-	CRTAM	1.000	0.106	-0.41	0.216	0.317
R.279 Spleen MLR+ vs MLR-	DNAJB1	1.000	0.108	0.32	0.459	0.339
R.279 Spleen MLR+ vs MLR-	H2AFZ	1.000	0.109	0.43	0.703	0.633
R.279 Spleen MLR+ vs MLR-	HMGB1	1.000	0.110	0.51	0.865	0.78
R.279 Spleen MLR+ vs MLR-	ND4L	1.000	0.113	-0.49	0.027	0.11
R.279 Spleen MLR+ vs MLR-	ENSMMUG00000063316	1.000	0.114	-0.29	0.054	0.142
R.279 Spleen MLR+ vs MLR-	COX2	1.000	0.116	-0.46	0.892	0.95
R.279 Spleen MLR+ vs MLR-	EIF1AX	1.000	0.120	-0.25	0.351	0.454
R.279 Spleen MLR+ vs MLR-	GPCPD1	1.000	0.121	-0.29	0.378	0.463
R.279 Spleen MLR+ vs MLR-	JUN	1.000	0.126	-0.30	0.73	0.825
R.279 Spleen MLR+ vs MLR-	HNRNPA3	1.000	0.141	0.39	0.649	0.576
R.279 Spleen MLR+ vs MLR-	XCL1	1.000	0.153	-0.55	0.054	0.133
R.279 Spleen MLR+ vs MLR-	TMPO	1.000	0.153	0.31	0.351	0.283
R.279 Spleen MLR+ vs MLR-	NR4A1	1.000	0.161	-0.25	0.243	0.351
R.279 Spleen MLR+ vs MLR-	ENSMMUG00000060751	1.000	0.163	0.29	0.162	0.098
R.279 Spleen MLR+ vs MLR-	GSTP1	1.000	0.163	-0.27	0.784	0.878
R.279 Spleen MLR+ vs MLR-	SELPLG	1.000	0.169	0.31	0.459	0.38
R.279 Spleen MLR+ vs MLR-	HSP90AA1	1.000	0.171	0.28	0.892	0.901
R.279 Spleen MLR+ vs MLR-	YPEL5	1.000	0.171	-0.27	0.622	0.651
R.279 Spleen MLR+ vs MLR-	H1-3	1.000	0.182	0.44	0.189	0.123
R.279 Spleen MLR+ vs MLR-	LUC7L3	1.000	0.198	0.27	0.27	0.194

R.279 Spleen MLR+ vs MLR-	ZFP36L1	1.000	0.199	-0.30	0.541	0.611
R.279 Spleen MLR+ vs MLR-	S100A6	1.000	0.203	-0.29	0.595	0.691
R.279 Spleen MLR+ vs MLR-	CCL3	1.000	0.203	-0.27	0.081	0.152
R.279 Spleen MLR+ vs MLR-	IL2RB	1.000	0.227	-0.27	0.405	0.48
R.279 Spleen MLR+ vs MLR-	ENSMMUG00000028672	1.000	0.240	-0.28	0.568	0.616
R.279 Spleen MLR+ vs MLR-	BCL2A1	1.000	0.321	0.26	0.649	0.649
R.279 Spleen MLR+ vs MLR-	RGS1	1.000	0.330	-0.27	0.405	0.457
R.279 Spleen MLR+ vs MLR-	TSC22D3	1.000	0.333	0.26	0.865	0.818
R.279 Spleen MLR+ vs MLR-	IFI27	1.000	0.354	0.25	0.622	0.563
R.279 Spleen MLR+ vs MLR-	CHORDC1	1.000	0.359	0.30	0.324	0.271
R.279 Spleen MLR+ vs MLR-	ENSMMUG00000055690	1.000	0.363	-0.38	1	0.998
R.279 Spleen MLR+ vs MLR-	MYH9	1.000	0.369	0.37	0.73	0.705
R.279 Spleen MLR+ vs MLR-	RGCC	1.000	0.390	-0.29	0.73	0.789
R.279 Spleen MLR+ vs MLR-	TXNIP	1.000	0.398	0.31	0.432	0.426
R.279 Spleen MLR+ vs MLR-	ANP32B	1.000	0.416	0.31	0.568	0.583
R.279 Spleen MLR+ vs MLR-	LMNA	1.000	0.447	-0.33	0.351	0.385
R.279 Spleen MLR+ vs MLR-	GZMA	1.000	0.502	-0.67	0.405	0.314
R.279 Spleen MLR+ vs MLR-	TUBA4A	1.000	0.505	-0.34	0.703	0.624
R.279 Spleen MLR+ vs MLR-	CD69	1.000	0.789	0.30	0.73	0.764
R.279 Spleen MLR+ vs MLR-	ENSMMUG00000062894	1.000	0.844	-0.28	0.73	0.701
R.279 Spleen MLR+ vs MLR-	ENSMMUG00000062077	1.000	0.868	-1.00	1	0.998
R.319 Spleen MLR+ vs MLR-	ENSMMUG00000020332	0.000	0.000	1.37	0.231	0.008
R.319 Spleen MLR+ vs MLR-	KIR3DL12	0.000	0.000	0.38	0.282	0.015
R.319 Spleen MLR+ vs MLR-	ENSMMUG00000050862	0.000	0.000	1.70	0.641	0.099
R.319 Spleen MLR+ vs MLR-	TYROBP	0.000	0.000	1.48	0.59	0.104
R.319 Spleen MLR+ vs MLR-	CCL5	0.000	0.000	2.06	0.769	0.187
R.319 Spleen MLR+ vs MLR-	NKG7	0.000	0.000	1.91	0.897	0.314
R.319 Spleen MLR+ vs MLR-	VEGFA	0.000	0.000	0.35	0.231	0.021
R.319 Spleen MLR+ vs MLR-	ITGAX	0.000	0.000	0.78	0.538	0.106
R.319 Spleen MLR+ vs MLR-	CST7	0.000	0.000	1.36	0.872	0.331
R.319 Spleen MLR+ vs MLR-	ENSMMUG00000056183	0.000	0.000	0.44	0.333	0.047
R.319 Spleen MLR+ vs MLR-	ZEB2	0.000	0.000	0.59	0.436	0.08
R.319 Spleen MLR+ vs MLR-	ENSMMUG00000063583	0.000	0.000	1.46	0.846	0.31
R.319 Spleen MLR+ vs MLR-	NCR1	0.000	0.000	0.37	0.231	0.025
R.319 Spleen MLR+ vs MLR-	GZMB	0.000	0.000	1.27	0.795	0.244
R.319 Spleen MLR+ vs MLR-	CTSW	0.000	0.000	1.29	0.667	0.222
R.319 Spleen MLR+ vs MLR-	MAMU-DRB1	0.000	0.000	0.71	0.41	0.085
R.319 Spleen MLR+ vs MLR-	CCL4L1	0.000	0.000	1.00	0.615	0.178
R.319 Spleen MLR+ vs MLR-	KLRD1	0.000	0.000	0.56	0.513	0.123
R.319 Spleen MLR+ vs MLR-	EOMES	0.000	0.000	0.37	0.308	0.053
R.319 Spleen MLR+ vs MLR-	B2M	0.000	0.000	0.46	1	1
R.319 Spleen MLR+ vs MLR-	RPL8	0.000	0.000	-0.59	1	1
R.319 Spleen MLR+ vs MLR-	HCST	0.000	0.000	0.95	0.974	0.662
R.319 Spleen MLR+ vs MLR-	ENSMMUG00000064139	0.000	0.000	0.67	1	0.998
R.319 Spleen MLR+ vs MLR-	RPS8	0.000	0.000	-0.61	1	1
R.319 Spleen MLR+ vs MLR-	GZMM	0.000	0.000	1.18	0.564	0.198
R.319 Spleen MLR+ vs MLR-	ENSMMUG00000058581	0.000	0.000	1.07	0.667	0.269
R.319 Spleen MLR+ vs MLR-	EEF1A1	0.000	0.000	-0.54	1	1
R.319 Spleen MLR+ vs MLR-	ENSMMUG00000003532	0.000	0.000	0.62	0.641	0.211
R.319 Spleen MLR+ vs MLR-	HOPX	0.000	0.000	0.65	0.667	0.23
R.319 Spleen MLR+ vs MLR-	ALOX5AP	0.000	0.000	0.52	0.487	0.144
R.319 Spleen MLR+ vs MLR-	MAMU-A	0.000	0.000	0.43	1	1

R.319 Spleen MLR+ vs MLR-	MYRF	0.000	0.000	0.35	0.231	0.039
R.319 Spleen MLR+ vs MLR-	RPL28	0.000	0.000	-0.61	1	1
R.319 Spleen MLR+ vs MLR-	LTB	0.000	0.000	-1.22	0.513	0.842
R.319 Spleen MLR+ vs MLR-	RPS4X	0.000	0.000	-0.55	1	1
R.319 Spleen MLR+ vs MLR-	ADAMTS7	0.000	0.000	0.27	0.231	0.041
R.319 Spleen MLR+ vs MLR-	CD160	0.000	0.000	0.44	0.205	0.034
R.319 Spleen MLR+ vs MLR-	RPL10	0.000	0.000	-0.50	1	1
R.319 Spleen MLR+ vs MLR-	GZMK	0.000	0.000	0.54	0.436	0.125
R.319 Spleen MLR+ vs MLR-	PLEK	0.000	0.000	0.35	0.256	0.052
R.319 Spleen MLR+ vs MLR-	ENSMMUG00000005593	0.000	0.000	-0.54	1	1
R.319 Spleen MLR+ vs MLR-	SPOCK2	0.000	0.000	-0.90	0.256	0.734
R.319 Spleen MLR+ vs MLR-	RACK1	0.001	0.000	-0.57	1	1
R.319 Spleen MLR+ vs MLR-	FGR	0.001	0.000	0.34	0.205	0.037
R.319 Spleen MLR+ vs MLR-	ENSMMUG00000064120	0.001	0.000	0.53	1	0.946
R.319 Spleen MLR+ vs MLR-	SCML4	0.002	0.000	0.67	0.59	0.257
R.319 Spleen MLR+ vs MLR-	ETS2	0.002	0.000	0.43	0.41	0.128
R.319 Spleen MLR+ vs MLR-	ZC3H10	0.003	0.000	-0.62	0.897	0.979
R.319 Spleen MLR+ vs MLR-	GCN1	0.003	0.000	-0.48	1	1
R.319 Spleen MLR+ vs MLR-	RPS3A	0.003	0.000	-0.50	1	1
R.319 Spleen MLR+ vs MLR-	RGS9	0.003	0.000	0.58	0.359	0.111
R.319 Spleen MLR+ vs MLR-	RPL22L1	0.003	0.000	-0.91	0.436	0.789
R.319 Spleen MLR+ vs MLR-	RPL7A	0.005	0.000	-0.49	1	1
R.319 Spleen MLR+ vs MLR-	RPL12	0.006	0.000	-0.56	1	1
R.319 Spleen MLR+ vs MLR-	ENSMMUG00000052609	0.006	0.000	-0.48	1	1
R.319 Spleen MLR+ vs MLR-	EEF1G	0.007	0.000	-0.69	0.846	0.937
R.319 Spleen MLR+ vs MLR-	CCR7	0.008	0.000	-0.95	0.103	0.544
R.319 Spleen MLR+ vs MLR-	ENSMMUG00000014786	0.008	0.000	-0.47	1	1
R.319 Spleen MLR+ vs MLR-	SKAP2	0.008	0.000	0.39	0.308	0.084
R.319 Spleen MLR+ vs MLR-	KLRG1	0.010	0.000	0.32	0.179	0.033
R.319 Spleen MLR+ vs MLR-	HBA	0.011	0.000	0.36	0.308	0.086
R.319 Spleen MLR+ vs MLR-	RPL30	0.012	0.000	-0.44	1	1
R.319 Spleen MLR+ vs MLR-	RPL5	0.015	0.000	-0.47	1	1
R.319 Spleen MLR+ vs MLR-	S100A10	0.015	0.000	0.68	0.949	0.909
R.319 Spleen MLR+ vs MLR-	RPS23	0.015	0.000	-0.43	1	1
R.319 Spleen MLR+ vs MLR-	MAMU-E	0.016	0.000	0.43	1	0.999
R.319 Spleen MLR+ vs MLR-	RPS7	0.018	0.000	-0.39	1	1
R.319 Spleen MLR+ vs MLR-	CD7	0.023	0.000	-0.85	0.308	0.679
R.319 Spleen MLR+ vs MLR-	ENSMMUG00000013429	0.028	0.000	-0.48	1	1
R.319 Spleen MLR+ vs MLR-	HARS2	0.031	0.000	0.26	0.256	0.066
R.319 Spleen MLR+ vs MLR-	ELOA	0.035	0.000	-0.40	1	1
R.319 Spleen MLR+ vs MLR-	RPL18	0.037	0.000	-0.41	1	1
R.319 Spleen MLR+ vs MLR-	RPS16	0.039	0.000	-0.41	1	1
R.319 Spleen MLR+ vs MLR-	CD63	0.047	0.000	0.70	0.615	0.33
R.319 Spleen MLR+ vs MLR-	EFHD2	0.047	0.000	0.59	0.718	0.403
R.319 Spleen MLR+ vs MLR-	RPL37A	0.053	0.000	-0.45	1	0.998
R.319 Spleen MLR+ vs MLR-	RPS26	0.056	0.000	-0.35	1	1
R.319 Spleen MLR+ vs MLR-	CD74	0.056	0.000	0.67	0.641	0.322
R.319 Spleen MLR+ vs MLR-	ENSMMUG00000003867	0.057	0.000	-0.44	1	1
R.319 Spleen MLR+ vs MLR-	ENSMMUG00000062350	0.063	0.000	-0.38	1	1
R.319 Spleen MLR+ vs MLR-	CPD	0.066	0.000	0.40	0.436	0.168
R.319 Spleen MLR+ vs MLR-	RPS24	0.072	0.000	-0.45	1	0.999
R.319 Spleen MLR+ vs MLR-	RPS13	0.073	0.000	-0.38	1	1

R.319 Spleen MLR+ vs MLR-	PPP1CC	0.078	0.000	-0.79	0.538	0.756
R.319 Spleen MLR+ vs MLR-	RPSA	0.138	0.000	-0.39	1	1
R.319 Spleen MLR+ vs MLR-	GPR183	0.153	0.000	-0.88	0.282	0.646
R.319 Spleen MLR+ vs MLR-	RPS5	0.160	0.000	-0.37	1	1
R.319 Spleen MLR+ vs MLR-	NACA	0.161	0.000	-0.44	1	0.999
R.319 Spleen MLR+ vs MLR-	TPT1	0.176	0.000	-0.51	1	1
R.319 Spleen MLR+ vs MLR-	ENSMMUG00000063637	0.203	0.000	-0.41	1	1
R.319 Spleen MLR+ vs MLR-	RPLP1	0.241	0.000	-0.32	1	1
R.319 Spleen MLR+ vs MLR-	RPS12	0.286	0.000	-0.37	1	1
R.319 Spleen MLR+ vs MLR-	RPS21	0.317	0.000	-0.39	1	1
R.319 Spleen MLR+ vs MLR-	S100A4	0.345	0.000	0.57	0.974	0.695
R.319 Spleen MLR+ vs MLR-	RPS28	0.374	0.000	-0.32	1	1
R.319 Spleen MLR+ vs MLR-	LEF1	0.432	0.000	-0.64	0.077	0.406
R.319 Spleen MLR+ vs MLR-	DDAH2	0.513	0.000	0.27	0.179	0.044
R.319 Spleen MLR+ vs MLR-	ENSMMUG00000054038	0.518	0.000	0.41	1	0.988
R.319 Spleen MLR+ vs MLR-	CD84	0.639	0.000	0.31	0.359	0.136
R.319 Spleen MLR+ vs MLR-	NKG2D	0.722	0.000	0.35	0.333	0.119
R.319 Spleen MLR+ vs MLR-	CASP12	0.838	0.000	-0.61	0.462	0.738
R.319 Spleen MLR+ vs MLR-	SERPINA1	0.857	0.000	0.42	0.59	0.287
R.319 Spleen MLR+ vs MLR-	RPL32	1.000	0.000	-0.35	1	1
R.319 Spleen MLR+ vs MLR-	COX2	1.000	0.000	-0.45	1	1
R.319 Spleen MLR+ vs MLR-	THY1	1.000	0.000	0.51	0.462	0.209
R.319 Spleen MLR+ vs MLR-	RNF103	1.000	0.000	0.29	0.333	0.126
R.319 Spleen MLR+ vs MLR-	COX1	1.000	0.000	-0.52	0.949	0.979
R.319 Spleen MLR+ vs MLR-	RPL10A	1.000	0.000	-0.43	1	0.999
R.319 Spleen MLR+ vs MLR-	CMC1	1.000	0.000	0.49	0.436	0.204
R.319 Spleen MLR+ vs MLR-	RPL23A	1.000	0.000	-0.51	0.667	0.88
R.319 Spleen MLR+ vs MLR-	CXCR4	1.000	0.000	0.68	0.846	0.643
R.319 Spleen MLR+ vs MLR-	RPAP3	1.000	0.000	0.27	0.205	0.06
R.319 Spleen MLR+ vs MLR-	MAP4K1	1.000	0.000	0.40	0.59	0.322
R.319 Spleen MLR+ vs MLR-	DDX5	1.000	0.000	0.37	0.949	0.958
R.319 Spleen MLR+ vs MLR-	RPS27A.1	1.000	0.000	-0.35	1	1
R.319 Spleen MLR+ vs MLR-	CRTAM	1.000	0.000	0.36	0.282	0.103
R.319 Spleen MLR+ vs MLR-	RPL6	1.000	0.000	-0.31	1	1
R.319 Spleen MLR+ vs MLR-	PPP1R12A	1.000	0.000	0.31	0.59	0.312
R.319 Spleen MLR+ vs MLR-	RPL24	1.000	0.000	-0.33	1	1
R.319 Spleen MLR+ vs MLR-	RPL22	1.000	0.000	-0.35	1	0.999
R.319 Spleen MLR+ vs MLR-	DHRS7	1.000	0.000	0.37	0.487	0.24
R.319 Spleen MLR+ vs MLR-	CD28	1.000	0.000	-0.47	0.077	0.349
R.319 Spleen MLR+ vs MLR-	BST2	1.000	0.000	0.52	0.821	0.614
R.319 Spleen MLR+ vs MLR-	UBB	1.000	0.000	0.42	0.949	0.932
R.319 Spleen MLR+ vs MLR-	ITGB2	1.000	0.000	0.49	0.795	0.637
R.319 Spleen MLR+ vs MLR-	RSRP1	1.000	0.000	0.49	0.641	0.382
R.319 Spleen MLR+ vs MLR-	CAPG	1.000	0.000	-0.67	0.513	0.708
R.319 Spleen MLR+ vs MLR-	ENSMMUG00000004441	1.000	0.000	0.42	0.974	0.93
R.319 Spleen MLR+ vs MLR-	DGKA	1.000	0.001	-0.46	0.179	0.463
R.319 Spleen MLR+ vs MLR-	RPS3	1.000	0.001	-0.32	1	0.998
R.319 Spleen MLR+ vs MLR-	NUDT3	1.000	0.001	-0.29	1	1
R.319 Spleen MLR+ vs MLR-	NMI	1.000	0.001	0.34	0.41	0.195
R.319 Spleen MLR+ vs MLR-	SLC2A3	1.000	0.001	-0.65	0.359	0.621
R.319 Spleen MLR+ vs MLR-	RPS15	1.000	0.001	-0.28	1	1
R.319 Spleen MLR+ vs MLR-	SSBP4	1.000	0.001	0.45	0.487	0.274

R.319 Spleen MLR+ vs MLR-	PRF1	1.000	0.001	0.38	0.41	0.19
R.319 Spleen MLR+ vs MLR-	PXN	1.000	0.001	0.33	0.385	0.182
R.319 Spleen MLR+ vs MLR-	FCMR	1.000	0.001	-0.44	0.103	0.373
R.319 Spleen MLR+ vs MLR-	RPL13A	1.000	0.001	-0.29	1	1
R.319 Spleen MLR+ vs MLR-	RPL38	1.000	0.001	-0.30	1	1
R.319 Spleen MLR+ vs MLR-	NBEAL2	1.000	0.001	0.30	0.308	0.128
R.319 Spleen MLR+ vs MLR-	ICOS	1.000	0.001	-0.52	0.154	0.414
R.319 Spleen MLR+ vs MLR-	RPS14	1.000	0.001	-0.30	1	1
R.319 Spleen MLR+ vs MLR-	LAMP1	1.000	0.001	0.40	0.641	0.421
R.319 Spleen MLR+ vs MLR-	RPL35A	1.000	0.001	-0.27	1	1
R.319 Spleen MLR+ vs MLR-	SERTAD2	1.000	0.001	-0.34	0	0.224
R.319 Spleen MLR+ vs MLR-	ST8SIA4	1.000	0.001	0.29	0.436	0.215
R.319 Spleen MLR+ vs MLR-	ANP32B	1.000	0.001	-0.45	0.333	0.608
R.319 Spleen MLR+ vs MLR-	TIGIT	1.000	0.001	0.28	0.513	0.268
R.319 Spleen MLR+ vs MLR-	PECAM1	1.000	0.001	-0.41	0.051	0.292
R.319 Spleen MLR+ vs MLR-	DGKZ	1.000	0.001	0.43	0.59	0.385
R.319 Spleen MLR+ vs MLR-	HNRNPA1	1.000	0.001	-0.37	0.974	0.963
R.319 Spleen MLR+ vs MLR-	RGCC	1.000	0.001	-0.61	0.718	0.873
R.319 Spleen MLR+ vs MLR-	EEF2	1.000	0.001	-0.35	0.974	0.997
R.319 Spleen MLR+ vs MLR-	ATP6AP2	1.000	0.001	0.36	0.462	0.253
R.319 Spleen MLR+ vs MLR-	JUNB	1.000	0.001	-0.57	0.487	0.695
R.319 Spleen MLR+ vs MLR-	ELF4	1.000	0.001	0.25	0.359	0.166
R.319 Spleen MLR+ vs MLR-	CFP	1.000	0.001	-0.32	0.051	0.305
R.319 Spleen MLR+ vs MLR-	CLEC2D	1.000	0.001	0.36	0.795	0.592
R.319 Spleen MLR+ vs MLR-	STK17A	1.000	0.001	-0.44	0.718	0.832
R.319 Spleen MLR+ vs MLR-	ENSMMUG00000013256	1.000	0.001	-0.38	0.41	0.69
R.319 Spleen MLR+ vs MLR-	ENSMMUG00000002320	1.000	0.001	0.43	1	0.997
R.319 Spleen MLR+ vs MLR-	COTL1	1.000	0.001	-0.57	0.564	0.808
R.319 Spleen MLR+ vs MLR-	ZBTB38	1.000	0.001	0.25	0.282	0.119
R.319 Spleen MLR+ vs MLR-	LAPTM5	1.000	0.001	-0.47	0.923	0.949
R.319 Spleen MLR+ vs MLR-	RPL13	1.000	0.002	-0.27	1	1
R.319 Spleen MLR+ vs MLR-	DCTN3	1.000	0.002	-0.38	0.154	0.404
R.319 Spleen MLR+ vs MLR-	RPL3	1.000	0.002	-0.29	0.974	1
R.319 Spleen MLR+ vs MLR-	UBA52	1.000	0.002	-0.26	1	1
R.319 Spleen MLR+ vs MLR-	INPP5D	1.000	0.002	0.26	0.359	0.17
R.319 Spleen MLR+ vs MLR-	CTSL	1.000	0.002	-0.41	0.051	0.28
R.319 Spleen MLR+ vs MLR-	ENSMMUG00000022489	1.000	0.002	-0.35	0.974	0.942
R.319 Spleen MLR+ vs MLR-	RPL4	1.000	0.002	-0.32	1	0.988
R.319 Spleen MLR+ vs MLR-	TRIB2	1.000	0.002	-0.34	0.026	0.236
R.319 Spleen MLR+ vs MLR-	PLAC8	1.000	0.002	-0.78	0.282	0.524
R.319 Spleen MLR+ vs MLR-	H2AFZ	1.000	0.002	-0.59	0.462	0.69
R.319 Spleen MLR+ vs MLR-	FYB1	1.000	0.002	-0.41	0.462	0.741
R.319 Spleen MLR+ vs MLR-	ITGA1	1.000	0.002	-0.33	0	0.197
R.319 Spleen MLR+ vs MLR-	CSNK2B	1.000	0.002	-0.36	0.282	0.545
R.319 Spleen MLR+ vs MLR-	CTSC	1.000	0.002	0.27	0.436	0.228
R.319 Spleen MLR+ vs MLR-	EIF2S2	1.000	0.002	-0.31	0.103	0.36
R.319 Spleen MLR+ vs MLR-	PTPRC	1.000	0.002	0.35	0.974	0.94
R.319 Spleen MLR+ vs MLR-	TSC22D3	1.000	0.002	0.43	0.974	0.875
R.319 Spleen MLR+ vs MLR-	IRF1	1.000	0.002	0.31	0.974	0.858
R.319 Spleen MLR+ vs MLR-	REL	1.000	0.003	-0.40	0.205	0.446
R.319 Spleen MLR+ vs MLR-	PDE4B	1.000	0.003	-0.42	0.154	0.384
R.319 Spleen MLR+ vs MLR-	ATP5MC2	1.000	0.003	-0.40	0.846	0.885

R.319 Spleen MLR+ vs MLR-	CHD3	1.000	0.003	-0.36	0.154	0.398
R.319 Spleen MLR+ vs MLR-	HNRNPA2B1	1.000	0.003	0.27	0.974	0.894
R.319 Spleen MLR+ vs MLR-	CNBP	1.000	0.003	0.36	0.821	0.745
R.319 Spleen MLR+ vs MLR-	ATP5F1D	1.000	0.003	-0.39	0.615	0.808
R.319 Spleen MLR+ vs MLR-	FRG1	1.000	0.003	-0.31	0.051	0.261
R.319 Spleen MLR+ vs MLR-	DNAJA1	1.000	0.003	0.52	0.744	0.549
R.319 Spleen MLR+ vs MLR-	CLK1	1.000	0.003	0.32	0.538	0.332
R.319 Spleen MLR+ vs MLR-	RPL9	1.000	0.003	-0.29	0.974	0.999
R.319 Spleen MLR+ vs MLR-	ABRACL	1.000	0.003	-0.40	0.385	0.66
R.319 Spleen MLR+ vs MLR-	SOCS3	1.000	0.003	-0.40	0.179	0.406
R.319 Spleen MLR+ vs MLR-	SMCHD1	1.000	0.003	-0.34	0.128	0.365
R.319 Spleen MLR+ vs MLR-	TCF7	1.000	0.003	-0.49	0.308	0.526
R.319 Spleen MLR+ vs MLR-	ENSMMUG00000064692	1.000	0.003	-0.38	0.513	0.744
R.319 Spleen MLR+ vs MLR-	EIF3B	1.000	0.004	-0.30	0.051	0.252
R.319 Spleen MLR+ vs MLR-	RPL39	1.000	0.004	-0.25	1	1
R.319 Spleen MLR+ vs MLR-	ARHGAP9	1.000	0.004	0.41	0.538	0.35
R.319 Spleen MLR+ vs MLR-	SELL	1.000	0.004	-0.58	0.256	0.484
R.319 Spleen MLR+ vs MLR-	RPS6KA1	1.000	0.004	0.33	0.41	0.227
R.319 Spleen MLR+ vs MLR-	EEF1B2	1.000	0.005	-0.35	0.949	0.983
R.319 Spleen MLR+ vs MLR-	EIF3E	1.000	0.005	-0.39	0.513	0.712
R.319 Spleen MLR+ vs MLR-	CD38	1.000	0.005	-0.48	0.154	0.353
R.319 Spleen MLR+ vs MLR-	VAMP5	1.000	0.005	-0.32	0.103	0.307
R.319 Spleen MLR+ vs MLR-	FKBP8	1.000	0.005	0.28	0.667	0.459
R.319 Spleen MLR+ vs MLR-	DECR2	1.000	0.005	-0.26	0.128	0.369
R.319 Spleen MLR+ vs MLR-	PGD	1.000	0.005	-0.27	0.026	0.209
R.319 Spleen MLR+ vs MLR-	CD5	1.000	0.005	-0.37	0.179	0.403
R.319 Spleen MLR+ vs MLR-	IL2RB	1.000	0.005	0.50	0.59	0.393
R.319 Spleen MLR+ vs MLR-	ZFP36	1.000	0.005	-0.45	0.872	0.938
R.319 Spleen MLR+ vs MLR-	HSD17B4	1.000	0.005	-0.32	0.103	0.314
R.319 Spleen MLR+ vs MLR-	RPL23	1.000	0.005	-0.33	0.872	0.918
R.319 Spleen MLR+ vs MLR-	SLC25A6	1.000	0.005	-0.32	0.897	0.977
R.319 Spleen MLR+ vs MLR-	ENSMMUG00000017097	1.000	0.005	0.28	0.41	0.231
R.319 Spleen MLR+ vs MLR-	CDKN1A	1.000	0.006	-0.55	0.128	0.327
R.319 Spleen MLR+ vs MLR-	SFRP5	1.000	0.006	-0.34	0	0.166
R.319 Spleen MLR+ vs MLR-	ENSMMUG00000020050	1.000	0.006	-0.29	0.179	0.395
R.319 Spleen MLR+ vs MLR-	PPM1J	1.000	0.006	0.34	0.308	0.155
R.319 Spleen MLR+ vs MLR-	FNTB	1.000	0.006	-0.32	0.179	0.403
R.319 Spleen MLR+ vs MLR-	CYB5A	1.000	0.006	-0.33	0.154	0.374
R.319 Spleen MLR+ vs MLR-	COX5A	1.000	0.007	-0.37	0.487	0.661
R.319 Spleen MLR+ vs MLR-	RABAC1	1.000	0.007	0.33	0.615	0.424
R.319 Spleen MLR+ vs MLR-	ITGB7	1.000	0.007	-0.30	0.256	0.503
R.319 Spleen MLR+ vs MLR-	RPL7	1.000	0.007	-0.29	0.897	0.953
R.319 Spleen MLR+ vs MLR-	HSPA8	1.000	0.007	0.28	1	0.955
R.319 Spleen MLR+ vs MLR-	MRPS34	1.000	0.007	-0.30	0.154	0.363
R.319 Spleen MLR+ vs MLR-	ENSMMUG00000064873	1.000	0.007	-0.29	0.179	0.39
R.319 Spleen MLR+ vs MLR-	CD3G	1.000	0.007	0.33	0.897	0.798
R.319 Spleen MLR+ vs MLR-	CLIC1	1.000	0.007	0.31	0.872	0.745
R.319 Spleen MLR+ vs MLR-	PLK2	1.000	0.007	-0.31	0	0.157
R.319 Spleen MLR+ vs MLR-	FAM102A	1.000	0.007	-0.26	0.026	0.196
R.319 Spleen MLR+ vs MLR-	SLC25A43	1.000	0.008	-0.34	0.385	0.67
R.319 Spleen MLR+ vs MLR-	FLOT1	1.000	0.008	-0.34	0.205	0.42
R.319 Spleen MLR+ vs MLR-	PAG1	1.000	0.008	-0.30	0.077	0.263

R.319 Spleen MLR+ vs MLR-	UBE2L6	1.000	0.008	-0.27	0.385	0.606
R.319 Spleen MLR+ vs MLR-	HNRNPH2	1.000	0.008	-0.29	1	0.985
R.319 Spleen MLR+ vs MLR-	ENSMMUG00000059937	1.000	0.008	-0.38	0.077	0.267
R.319 Spleen MLR+ vs MLR-	STMN1	1.000	0.008	-0.72	0.103	0.297
R.319 Spleen MLR+ vs MLR-	TMEM50A	1.000	0.009	0.32	0.59	0.424
R.319 Spleen MLR+ vs MLR-	EIF3F	1.000	0.009	-0.32	0.615	0.865
R.319 Spleen MLR+ vs MLR-	NDUFAB1	1.000	0.009	-0.29	0.103	0.289
R.319 Spleen MLR+ vs MLR-	LGALS3	1.000	0.009	0.39	0.615	0.437
R.319 Spleen MLR+ vs MLR-	IL4R	1.000	0.009	-0.31	0.103	0.294
R.319 Spleen MLR+ vs MLR-	RNF138	1.000	0.010	-0.26	0.051	0.223
R.319 Spleen MLR+ vs MLR-	LIMD2	1.000	0.010	-0.34	0.487	0.729
R.319 Spleen MLR+ vs MLR-	ATP5PO	1.000	0.011	-0.26	0.744	0.863
R.319 Spleen MLR+ vs MLR-	VIM	1.000	0.011	-0.41	0.974	0.975
R.319 Spleen MLR+ vs MLR-	SPRYD3	1.000	0.012	0.28	0.282	0.147
R.319 Spleen MLR+ vs MLR-	PPP3CC	1.000	0.012	0.30	0.436	0.276
R.319 Spleen MLR+ vs MLR-	R3HDM4	1.000	0.012	-0.27	0.154	0.355
R.319 Spleen MLR+ vs MLR-	RANBP1	1.000	0.012	-0.32	0.128	0.307
R.319 Spleen MLR+ vs MLR-	COPRS	1.000	0.012	-0.32	0.128	0.306
R.319 Spleen MLR+ vs MLR-	PARP8	1.000	0.012	0.31	0.333	0.184
R.319 Spleen MLR+ vs MLR-	NPM1	1.000	0.012	-0.28	0.923	0.975
R.319 Spleen MLR+ vs MLR-	CUTA	1.000	0.013	-0.31	0.179	0.367
R.319 Spleen MLR+ vs MLR-	NFKB2	1.000	0.014	-0.25	0.051	0.212
R.319 Spleen MLR+ vs MLR-	SYAP1	1.000	0.014	-0.28	0.103	0.276
R.319 Spleen MLR+ vs MLR-	NDUFS8	1.000	0.014	-0.30	0.205	0.393
R.319 Spleen MLR+ vs MLR-	EIF3M	1.000	0.014	-0.32	0.359	0.576
R.319 Spleen MLR+ vs MLR-	ORAI1	1.000	0.014	-0.28	0.231	0.428
R.319 Spleen MLR+ vs MLR-	S1PR1	1.000	0.014	-0.28	0.179	0.391
R.319 Spleen MLR+ vs MLR-	HERPUD1	1.000	0.014	0.42	0.641	0.506
R.319 Spleen MLR+ vs MLR-	NUTF2	1.000	0.014	-0.30	0.205	0.383
R.319 Spleen MLR+ vs MLR-	SERTAD1	1.000	0.014	0.40	0.692	0.585
R.319 Spleen MLR+ vs MLR-	DUSP2	1.000	0.014	-0.59	0.513	0.656
R.319 Spleen MLR+ vs MLR-	PNISR	1.000	0.015	0.35	0.513	0.386
R.319 Spleen MLR+ vs MLR-	PBXIP1	1.000	0.015	-0.29	0.256	0.459
R.319 Spleen MLR+ vs MLR-	NUDC	1.000	0.015	0.36	0.564	0.405
R.319 Spleen MLR+ vs MLR-	DRAP1	1.000	0.015	0.35	0.564	0.44
R.319 Spleen MLR+ vs MLR-	PTP4A1	1.000	0.015	-0.31	0.154	0.336
R.319 Spleen MLR+ vs MLR-	LIMS1	1.000	0.015	-0.36	0.231	0.403
R.319 Spleen MLR+ vs MLR-	CYBA	1.000	0.015	0.32	0.821	0.765
R.319 Spleen MLR+ vs MLR-	SLCO3A1	1.000	0.016	0.27	0.179	0.078
R.319 Spleen MLR+ vs MLR-	SERPINB9	1.000	0.016	0.35	0.513	0.343
R.319 Spleen MLR+ vs MLR-	GPSM3	1.000	0.016	-0.34	0.538	0.726
R.319 Spleen MLR+ vs MLR-	HM13	1.000	0.016	-0.30	0.179	0.354
R.319 Spleen MLR+ vs MLR-	TMEM243	1.000	0.016	-0.27	0.103	0.271
R.319 Spleen MLR+ vs MLR-	RPL35	1.000	0.016	-0.26	1	0.987
R.319 Spleen MLR+ vs MLR-	LMNA	1.000	0.016	-0.43	0.282	0.495
R.319 Spleen MLR+ vs MLR-	NUFIP2	1.000	0.017	-0.26	0.103	0.277
R.319 Spleen MLR+ vs MLR-	NDUFV2	1.000	0.017	-0.31	0.256	0.455
R.319 Spleen MLR+ vs MLR-	RHOG	1.000	0.017	-0.33	0.41	0.557
R.319 Spleen MLR+ vs MLR-	RGS10	1.000	0.018	-0.27	0.103	0.267
R.319 Spleen MLR+ vs MLR-	ND4L	1.000	0.018	-0.33	0.846	0.978
R.319 Spleen MLR+ vs MLR-	SELENOK	1.000	0.018	0.25	0.538	0.368
R.319 Spleen MLR+ vs MLR-	GADD45B	1.000	0.018	0.31	0.718	0.583

R.319 Spleen MLR+ vs MLR-	DNAJC19	1.000	0.019	0.26	0.385	0.239
R.319 Spleen MLR+ vs MLR-	CD53	1.000	0.020	0.30	0.769	0.631
R.319 Spleen MLR+ vs MLR-	AHI1	1.000	0.020	-0.29	0.179	0.368
R.319 Spleen MLR+ vs MLR-	INPP4B	1.000	0.021	-0.27	0.154	0.341
R.319 Spleen MLR+ vs MLR-	PABPC1	1.000	0.022	-0.29	0.897	0.955
R.319 Spleen MLR+ vs MLR-	APOBEC3G	1.000	0.022	0.32	0.41	0.266
R.319 Spleen MLR+ vs MLR-	PTPN22	1.000	0.023	0.27	0.333	0.193
R.319 Spleen MLR+ vs MLR-	HINT1	1.000	0.023	-0.26	0.974	0.941
R.319 Spleen MLR+ vs MLR-	CHMP2A	1.000	0.024	-0.25	0.128	0.288
R.319 Spleen MLR+ vs MLR-	GSTP1	1.000	0.024	0.26	0.923	0.885
R.319 Spleen MLR+ vs MLR-	ENSMMUG00000018740	1.000	0.025	0.28	0.641	0.589
R.319 Spleen MLR+ vs MLR-	CD6	1.000	0.025	-0.32	0.282	0.445
R.319 Spleen MLR+ vs MLR-	CD164	1.000	0.025	-0.32	0.462	0.629
R.319 Spleen MLR+ vs MLR-	PRR13	1.000	0.026	0.28	0.744	0.681
R.319 Spleen MLR+ vs MLR-	CD99	1.000	0.027	0.40	0.641	0.537
R.319 Spleen MLR+ vs MLR-	WDR83OS	1.000	0.027	-0.27	0.538	0.763
R.319 Spleen MLR+ vs MLR-	CCT5	1.000	0.027	-0.30	0.333	0.49
R.319 Spleen MLR+ vs MLR-	MT1E	1.000	0.028	-0.42	0.179	0.343
R.319 Spleen MLR+ vs MLR-	IK	1.000	0.028	0.28	0.564	0.44
R.319 Spleen MLR+ vs MLR-	ITGA6	1.000	0.028	-0.25	0.103	0.251
R.319 Spleen MLR+ vs MLR-	CA6	1.000	0.029	-0.36	0.077	0.215
R.319 Spleen MLR+ vs MLR-	ENO1	1.000	0.029	-0.39	0.564	0.758
R.319 Spleen MLR+ vs MLR-	KLF10	1.000	0.029	-0.32	0.154	0.304
R.319 Spleen MLR+ vs MLR-	CTLA4	1.000	0.029	-0.30	0.128	0.284
R.319 Spleen MLR+ vs MLR-	TNFSF12	1.000	0.030	0.26	0.359	0.23
R.319 Spleen MLR+ vs MLR-	ERGIC3	1.000	0.030	-0.27	0.231	0.384
R.319 Spleen MLR+ vs MLR-	NUCB2	1.000	0.030	-0.28	0.231	0.393
R.319 Spleen MLR+ vs MLR-	CAST	1.000	0.031	0.27	0.564	0.391
R.319 Spleen MLR+ vs MLR-	AHNAK	1.000	0.031	0.33	0.667	0.554
R.319 Spleen MLR+ vs MLR-	ENSMMUG00000063316	1.000	0.031	-0.48	0.487	0.637
R.319 Spleen MLR+ vs MLR-	ITGB1	1.000	0.031	-0.49	0.385	0.547
R.319 Spleen MLR+ vs MLR-	EIF3H	1.000	0.032	-0.25	0.667	0.788
R.319 Spleen MLR+ vs MLR-	ALDOA	1.000	0.032	-0.30	0.821	0.925
R.319 Spleen MLR+ vs MLR-	ENSMMUG00000053146	1.000	0.033	-0.26	0.256	0.41
R.319 Spleen MLR+ vs MLR-	ENSMMUG00000055584	1.000	0.034	-0.27	0.462	0.625
R.319 Spleen MLR+ vs MLR-	STX11	1.000	0.034	0.35	0.333	0.207
R.319 Spleen MLR+ vs MLR-	CCL3	1.000	0.034	0.38	0.154	0.069
R.319 Spleen MLR+ vs MLR-	CYLD	1.000	0.034	-0.27	0.231	0.393
R.319 Spleen MLR+ vs MLR-	PLK3	1.000	0.035	-0.30	0.256	0.404
R.319 Spleen MLR+ vs MLR-	ANXA1	1.000	0.036	0.36	0.795	0.681
R.319 Spleen MLR+ vs MLR-	PRDX1	1.000	0.037	-0.34	0.436	0.602
R.319 Spleen MLR+ vs MLR-	ENSMMUG00000043332	1.000	0.037	-0.26	0.692	0.813
R.319 Spleen MLR+ vs MLR-	PSMA6	1.000	0.037	0.30	0.564	0.433
R.319 Spleen MLR+ vs MLR-	C3H7orf50	1.000	0.038	0.36	0.641	0.571
R.319 Spleen MLR+ vs MLR-	NFATC1	1.000	0.039	-0.27	0.128	0.274
R.319 Spleen MLR+ vs MLR-	ENSMMUG00000003854	1.000	0.039	0.27	0.59	0.468
R.319 Spleen MLR+ vs MLR-	SSH2	1.000	0.039	-0.25	0.154	0.298
R.319 Spleen MLR+ vs MLR-	CALR	1.000	0.040	0.29	0.897	0.848
R.319 Spleen MLR+ vs MLR-	TMSB10	1.000	0.040	-0.30	1	1
R.319 Spleen MLR+ vs MLR-	IGFLR1	1.000	0.040	-0.27	0.205	0.354
R.319 Spleen MLR+ vs MLR-	SON	1.000	0.040	0.25	0.821	0.736
R.319 Spleen MLR+ vs MLR-	APBB1IP	1.000	0.041	0.29	0.59	0.47

R.319 Spleen MLR+ vs MLR-	SEPTIN6	1.000	0.042	-0.31	0.436	0.559
R.319 Spleen MLR+ vs MLR-	RHEB	1.000	0.043	-0.27	0.231	0.384
R.319 Spleen MLR+ vs MLR-	PFKL	1.000	0.044	-0.27	0.256	0.4
R.319 Spleen MLR+ vs MLR-	TBC1D10B	1.000	0.045	-0.28	0.564	0.745
R.319 Spleen MLR+ vs MLR-	TNFSF10	1.000	0.046	-0.29	0.103	0.231
R.319 Spleen MLR+ vs MLR-	TAPBPL	1.000	0.048	0.27	0.872	0.775
R.319 Spleen MLR+ vs MLR-	MVP	1.000	0.048	-0.27	0.359	0.5
R.319 Spleen MLR+ vs MLR-	KRTCAP2	1.000	0.049	-0.28	0.692	0.805
R.319 Spleen MLR+ vs MLR-	ARID5B	1.000	0.050	-0.26	0.231	0.378
R.319 Spleen MLR+ vs MLR-	SEPTIN7	1.000	0.051	0.26	0.641	0.554
R.319 Spleen MLR+ vs MLR-	CYCS	1.000	0.051	-0.27	0.359	0.513
R.319 Spleen MLR+ vs MLR-	IKZF3	1.000	0.052	0.29	0.333	0.214
R.319 Spleen MLR+ vs MLR-	MAPRE2	1.000	0.052	0.29	0.538	0.408
R.319 Spleen MLR+ vs MLR-	IL7R	1.000	0.052	-0.36	0.359	0.536
R.319 Spleen MLR+ vs MLR-	IGFBP4	1.000	0.053	-0.28	0.026	0.132
R.319 Spleen MLR+ vs MLR-	RCSD1	1.000	0.053	-0.26	0.308	0.454
R.319 Spleen MLR+ vs MLR-	HNRNPDL	1.000	0.054	0.25	0.923	0.88
R.319 Spleen MLR+ vs MLR-	KLF2	1.000	0.056	-0.32	0.923	0.917
R.319 Spleen MLR+ vs MLR-	SRI	1.000	0.056	0.27	0.59	0.461
R.319 Spleen MLR+ vs MLR-	TIMP1	1.000	0.058	-0.33	0.231	0.371
R.319 Spleen MLR+ vs MLR-	ND5	1.000	0.060	-0.27	0.436	0.553
R.319 Spleen MLR+ vs MLR-	CKS2	1.000	0.062	-0.31	0.231	0.365
R.319 Spleen MLR+ vs MLR-	GTF2B	1.000	0.063	0.29	0.692	0.553
R.319 Spleen MLR+ vs MLR-	MAP4	1.000	0.064	0.27	0.359	0.248
R.319 Spleen MLR+ vs MLR-	SKAP1	1.000	0.067	0.26	0.744	0.68
R.319 Spleen MLR+ vs MLR-	ARL6IP5	1.000	0.067	0.30	0.59	0.525
R.319 Spleen MLR+ vs MLR-	HSP90AA1	1.000	0.068	0.32	0.949	0.923
R.319 Spleen MLR+ vs MLR-	PIM3	1.000	0.071	-0.32	0.256	0.375
R.319 Spleen MLR+ vs MLR-	HSPA5	1.000	0.074	0.30	0.872	0.842
R.319 Spleen MLR+ vs MLR-	CNN2	1.000	0.075	-0.27	0.615	0.719
R.319 Spleen MLR+ vs MLR-	DNAJB1	1.000	0.078	0.49	0.538	0.424
R.319 Spleen MLR+ vs MLR-	COX8A	1.000	0.078	0.28	0.718	0.646
R.319 Spleen MLR+ vs MLR-	GHITM	1.000	0.081	0.25	0.641	0.511
R.319 Spleen MLR+ vs MLR-	RILPL2	1.000	0.082	-0.26	0.231	0.351
R.319 Spleen MLR+ vs MLR-	JAK1	1.000	0.083	0.32	0.692	0.612
R.319 Spleen MLR+ vs MLR-	RBM39	1.000	0.083	0.28	0.821	0.733
R.319 Spleen MLR+ vs MLR-	LRPAP1	1.000	0.087	0.29	0.333	0.226
R.319 Spleen MLR+ vs MLR-	ARRB2	1.000	0.095	0.26	0.462	0.364
R.319 Spleen MLR+ vs MLR-	ARHGAP4	1.000	0.095	0.26	0.513	0.427
R.319 Spleen MLR+ vs MLR-	KIAA0040	1.000	0.096	0.28	0.308	0.213
R.319 Spleen MLR+ vs MLR-	CYFIP2	1.000	0.097	0.30	0.41	0.326
R.319 Spleen MLR+ vs MLR-	WIPF1	1.000	0.097	0.29	0.641	0.595
R.319 Spleen MLR+ vs MLR-	ODC1	1.000	0.097	0.26	0.564	0.473
R.319 Spleen MLR+ vs MLR-	TUBB	1.000	0.097	-0.54	0.513	0.63
R.319 Spleen MLR+ vs MLR-	HMGB1	1.000	0.098	-0.30	0.59	0.756
R.319 Spleen MLR+ vs MLR-	ITM2A	1.000	0.099	-0.29	0.462	0.555
R.319 Spleen MLR+ vs MLR-	COPE	1.000	0.101	0.27	0.564	0.519
R.319 Spleen MLR+ vs MLR-	HSPH1	1.000	0.114	0.41	0.462	0.411
R.319 Spleen MLR+ vs MLR-	GADD45A	1.000	0.119	0.33	0.282	0.197
R.319 Spleen MLR+ vs MLR-	ND4	1.000	0.119	-0.26	0.846	0.878
R.319 Spleen MLR+ vs MLR-	USP47	1.000	0.125	0.29	0.282	0.207
R.319 Spleen MLR+ vs MLR-	TOB1	1.000	0.126	-0.28	0.538	0.63

R.319 Spleen MLR+ vs MLR-	G3BP2	1.000	0.127	-0.33	0.487	0.554
R.319 Spleen MLR+ vs MLR-	TAF7	1.000	0.130	0.27	0.359	0.271
R.319 Spleen MLR+ vs MLR-	RGS1	1.000	0.132	0.26	0.538	0.432
R.319 Spleen MLR+ vs MLR-	ENSMMUG00000055690	1.000	0.133	0.42	1	1
R.319 Spleen MLR+ vs MLR-	RSRC2	1.000	0.142	0.29	0.462	0.387
R.319 Spleen MLR+ vs MLR-	SRSF7	1.000	0.150	0.35	0.615	0.624
R.319 Spleen MLR+ vs MLR-	ID3	1.000	0.159	-0.32	0.154	0.249
R.319 Spleen MLR+ vs MLR-	MKI67	1.000	0.181	-0.27	0.051	0.123
R.319 Spleen MLR+ vs MLR-	FOSB	1.000	0.211	0.38	0.718	0.794
R.319 Spleen MLR+ vs MLR-	ENSMMUG00000051392	1.000	0.228	-0.34	0.436	0.489
R.319 Spleen MLR+ vs MLR-	JUN	1.000	0.251	0.51	0.872	0.829
R.319 Spleen MLR+ vs MLR-	NONO	1.000	0.277	0.26	0.436	0.396
R.319 Spleen MLR+ vs MLR-	DUSP1	1.000	0.287	-0.26	0.846	0.849
R.319 Spleen MLR+ vs MLR-	ZNF800	1.000	0.293	0.26	0.256	0.194
R.319 Spleen MLR+ vs MLR-	ACTG1	1.000	0.322	-0.28	1	0.995
R.319 Spleen MLR+ vs MLR-	TNFSF9	1.000	0.323	0.29	0.179	0.129
R.319 Spleen MLR+ vs MLR-	PSMA5	1.000	0.344	0.25	0.359	0.332
R.319 Spleen MLR+ vs MLR-	GZMA	1.000	0.371	-0.40	0.179	0.127
R.319 Spleen MLR+ vs MLR-	ENSMMUG00000062077	1.000	0.421	0.26	1	0.999
R.319 Spleen MLR+ vs MLR-	DDIT3	1.000	0.511	0.25	0.205	0.174
R.319 Spleen MLR+ vs MLR-	HMGB2	1.000	0.736	-0.31	0.436	0.449

Supplementary Data 4: Rhesus TCR Reference

```
>1|TRAV1-1*01 IMGT000013|TRAV1-1|L-REGION+V-REGION|TR|TRA|None|01
ATGTGGGGAGCTTTCCTTCTTTATGTCTCCATGAAGATGGGAGGCACTGCAGGACAAAGCCTTGAGCAGC
CCTCTGAAGTGACAGCTGTGGAAGGAGCCATTGTCCAGATAAACTGCACGTACCAGACATCTAGGTTTGA
TGGGCTGTCTTGGTACCAGCAACATGATGGCGGAGCACCCACATTTCTTTCTTACAATGCTCTGGATGGT
TTGGAGGAGAGAGGTCATTTTTCTTCATTCCTTAGTCGCTCTGATAGTTATGGTTACCTCCTTCTACAGG
AGCTCCAGATGAAAGACTCTGCCTCTTACTTCTGTGCTGTGAGAGA
>2|TRAV1-1*02 IMGT000076|TRAV1-1|L-REGION+V-REGION|TR|TRA|None|02
ATGTGGGGAGCTTTCCTTCTTTATGTCTCCATGAAGATGGGAGGCACTGCAGGACAAAGCCTTGAGCAGC
CCTCTGAAGTGACAGCTGTGGAAGGAGCCATTGTCCAGATAAACTGCACATACCAGACATCTAGGTTTGA
TGGGCTGTCTTGGTACCAGCAACATGATGGCGGAGCACCCACATTTCTTTCTTACAATGCTCTGGATGGT
TTGGAGGAGAGAGGTCATTTTTCTTCATTCCTTAGTCGCTCTGATAGTTATGGTTACCTCCTTCTACAGG
AGCTCCAGATGAAAGACTCTGCCTCTTACTTCTGTGCTGTGAGAGA
>3|TRAV1-2*01 IMGT000013|TRAV1-2|L-REGION+V-REGION|TR|TRA|None|01
ATGTGGGGAGCTTTCCTTCTTTACATTTCCATGAAGATGGGAGTCACTACAGGACAAAACATTGACCAGC
CCACTGAGATGACAGCTATGGAAGGAGTCATTGTCCAGATCAACTGCACGTACCAGACGTCTGGGTTCAA
TGGGCTGTCCTGGTACCAGCAACATGATGGCGAAGCACCCACACTTCTCTCTTACAATGTTCTGGATGGT
TTGGAGGAGAAAGGTCGTTTTTCTTCATTCCTTAGTAACTCGAAAAGGTACAGTTACCTCCTTTTGAAGG
AGCTCCAGATGAAAGACTCTGCCTCTTACCTCTGTGCTGTGAGAGA
>4|TRAV10*01 IMGT000013|TRAV10|L-REGION+V-REGION|TR|TRA|None|01
ATGAAGAAGCGTCTGAGGACCTGCTTGGTGATTTTGTGGCTTTATTTTTATAGGGGAAGTGGCAAAAACC
AAGTGGAGCAGAGTCCTCAGTCCCTGATCATCCTAGAGGGAAAGAACTGCACTCTTCAATGCAATTATAC
AGTGAGCCCCTTCAGCAACTTAAGGTGGTATAAGCAAGATACTGGGAAAGGTCCCATTTCCCTGACAATC
ATGACTTTCAGTGAGAACACAAAGTCGAATGGAAGGTACACAGCAACTCTGGATGCAGACACCAAGCAAA
GCTCTCTGCACATCACAGCCGCCCAGCTCAGTGATTCAGCCTCCTACATCTGTGTGGTGAGCG
>5|TRAV11-1*01 IMGT000013|TRAV11-1|L-REGION+V-REGION|TR|TRA|None|01
ATGGAGAAGCCCTTGGGAGTTTCCTTCTTGATTTTCTCCTGGCAGCTGTGCTCCGCCCATCTCGGCCTCC
CAAAGTGCTGGGATTACAGGCGTGAGCCACCACGCCCGGCTATGCATGCCATTCTTAATTGTACTTATCA
GGTGAGAACACTCTTCAATTTCCACTGGTTCCTGCAGGATCCAGGGAGAGGATTTGTGTCTTTGACCTTA
ATTCAATCAAGCCAGAAGGAGCAAGGAGACAAATACTGGAGTCAAGAACTGCTTGGAAAAGAGAAGTTTT
ATAGTGTTTGGAATCTCCCACTCTCTCATCCTGGAGATTCAGTCACCTACTTCTGTGCTTTGT
>6|TRAV12-1*01 IMGT000013|TRAV12-1|L-REGION+V-REGION|TR|TRA|None|01
ATGATGAAATCCTTGAGAGTTTTACTGGTGATCCTGTGGCTTCAGTTAAGCTGGGTTTGGAGCCAACAGA
AGGAGGTGGAGCAGGATTCTGGACCCTTCAGTGTTCCAGAGGGAGCCACTGTCACTTTCAACTGCACTTA
CAGCAACAGTGCTTCCCAGTCTTTCTTCTGGTACAGACAGGATTCCAGGAAAGAACCTAAGTTGCTGATG
TCTGTATACTCCAGTGGTAACGAAGATGGAAGGTTTACAGCACAGCTCAATAGAGCCAGCCAGTATATTT
CCCTGCTCATCAGAGACTCCCAGCTCAGTGATTCAGCCACCTACCTCTGTGCAGTGCGTA
>7|TRAV12-1*02 IMGT000076|TRAV12-1|L-REGION+V-REGION|TR|TRA|None|02
ATGATGAAATCCTTGAGAGTTTTACTGGTGATCCTGTGGCTTCAGTTAAGCTGGGTTTGGAGCCAACAGA
AGGAGGTGGAGCAGGATCCTGGACCCTTCAGTGTTCCAGAGGGAGCCACCGTCACTTTCAACTGCACTTA
CAGCAACAGTGCTTCCCAGTCTTTCTTCTGGTACAGACAGGATTCCAGGAAAGAACCTAAGTTGCTGATG
TCTGTATACTCCAGTGGTAACGAAGATGGAAGGTTTACAGCACAGCTCAATAGAGCCAGCCAGTATATTT
CCCTGCTCATCAGAGACTCCCAGCTCAGTGATTCAGCCACCTACCTCTGTGCAGTGCGTA
>8|TRAV12-2*01 IMGT000013|TRAV12-2|L-REGION+V-REGION|TR|TRA|None|01
ATGATGAAATCCTTGAGAGTTTTACTAGTGATCCTGTGGCTTCAGTTGAGCTGGGTTTGGAGCCAACAGA
AGGTGGAGCAGAATTCTGGACCCCTCAATGTTCCAGAGGGAGCCATTGCCTCTCTCAACTGCACTTATAG
TGACCGTGGTTCTCAGTCCTTCTTCTGGTACAGACAATATTCTGGGCAAAGCCCTGAGTTGCTAATGTCC
ACATACTCAAGTGGTGACAAAGAAGATGGAAGATTTACAGCACAGCTCAATAAAGCCAGCCAGTATGTTT
CTCTGCTCATCAGAGACTCCCAGCTCAGTGATTCAGCCACCTACCTCTGTGCCGTGAACA
>9|TRAV12-3*01   IMGT000013|TRAV12-3|L-REGION+V-REGION|TR|TRA|None|01
```

ATGATGAAATCCTTGAGAATTTTACTGGTGATCCTGTGGCTTCAGTTAAGCTGGGTTTGGAGCCAACAGA
AGGAGGTGGAGCAGAATCCTGGACCCCTCAGTGTTCCAGAGGGAGCCACTGCCTCTCTCAACTGCACTTA
CAGCAACAGTGCTTTTCAATACTTCATGTGGTACAGACAGTATTCCAGAAAAGGCCCTCAGTTGCTGATT
TACATATACTCCAGTGGTAACAAAGAAGATGGAAGGTTTACAGCACTGGTCGATAAATCCAGCAAGTATA
TTTCCTTGTTCATCAGAGACTCACAGCCCAGTGATTCAGCCACCTACTTCTGTGCAATGAGCG
>10|TRAV13-1*01 IMGT000013|TRAV13-1|L-REGION+V-REGION|TR|TRA|None|01
ATGACATCCATTCGAGCTGTATTTATATTCTTGTGGCTGCAGCTGGACTCGGTGAATGGAGAGAATGTGG
AGCAGCATCCTTCAACCCTGAGTGTCCAGGAGGGAGACAGCTCCGTTATCAAGTGTACTTATTCAGACAG
TGCCTCAAACTACTTCCCTTGGTATAAGCAAGAACTTGGAAAAGGACCTCAGTTCATTATAGACATTCGT
TCAAATGCGCATGAAAAGAAAAACCAAAGAATTACTGTTTTATTGAACAAGACAGCCAAACATTTCTCTC
TGCACATCACAGAGACCCAACCGGGAGACTCAGCTGTCTACTTCTGCGCAGCGAGTA
>11|TRAV13-1*02 IMGT000076|TRAV13-1|L-REGION+V-REGION|TR|TRA|None|02
ATGACATCCATTCGAGCTGTATTTATATTCTTGTGGCTGCAGCTGGACTCGGTGAATGGAGAGAATGTGG
AGCAGCATCCTTCAACCCTGAGTGTCCAGGAGGGAGACAGCTCTGTTATCAAGTGTACTTATTCAGACAG
TGCCTCAAACTACTTCCCTTGGTATAAGCAAGAACTTGGAAAAGGACCTCAGTTCATTATAGACATTCGT
TCAAATGAGCATGAAAAGAAAGACCAAAGAATTACTGTTTTATTGAACAAGACAGTCAAACATTTCTCTC
TGCACATCACAGAGACCCAACCTGGAGACTCAGCTGTCTACTTCTGCGCAGCGAGTA
>12|TRAV13-2*01 IMGT000013|TRAV13-2|L-REGION+V-REGION|TR|TRA|None|01
ATGGCATGCATTCGAGCTTCATTTATGTACTTGTGGCTGCAGCTGGACTGGGTGAGCAGAGGAGAGAGTG
TGGGGCTGCATCTTCCTACCGTGAGTGTTCAGGAGGGAGACAACTCTGTTATCAACTGTACTTATTCAGA
CAGTGCCTCAGACTACTTCTTTTGGTACAAGCAGGAATCTGGAAAAGGTCCTCAATCCATTATGGACATC
CGTTCAAATATGGCTAAGAGGCAAGGCCAAAGACTCACCGTTTTATTGAATAAGACAATGAAACATCTCT
CTCTGCAAATTGCAGCTACTGAACCTGGAGACTCAGCTGTCTACTTCTGTGCAGAGAATA
>13|TRAV13-2*02 IMGT000076|TRAV13-2|L-REGION+V-REGION|TR|TRA|None|02
ATGGCATGCATTCGAGCTTCATTTATGTACTTGTGGCTGCAGCTGGACTGGGTGAGCAGAGGAGAGAGTG
TGGGGCTGCATCTTCCTACCGTGAGTGTTCAGGAGGGAGACAACTCTGTTATCAACTGTACTTATTCAGA
CAGTGCCTCAGACTACTTCCTTTGGTATAAGCAGGAATCTGGAAAAGGTCCTCAATCCATTATGGACATC
CGTTCAAATATGGCTAAGAGGCAAGGCCAAAGACTCACCGTTTTATTGAATAAGACAATGAAACATCTCT
CTCTGCAAATTGCAGCTACTGAACCTGGAGACTCAGCTGTCTACTTCTGTGCAGAGAATA
>14|TRAV14-2*01 IMGT000013|TRAV14-2|L-REGION+V-REGION|TR|TRA|None|01
ATGTTACTTTCTAGCCTGCTGAAGGTGGTCACAGCTTCACTCTGGCTAGGATCTGGCATTGCCCAGAAGA
TAACTCAAACCCAACCAGCAATGTTCGTGCAGGAAAAGGAGGCTGTGACTCTGGACTGCACATATGACAC
CAGTGATCAAAATTACGGTCTATTCTGGTACAAGCAACCCAGCAGTGGAGAGATGATTTTTCTTATTCTT
CAGATGTCTTATGACAAGCAAAATGCAACAGAAGGACGCTACTCATTGAACTTCCAGAAGGCAAGAAAAT
CCGTCAACCTTGTCATCTCTGCTTCACAAGTGGGGGACTCAGCAACGTATTTCTGTGCAATGAGAGGG
>15|TRAV16*01 IMGT000013|TRAV16|L-REGION+V-REGION|TR|TRA|None|01
ATGAAGCCCACCCTCATCTCAGTGCTTGTGATAATATTTATACTCAGAACAAGAGCCCAGAGTGTGACTC
AGCCCGAGAAGCTCCTCTCTGTCTTTAAAGGGGCCCCAGTGGAACTGAAGTGCTACTATTCATATTCTGG
GAGTCCTAATCTCTTCTGGTATGTCCAGTACCCCAAACAACGCCTCCAGTTACTCTTGAGACACATCTCT
AGAGAGAGCATCAAAGGCTTCACTGCTGACCTTAACAAAAGCGATACATCTTTCCATCTGAAGAAACTAT
TCGCTCAAGAGGAAGACTCAGCCACGTATTACTGTGCTCTAAGTGG
>16|TRAV17*01 IMGT000013|TRAV17|L-REGION+V-REGION|TR|TRA|None|01
ATGGAAACTCTCCTGGGAGTGTCTTTGGTGATTCTATGGCTTCAACTGGCCAGGGTGAACAGTCAACAAG
GAGAAGAGGATCCTCAGGCCTTGAGCATCCAGGAGGGTGAAAATGCCACCATGAACTGCAGTTACAAAAC
TAGTATAAACAATTTACAGTGGTATAGACAAGATTCAGGTAGAAGCCTTGTACAGCTAGTTTTAATACGT
TCAAATGAAAGAGAGAAAAATAGCGGAAGACTAAGAGTCACTCTTGACACTTTGAAGAAAAGCAGTTCCT
TGTTGATCACGGCTTCCCGGGCAGCAGACACTGCTTCTTACTTCTGTGCTACGGATG
>17|TRAV18*01 IMGT000013|TRAV18|L-REGION+V-REGION|TR|TRA|None|01
ATGCTGTTTGCTTCCTGCTCAGGACTTGTGATCTTGTTGATAATCAGAAGGACCAATGGAGACTCAGTGA
CCCAGACAGAAGGTCCAGTTACCCTCCCTGAGAGGGCAGCTCTAACATTAAACTGCACTTATCAGACCAG

CTATTCAGCTTTTATATTCTGGTATGTCCAGTATCCAAACAAAGAGCCTGAGCTCCTCCTGAAAAGTTCA
GAAAACCAGGAGACGAACAGCAGAGGTTTTCAGGCCAGTCATGTCAAGAGTGACAGTTCCTTCCATCTGG
AGAAACCCTCGGTGCAGCTGTCGGACTCTGCCGTGTACTACTGTGTTCTGAGAGA
>18|TRAV19*01 IMGT000013|TRAV19|L-REGION+V-REGION|TR|TRA|None|01
ATGCTGACTGCCAGCCTGCTGAGGGCAGTCATAACCTCCATCTGTGTTGGATCCAGCATGGCTCAGAAGG
TAACTCAAGCTCAGACTGAAATTTCTGTGGTGGAGAAGGAGGATGTGACCTTGGACTGTGTGTATGAAAC
CCGTGATACTACTTATTACTTATTCTGGTACAAGCAACCACCAAGTGGAGAATTGGTTTTCCTTATTCGT
CAGAACTCTTTTGATGAGCAAAATGAAATAAATGGTCGATATTATTCGAACTTCCAGAAATCCACCAGTT
CCTTCAACCTCACCATCACAGCCTCACAGATCGTGGACTCAGCAGTATACTTCTGTGCTCTGAATGAAGC
>19|TRAV19*02 IMGT000076|TRAV19|L-REGION+V-REGION|TR|TRA|None|02
ATGCTGACTGCCAGCCTGCTGAGGGCAGTCATAACCTCCATCTGTGTTGGATCCAGCATGGCTCAGAAGG
TAACTCAAGCTCAGACTGAAATTTCTGTGGTGGAGAAGGAGGATGTGACCTTGGACTGTGTGTATGAAAC
CCGTGATACTACTTATTACTTATTCTGGTACAAGCAACCACCAAGTGGAGAATTGGTTTTCCTTATTCGT
CAGAACTCTTTTGATGAGCAAAATGAAATAAATGGTCGATATTATTCGAACTTCCAGAAATCCACCAGTT
CCTTCAACCTCACCATCACAGCCTCACAGGTCGTGGACTCAGCAGTATACTTCTGTGCTCTGAATGAAGC
>20|TRAV2*01 IMGT000013|TRAV2|L-REGION+V-REGION|TR|TRA|None|01
ATGGCTCTGCAGAGCACTCTGGGGACAGTGTGGCTGGGACTTCTCCTCAACTCTCTCTGGAATGTTGCAG
AAAGCAAGGACCAAGTGTTTCAGCCTTCCACAGTGGCATCTTCGGAGGGAGCCATGGTGGAAATCTTCTG
TAATCACTCTGTGTCCAATGCTTACAACTTCTTCTGGTACCTTCACTTCCCGGGATGTGCACCAAGACTC
CTTGTTAAGGGCTCAAAGCCTTCTCAGCAGGGACGATACAACATGACCTATGAACGATTCTCTTCATCGC
TGCTCATCCTCCAGGTGCGGGAGGCAGATGCTGCTGTTTACTACTGTGCTGTGGAGGA
>21|TRAV20*01 IMGT000013|TRAV20|L-REGION+V-REGION|TR|TRA|None|01
ATGGAGAAAATGTTGGAGTGTGCATTCATAGTCTTGTGGCTTCAGCTTGGCTGGTTGAGTGGAGAAGACC
AGGTGACGCAGAGTCCTGAGGCCCTGAGCCTCCAGGAGGGAGACAGTAGCAGTCTCAACTGCAGTTACAC
AGTCAGCGGCTTAAGAGGGCTGTTCTGGTATAGGCAAGATCCTGGGAAAGGCCCTGAATTCCTCTTCAGC
CTGTATTCAGCTGGGGAAGAAAAGGAAAAAGAAAGGCTAAAAGCCACATTAACGAAGAAGGAGAGCTTTC
TGCACATCACAGGCCCCAAACCTGAAGACTCAGCCACTTATCTCTGTGCTGTGCAAG
>22|TRAV20*02 IMGT000076|TRAV20|L-REGION+V-REGION|TR|TRA|None|02
ATGGAGAAAATGTTGGAGTGTGCATTCATAGTCTTGTGGCTTCAGCTTGGCTGGTTGAGTGGAGAAGACC
AGGTGACGCAGAGTCCTGAGGCCCTGAGCCTCCAGGAGGGAGAGAGTAGCAGTCTCAACTGCAGTTACAC
AGTCAGCGGCTTAAGAGGGCTGTTCTGGTATAGGCAATATCCTGGGAAAGGCCCTGAATTCCTCTTCATC
CTGTATTCAGCTGGGGAAGAAAAGGAAAAAGAAAGGCTAAAAGCCACATTAACGAAGAAGGAGAGCTTTC
TGCACATCACAGGCCCCAAACCTGAAGACTCAGCCACTTATCTCTGTGCTGTGCAAG
>23|TRAV21*01 IMGT000013|TRAV21|L-REGION+V-REGION|TR|TRA|None|01
ATGGAGACCCTCTTGGGCCTGCTCATCCTTTGGCTGCAGTTGCAATGGGTGAGCAGTAAACAGGAGGTGA
CACAGATTCCTGCAGCTCTGAGTGTTCCAGAAGGAGACAACTTGGTTCTCAACTGCAGTTTCACTGATAG
TGCTATTTACAACCTCCAGTGGTTTAGGCAGGACCCTGGGAAAGGTCTCACATCTCTGTTGTTAATTCAG
TCAAGTCAGAGAGAGCAAACAAGTGGAAGACTTAATGCCTCGCTGGATAAATCATCAGGACGTAGTACTT
TATACATTACAGCTTCTCAGCCTAGTGACTCAGCCACCTACCTCTGTGCTGTGAGA
>24|TRAV22-1*01 IMGT000013|TRAV22-1|L-REGION+V-REGION|TR|TRA|None|01
ATGAAGAGGACATTGGGAGCTCTGCTGGGGCTCTTCAGTGCCCAGGTTTGCTGTGTGATAGGAATTCAAG
TGGAGCAGAGTCCTCCAGACCTGATTCTCCAGGAGGGAGCCAATTCTACGCTGCGGTGCAATTATTCTGC
CACTGTGAACAATTTGCAGTGGTTTCACCAAAACCCTTGGGGACAGCTCATCAACCTGTTTTACATAGCA
TCAGGGACAAAACAGAATGGAAGATTAAGCGCCACGACTGTCACTACGGAACGCTACAGCTTATTGCACA
TTTCCTCTTCCCAGACCACAGACTCAGGCATTTATTTCTGTGCTGTGAAGT
>25|TRAV22-1*02 IMGT000076|TRAV22-1|L-REGION+V-REGION|TR|TRA|None|02
ATGAAGAGGACATTGGGAGCTCTGCTGGGGCTCTTCAGTGCCCAGGTTTGCTGTGTGAGAGGAATACAAG
TGGAGCAGAGTCCTGCAGACCTGATTCTCCAGGAGGGAGCCAATTCCAAGCTGCGGTGCAATTTTTCTGC
CACTGTGAGCAATTTGCAGTGGTTTCACCAAAACCCTTGGGGACAGCTCATCAACCTGTTTTACATCCCC
TCAGGGACAAAACAGAATGGAAGATTAAGCGCCACGACTGTCACTACGGAACGCTACAGCTTATTGCACA

TTTCCTCTTCCCAGACCACAGACTCAGGCATTTATTTCTGTGCTGTGAAGT
>26|TRAV22-2*01 IMGT000013|TRAV22-2|L-REGION+V-REGION|TR|TRA|None|01
ATGAAGAGGACATTGGGAGCTCTGCTGGGGCTCTTCAGTGCCCAGGTTTGCTGTGTGATAGGAATTCAAG
TGGAGCAGAGTCCTCCAGACCTGATTCTCCAGGAGGGAGCCAATTCTACGCTGCGGTGCAATTATTCTGC
CACTGCGAACAATTTGCAGTGGTTTCACCAAAACCCTTGGGGACAGCTCATCAACCTGTTTTACATAGCA
TCAGGGACAAAACAGAATGGAAGATTAAGCGCCACGACTGTCACTACGGAACGCTACAGCTTTTTGCACA
TTTCCTCTTCCCAGACCACAGACTCAGGCATTTATTTCTGTGCTGTGGAGT
>27|TRAV22-3*01 IMGT000076|TRAV22-3|L-REGION+V-REGION|TR|TRA|None|01
ATGAAGAGGACATTGGGAGCTCTGCTGGGGCTCTTGAGTGCCCAGGTTTGCTGTGTGAGAGGAATACAAG
TGGAGCAGAGTCCTCCAGACCTGATTCTCCAGGAGGGAGCCAATTCCACGCTGCGGTGCAATTTTTCTGC
CACTGTGAACAATTTGCAGTGGTTTCACCAAAACCCTTGGGGACAGCTCATCAACCTGTTTTACATCCCC
TCAGGGACAAAACAGAATGGAAGATTAAGCGCCACGACTGTCACTAAGGAACGCTACAGCTTATTGCACA
TTTCCTCCTCCCAGACCACAGACTCAGGCATTTATTTCTGTGCTGTGGAGT
>28|TRAV23-1*01 IMGT000013|TRAV23-1|L-REGION+V-REGION|TR|TRA|None|01
ATGGACAAGATCTTAGGAGCATCATTTTTAATTCTGTGGCTTCAACTATGCTGGGTGAGTGGCCAACAGA
AGGAGGAAAGTGACCGGCAGCAGGTGAAACAAAGTCCTCAATCTTTGATAGTCCAGAAAGGAGGGATTTC
AATTATAAACTGTGCTTATGAGAACAGTGCATTTGACTACTTTCCATGGTACCGACAATTCCCTGGGAAA
GGCCCTGCATTATTGATAGCCATAAGTTCAGCTGCGAGCAAAAAAGAAGAAGGAAGATTCAAAATCTTCT
TCAATAAAAGTGCCAAGAACTTCTCATTGCATATCATGGATTCCCAGCCTGGAGACTCAGCCACCTACTT
CTGTGCAGCAAGCA
>29|TRAV23-1*02 IMGT000076|TRAV23-1|L-REGION+V-REGION|TR|TRA|None|02
ATGGACAAGATCTTAGGAGCATCATTTTTAATTCTGTGGCTTCAACTATGCTGGGTGAGTGGCCAACAGA
AGAAGGAAAGTGACCGGCAGCAGGTGAAACAAAGTCCTCAATCTTTGATAGTCCAGAAAGGAGGGATTTC
AATTATAAACTGTGCTTATGAGAACAGTGCATTTGCCTACTTTCCATGGTACCGACAATTCCCTGGGAAA
GGCCCTGCATTATTGATAGCCATAAGTTCAGCTGCGAGCGAAAAAGAAGAAGGAAGATTCAAAATCTTCT
TCAATAAAAGTGCCAAGAACTTCTCATTGCATATCATGGATTCCCAGCCTGGAGACTCAGCCACCTACTT
CTGTGCAGCAAGCA
>30|TRAV23-2*01 IMGT000013|TRAV23-2|L-REGION+V-REGION|TR|TRA|None|01
ATGGACAAGATCTTAGGAGCATCATTTTTAATTCTGTGGCTTCAACTATGCTGGGTGAGTGGCCAACAGA
AGGAGGAAAGTGACCGGCAGCAGGTGAAACAAAGTCCTCAATCTTTGATAGTCCAGAAAGGAGGGATTTC
AATTATAAACTGTGCTTATGAGAACAGTGCATTTGACTACTTTCCATGGTACCGACAATTCCCTGGGAAA
GGCCCTGCATTATTGATAGCCATAAGTTCAGCTGCGAGCAAAAAAGAAGAAGGAAGATTCAAAATCTTCT
TCAATAAAAGTGCCAAGAACTTCTCATTGCATATCATGGATTCCCAGCCTGGAGACTCAGCCACCTACTT
CTGTGCAGCAAGCA
>31|TRAV23-3*01 IMGT000013|TRAV23-3|L-REGION+V-REGION|TR|TRA|None|01
ATGGACAAGATCTTAGGAGCATCATTTTTAATTCTGTGGCTTCAACTATGCTGGGTGAGTGGCCAACAGA
AGGAGGAAAGTGACCGGCAGCAGGTGAAACAAAGTCCTCAATCTTTGATAGTCCAGAAAGGAGGGATTTC
AATTATAAACTGTGCTTATGAGAACAGTGCATTTGACTACTTTCCATGGTACCGACAATTCCCTGGGAAA
GGCCCTGCATTATTGATAGCCATACGTTCAGCTGCGAGCAAAAAAGAAGAAGGAAGATTCAAAATCTTCT
TCAATAAAAATGCCAAGAACTTCTCATTGCATATCATGGATTCCCAGCCTGGAGACTCAGCCACCTACTT
CTGTGCAGCAAGCA
>32|TRAV23-4*01 IMGT000076|TRAV23-4|L-REGION+V-REGION|TR|TRA|None|01
ATGGACAAGATCTTAGGAGCATCATTTTTAATTCTGTGGCTTCAACTATGCTGGGTGAGTGGCCAACAGA
AGGAGGAAAGTGACCGGCAGCAGGTGAAACAAAGTCCTCAATCTTTGATAGTCCAGAAAGGAGGGATTTC
AATTATAAACTGTGCTTATGAGAACAGTGCATTTGACTACTTTCCATGGTACCGACAATTCCCTGGGAAA
GGCCCTGCATTATTGATAGCCATAAGTTCAGCTGCGAGCGAAAAAGAAGAAGGAAGATTCAAAATCTTCT
TCAATAAAAGTGCCAAGAACTTCTCATTGCATATCATGGATTCCCAGCCTGGAGACTCAGCCACCTACTT
CTGTGCAGCAAGCA
>33|TRAV24*01 IMGT000013|TRAV24|L-REGION+V-REGION|TR|TRA|None|01
ATGGAGAAGAATTCTTTGGCAGCCCCATTACTAATCCTCTGGCTTCATCTTGACTGCGTGAGCAGCATCC

TGAACGTGGAACAAAGTCCCCAGTCACTGCATGTTCAGGAGGGAGACAGCACCAACTTCACCTGCAGCTT
CCCTTCCAGCAATTTTTATGCCTTGCACTGGTACAGATTGGAAACTGCAAAAATCCCCAAGGCCTTGTTT
GTAATGACTTTAAATGGGGATGAAAAGAAGAAAGGACGAGTAAGAGTCACTCTTAATACCAAGGAGGGTT
ACAGCTATTTGTACATCAAAGGATCCCAGCCTGAAGACTCAGCCACATACCTCTGTGCCTTTG
>34|TRAV24-1*01 IMGT000076|TRAV24-1|L-REGION+V-REGION|TR|TRA|None|01
ATGGAGAAGAATCCTTTGGCAGCCCCATTACTAATCCTCTGGCTTCATCTTAACTGCGTGAGCAGCATCC
TGAACGTGGAACAAAGTCCCCAGTCACTGCATGTTCAGGAGGGAGACAGCACCAACTTCACCTGCAGCTT
CCCTTCCAGCAATTTTTATGCCTTGCACTGGTACAGATGGGAAACTGCAAAAATCCCCAAGGCCTTGTTT
GTAATGACTTTAAATGGGGATGAAAAGAAGAAAGGACGAGTAAGTGTCACTCTTAATACCAAGGAGGGTT
ACAGCTATTTGTACATCAAAGGATCCCAGCCTGAAGACTCAGCCACATACCTCTGTGCCTTTG
>35|TRAV25*01 IMGT000013|TRAV25|L-REGION+V-REGION|TR|TRA|None|01
ATGCTACTCATCACATCAGTGTTGGTCTTATGGATGCAATTGTCACAGGTGAATGGACAACAAATAATGC
AAATTCCTCAGTACCAGCATGTACAAGAAGGAGAGGACTTCACCACGTACTGCAATTCCTCAACTACTTT
AAGCAATATACAGTGGTATAAGCAAAGGCCTGGGGGACATCCCGTTTTTTTGATAATGTTAGTGAAGAGT
GGAGAAGTGAAGAAGCAGAAAAGACTGACATTTCAGTTTGGAGAAGCAAAAAAGAACAGCTCCCTGCACA
TCACAGCCACCCAGACTACAGATGTAGGAACCTACTTCTGCGCAGGG
>36|TRAV25*02 IMGT000076|TRAV25|L-REGION+V-REGION|TR|TRA|None|02
ATGCTACTCATCACATCAGTGTTGGTCTTATGGATGCAATTGTCACAGGTGAATGGACAACAAATAATGC
AAATTCCTCAGTACCAGCATGTACAAGAAGGAGAGGACTTCACCACGTACTGCAATTCCTCAACTACTTT
AAGCAATATACAGTGGTATAAGCAAAGGCCTGGGGGACATCCCGTTTTTTTGATAACGTTAGTGAAGAGT
GGAGAAGTGAAGAAGCAGAAAAGACTGACATTTCAGTTTGGAGAAGCAAAAAAGAACAGCTCCCTGCACA
TCACAGCCACCCAGACTACAGATGTAGGAACCTACTTCTGCGCAGGG
>37|TRAV25-1*01 IMGT000076|TRAV25-1|L-REGION+V-REGION|TR|TRA|None|01
ATGCTACTCATCACATCAGTGTTGGTCTTATGGATGCAATTGTCACAGGTGAATGGACAACAAATAATGC
AAATTCCTCAGTACCAGCATGTACAAGAAGGAGAGGACTTCACCACGTACTGCAATTTCTCAACTACTTT
AAACAATATACAGTGGTATAAGCAAAGGCCTGGGGGACATCCTGTTTTTTTGATAATGTTAGTGAAGAGT
GGAGAAGTGAAGAAGCAGAAAAGACTGACATTTCAGTCTGGAGAAGCAAAAAAGAACAGCTCCCTGCACA
TCACAGCCACCCAGACTACAGATGTAGGAACCTACTTCTGCGCAGGG
>38|TRAV26-1*01 IMGT000013|TRAV26-1|L-REGION+V-REGION|TR|TRA|None|01
ATGGAATACAGCCTAGAGAGTCTATTATTATTGGCATGTATCTTTGGGACTATAATTGATGCTAAGACGA
CCCAACCCACCTCCATGGATTGTGCTGAAGGAAGAGCTGCAAACCTGCCTTGTAATCACTCTACCATCGG
TGGAAATGAGTACATACATTGGTATCGACAGATTCACTCCGAGGGGCCACAGTATGTCATTCATGGTCTA
AAAAACAATGAGACCAATGCAATGGCCTCTCTGATCATCACAGAAGACAGAAAGTCCAGCACCTTGATCC
TGCCCCACGCGACGCTGAGAGACACTGCTGTGTACTATTGCATCGTGAGAGTCG
>39|TRAV26-2*01 IMGT000013|TRAV26-2|L-REGION+V-REGION|TR|TRA|None|01
ATGATTTACCAATTGCTGGAAAGAATTTGTATCTATGTGTTTCTCAAGGGTATTAGGGGTGATGCTAAGA
CCACACAGCCAAATTCAATGGAGAGTAATGAGGAAGAGCCTGTACACTTGCCTTGTAACCACTCCACAAT
CAGTGGAACTGATTACATATACTGGTATCGACAACGTCCCTCCCAGGGTCCAGAGTATGTGATTCATGGT
CTTACAAGTAATGTGAACAACAGAATGGCCTCTCTGACAATCGCTGAAGACAGAAAGTCCAGTACTTTGA
TCCTGCACCATGCTACCTTGAGAGATGCTGCTGTGTACTACTGCATCTTGAGAGAC
>40|TRAV26-2*02 IMGT000076|TRAV26-2|L-REGION+V-REGION|TR|TRA|None|02
ATGATTTACCAATTGCTGGAAAGAATTTGTATCTATGTGTTTCTCAAGGGTATTAGGGGTGATGCTAAGA
CCACACAGCCAAATTCAATGGAGAGTAATGAGGAAGAGCCTGTACACTTGCCTTGTAACCACTCCACAAT
CAGTGGAACTGATTACATATACTGGTATCGACAACGTCCCTCCCAGGGTCCAGAGTATGTGATTCATGGT
CTTACAAGTAATAACAACAGAATGGCCTCTCTGACAATCGCTGAAGACAGAAAGTCCAGTACTTTGATCC
TGCACCATGCTACCTTGAGAGATGCTGCTGTGTACTACTGCATCTTGAGAGAC
>41|TRAV26-3*01 IMGT000076|TRAV26-3|L-REGION+V-REGION|TR|TRA|None|01
ATGGAATACAGCCTAGAGAGTCTATTATTATTGGCATGTATCTTTGGGACTATAATTGATGCTAAGACCA
CTCAGCCCACCTCCATGGATTGCGCTGAAGGAAGAGCTGCAAACTTGCCTTGTAATCACTCTACCATCAG
TGGAAGTGAGTACATACATTGGTATCGACAGATTCACTCCCAGGGGCCACAGTATGTCATTCATGGTCTA

AAAAACAATGAGACCAATGCAATGGCCTCTCTGATCATCACAGAAGACAGAAAGTCCAGCACCTTGATTC
TGCCCCACGCGACGCTGAGAGACACTGCTGTATACTATTGCATCGTGAGAGTCG
>42|TRAV27*01 IMGT000013|TRAV27|L−REGION+V−REGION|TR|TRA|None|01
ATGGTCCTGAAATTCTCCGTATCCATTCTTTGGATTCAGTTGGCATGGGTGAGCACCCAGCAGCTGGAGC
AGAGTCCTCGGTTTCTAAGCATCCAAGAGGGAGAAAATTTCACTGCGTACTGCAACTCCTCAAGTGTTTT
TACCAGCTTACAATGGTACAGACAGGACCCTGGGGAAGGTCCTGTCCTCTTGGTGACACTAGTTACGCGT
GGAGAAATGAAGAAGCAGAAGAGACTAACCTTTCAGTTTGGTGATGCAAGAAAGGACAGCTCCCTCCACA
TCACTGCGACCCAGCCTGGTGATACAGGCCTCTACCTCTGTGCAGGAG
>43|TRAV29*01 IMGT000013|TRAV29|L−REGION+V−REGION|TR|TRA|None|01
ATGGGCGTGCTCCTGGGGGCATCATTGCTGATTCTGTGGCTTCAGCCAGACTGGGTAAATAGTCAACAGA
AGAATGATGACCAGCGAGTTAAGCAAAATCCACCATCCCTGAGTGTCCAGGAAGGAGGAATTTCTATTCT
GAACTGTGACTATACTAACAGCATGTTTGATTATTTCGTATGGTACAAAAAATACCCTGCTGAAGGTCCT
ACATTCCTGATATCTATACGTTCCGTTAAGGATAAAAATGAAGAGGGAAGATTCACAGTCTTCTTAAACA
AAAGTGCCAAGCACCTCTCTCTGCACATTGGGGCCTCCCAGCCTGGAGACTCTGCAGTGTACCTCTGTGC
AGCAAGCG
>44|TRAV3*01 IMGT000013|TRAV3|L−REGION+V−REGION|TR|TRA|None|01
ATGGCCTCTGCGCGCATCTCGACGCTTGCGATGCTCTTCACATTGAGTGGGCTGAGAGCTCAGTTGGTGA
CTCAGCCAGAAGATCAGGTCACGGTCGCCGAAGGGAATCCTCTGACTGTGAAATGCACCTATTCAGTCTC
TGGAAACCCTTATCTTTTTTGGTATGTTCAATGCCCCAACCAAGGCCTCCAGTTCCTTCTGAAATACGTC
ACGGGGGACAACCTGGTTAAAGGCAGCTATGGCTTTGAAGCTGAATTTAACAAGAGCCAAACCTCTTTCC
ACCTGAAGAAACCATCTGCCCTTGTGAGCGACTCCGCTTTGTACTTCTGTGCTGTGAGAGA
>45|TRAV30*01 IMGT000013|TRAV30|L−REGION+V−REGION|TR|TRA|None|01
ATGGAGACTCTCCTGAAAGTGCTTTCAAGCATCTTGTTGTGCCAGTTGACCTGGGTGAGAAGCCAACAAC
CAGTGCAGAGTCCTCAAGCCGTGATCCTCCAAGAAGGGGAAGATGCTATCATCAACTGCAATTCCTCAAA
GGCTTTATATTCTGTACTCTGGTACAGGCAGAAGCATGGTGAAGCGCCCATCTTCCTGATGATATTACTG
AAAGGTGGAGAACAGAAGAGTCATGACAAAATAGTCGCTACGTTTAATGAAAAAAAGCAGCAAAGCTCCC
TGTACCTTATGGCTTCCCAGCTCAGTTACTCCGGAACCTACTTCTGTGGTGCAGAGA
>46|TRAV34*01 IMGT000013|TRAV34|L−REGION+V−REGION|TR|TRA|None|01
ATGGAGACTGTTCTGCAAGAACTCCTAGGGATATTGGGGTTCCAAGCAGCCCGGGTCACTAGCCAAGAAC
TGGAGCAGAGTCCTCAGTCCTTGATCGTCCAAGAGGGAAAGAATCTCACCATAAACTGCACATCATCAAA
GACATTATATGGCTTACACTGGTACAAGCAAAAGTATGGTGAAGGTCTTATCTTCTTGATGATGCTACAG
CAAGGTGGGGAAGAGAAAAGTCATGAAAAAATAACTGCCAAGTTGGATGAGAAAAAGCAGCAAAGTTTCC
TGCATATCACAGCCTCCCAGCCCAGCCATGCAGGCATCTACCTCTGTGGAGGAGATG
>47|TRAV34*02 IMGT000076|TRAV34|L−REGION+V−REGION|TR|TRA|None|02
ATGGAGACTGTTCTGCAAGTACTCCTAGGGATATTGGGGTTCCAAGCAGCCCGGATCACTAGCCAAGAAC
TGGAGCAGAGTCCTCAGTCCTTGATCGTCCAAGAGGGAAAGAATCTCACCATAAACTGCACATCATCAAA
GACATTATATGGCTTACACTGGTACAAGCAAAAGTATGGTGAAGGTCTTATCTTCTTGATGATGCTACAG
CAAGGTGGGGAAGAGAAAAGTCATGAAAAAATAACTGCCAAGTTGGATGAGAAAAAGCAGCAAAGTTTCC
TGCATATCACAGCCTCCCAGCCCAGCCATGCAGGCATCTACCTCTGTGGAGGAGACG
>48|TRAV35*01 IMGT000013|TRAV35|L−REGION+V−REGION|TR|TRA|None|01
ATGCTCCTTGAACGTTTATTAATAATCTTGTGGATGCAGCTGACATGGGTCAGTGGTCAACAGCTGAATC
AGAGTCCTCAGTCATTGTCTGTCCAGGAAAGAGAGGATGTCTCCATGAACTGCACTTCTTCAAGCACATT
TAACACCTTTCTATGGTACAAGCAGGACCCTGGGGAAGGTCCTGTCCTCTTGATGGCCTTATTTAAGCCT
GGTGAATTGACCTCAAATGGGAGACTGTCTGCTCAGTTTGGTATAACCAGAAAGGACAGCTTCCTGAATA
TCTCAGCGTCCGTGCCTAGTGATGTAGGCACCTACTTCTGTGCCGGGCAA
>49|TRAV35*02 IMGT000076|TRAV35|L−REGION+V−REGION|TR|TRA|None|02
ATGCTCCTTGAACGTTTATTAATAATCTTGTGGATGCAGCTGACATGGGTCAGTGGTCAACAGCTGAATC
AGAGTCCTCAGTCATTGTCTGTCCAGGAAAGAGAGGATGTCTCCATGAACTGCACTTCTTCAAGCACATT
TAACACCTTTCTATGGTACAAGCAGGACCCTGGGGAAGGTCCTGTCCTCTTGATGGCCTTATTTAAGCCT
GGTGAATTGACCTCAAATGGGAGACTGTCTGCTCAGTTTGGTGTAACCAGAAAGGACAGCTTCCTGAATA

```
TCTCAGCGTCCGTGCCTAGTGATGTAGGCACCTACTTCTGTGCCGGGCAA
>50|TRAV36*01 IMGT000013|TRAV36|L-REGION+V-REGION|TR|TRA|None|01
ATGATGAAGTGTTCACAGGCTTTACTAGCGATCTTTTGGCTTCTACTGAGCTGGGTGAGCAGTGAAGACA
AGGTGATGCAAAACCCTCTATCTCTGGTTGTCCACGAGGGAGACACTGTAACTCTCAACTGCAGTTATGA
AGTGGTTAACTTTCGAAGCCTACTGTGGTACAAGCAGGAAAAGAAAGCTCCCATATTTCTATTTACGCTA
ACTTCAAGTGGAATTGAAAAGAAGTCAGGAAGACTAAGTAGCATATTAGACAAGAAAGACCTTTTCAGCA
TCCTGAACATCACAGCCACCCAGACCAGAGACTCGGCTGTCTACCTCTGTGCTGTGGAGG
>51|TRAV36*02 IMGT000076|TRAV36|L-REGION+V-REGION|TR|TRA|None|02
ATGATGGAGTGTTCACAGGCTTTACTAGCGATCTTTTGGCTTCTACTGAGCTGGGTGAGCAGTGAAGACA
AGGTGATGCAAAACCCTCTATCTCTGGTTGTCCACGAGGGAGACACTGTAACTCTCAACTGCAGTTATGA
AGTGGTTAACTTTCGAAGCCTACTATGGTACAAGCAGGAAAAGAAAGCTCCCATATTTCTATTTACGCTA
ACTTCAAGTGGAATTGAAAAGAAGTCAGGAAGACTAAGTAGCATATTAGACAAGAAAGACCTTTTCAGCA
TCCTGAACATCACAGCCACCCAGACCAGAGACTCGGCTGTCTACCTCTGTGCTGTGGAGG
>52|TRAV38-1*01 IMGT000013|TRAV38-1|L-REGION+V-REGION|TR|TRA|None|01
ATGACACGTGTTAGCTTGCTGTGGGCAGTCGTGGTCTCCACCTGTCTCGAATCCGGCATGGCCCAGACAG
TCACTCAGTCTCAACAAGAGATGTCTGTGCAGGAGGCAGAGACCGTGACCCTGAGCTGCACATATGACAC
CAGTGAGAGTAATTATTATTTGTTCTGGTACAAGCAGCCTCCCAGCAGGCAGATGATTCTCATTATTCGC
CAAGAAGCTTATAAGCAACAGAATGCAACGGAGAACCGCTTCTCTGTGAACTTCCAGAAAGCAGCCAAAT
CCTTCAGTCTCAAGATCTCAGACTCACAGCTGGGGGACGCCGCGATGTATTTCTGTGCTTTCATGAAGCA
>53|TRAV38-1*02 IMGT000076|TRAV38-1|L-REGION+V-REGION|TR|TRA|None|02
ATGACACGTGTTAGCTTGCTGTGGGCAGTCGTGGTCTCCACCTGTCTCGAATCCGGCATGGCCCAGACAG
TCACTCAGTCTCAACAAGAGATGTCTGTGCAGGAGGCAGAGACCGTGATCCTGAGCTGCACATATGACAC
CAGTGAGAGTAATTATTATTTGTTCTGGTACAAGCAGCCTCCCAGCAGGCAGATGATTCTCATTATTCGC
CAAGAAGCTTATAAGCAACAGAATGCAACGGAGAACCGCTTCTCTGTGAACTTCCAGAAAGCAGCCAAAT
CCTTCAGTCTCAAGATCTCAGACTCACAGCTGGGGGACGCCGCGATGTATTTCTGTGCTTTCATGAAGCA
>54|TRAV38-2*01 IMGT000013|TRAV38-2|L-REGION+V-REGION|TR|TRA|None|01
ATGGCATGCCCCGGCTTCCTGTGGACACTTGTGATCTCCACCTGTCTTGAATCCGGCATGGCCCAGACAG
TCACTCAGTCTCAACCAGAGATGTCTGTGCAGGAGGCAGAGACCGTGACCCTGAGCTGCACATACGACAC
CAGTGACAGTGATTATTATTTGTTCTGGTACAAGCAGCCTCCCAGCAGGCAGATGATTCTCATTATTCGC
CAAGAAGCTTATAAGCAACAGAATGCAACAGAGAATCGTTTCTCTGTGAACTTCCAGAAAGCAACCAAAT
CCTTCAGTCTCAAGATCTCAGACTCACAGCTGGGGGACGCCGCGATGTATTTCTGTGCTTATAGGAGCA
>55|TRAV38-2*02 IMGT000076|TRAV38-2|L-REGION+V-REGION|TR|TRA|None|02
ATGGCATGCCCCGGCTTCCTGTGGGCACTTGTGATCTCCACCTGTCTTGAATCCGGCATGGCCCAGACAG
TCACTCAGTCTCAACCAGAGATGTCTGTGCAGGAGGCAGAGACCGTGACCCTGAGCTGCACATATGACAC
CAGTGACAGTGATTATTATTTGTTCTGGTACAAGCAGCCTCCCAGCAGGCAGATGATTCTCATTATTCGC
CAAGAAGCTTATAAGCAACAGAATGCAACAGAGAATCGTTTCTCTGTGAACTTCCAGAAAGCAACCAAAT
CCTTCAGTCTCAAGATCTCAGACTCACAGCTGGGGGACGCCGCGATGTATTTCTGTGCTTATAGGAGCG
>56|TRAV39*01 IMGT000013|TRAV39|L-REGION+V-REGION|TR|TRA|None|01
ATGAAGAAGCTACTAGCAGTGATTCTGTGGCTTCAACTAGACTGGTTAAGTGGAGAGCTGAAAGTGGGAC
AAAACCCTCTGTTCCTGAGCACGCAGGAGGGAAAAAACTATACCATCTACTGCAATTATTCAACCGCTTC
AGACAGACTGTATTGGTACAGGCAGGATCCTGGGAAAAGTCTGGAATCTCTGTTTGTGTTGCTGTCAAAT
GGAGCAGTGAAGCAGGAGGGACGATTAATGGCCTCACTTGACACCAAAGCCCGTCTCAGCAGCCTGCATA
TCACAGCCGCCCTGCAAGACCTCTCTGCCACCTACTTCTGCGCCGTGGACA
>57|TRAV4*01 IMGT000013|TRAV4|L-REGION+V-REGION|TR|TRA|None|01
ATGAGGCAAGTGGCAAGAGTGATCGTGTTCCTGACCCTGAGTACTTTGAGCCTTGCTAAGACCACCCAGC
CCATCTCCATGGACTCATATGAAGGACAAGAAGTGAACATAACCTGTAACCACAACGACATTGCTACAAG
TGATTATATCATGTGGTACCAACAGTTTCCCAACCAAGGACCACGATTTATTATTCAAGGATACAAGGCA
AACATTGCAAATGAAGTGGCCTCCTTGTTTATCCCCACCGACAGAAAGTCCAGCACTCTGAGCCTGCCCC
GGGTTGCCCTGAGTGACACTGCTGTGTACTACTGCCTCGTGGGTGACA
>58|TRAV40*01   IMGT000013|TRAV40|L-REGION+V-REGION|TR|TRA|None|01
```

ATGAACTTCTCTCTGGACTTTCCAATTCTAATCTTAATGTTTGGAGAAACCGGCAGCAATTCAGTCAAAC
AGACAGGCCAAATAACCATCTTGGAGGGAGCATCTGTGACTATGAACTGCACATACACATCTGCGGGGTA
CCCTACCCTTTTCTGGTATGTCCAATACCCCAACAAACCTCTGCAGCTTCTTCAGAGACAGACGATGGAA
AACAGCAAAAACTTTGGAGTCGGAAATATTAAAGACAAAAACTCCCCCATTGTGAAATACTCGGTGCAGG
TATCAGACTCAGCCGTGTACTGCTGTCTTCTGAGAGA
>59|TRAV40*02 IMGT000076|TRAV40|L-REGION+V-REGION|TR|TRA|None|02
ATGAACTTCTCTCTGGACTTTCCAATTCTAATCTTAATGTTTGGAGAAACCGGCAGCAATTCAGTCAAAC
AGACAGGCCAAATAACCATCTTGGAGGGAGCATCTGTGACTATGAACTGCACATACACATCCGCGGGGTA
CCCTACCCTTTTCTGGTATGTCCAATACCCCAACAAACCTCTGCAGCTTCTTCAGAGACAGACGATGGAA
AACAGCAAAAACTTTGGAGTCGGAAATATTAAAGACAAAAACTCCCCCATTGTGAAATACTCGGTGCAGG
TATCAGACTCAGCCGTGTACTGCTGTCTTCTGAGAGA
>60|TRAV41*01 IMGT000013|TRAV41|L-REGION+V-REGION|TR|TRA|None|01
ATGGTGAAGATCCGGCAATTTTTGTTGGCTATTTTGTGGCTTCAGCTAAGCTGTGTAAGTGCCGCCAAAA
ATGAAGTGGAGCAGAGTCCTCAGGACCTGACTGCCCAGGAAGGAGAATTTATCACAATCAACTGCAGTTA
CTCAATAGGAATAAATTCCTTACACTGGCTGCAACAGCATCCAGGAGGAGGCATTGTTTCTTTGTTTATG
CTGAGCTCAGAGAAGAAGAAGAATGGAAGATTAATTGCCACAATAAACATACAGGAAAGGCACAGCTCCC
TGCACATCACAGCCTCCCAGCCCAGAGACTCTGCCATCTACATCTGTGCTGTGAGA
>61|TRAV5*01 IMGT000013|TRAV5|L-REGION+V-REGION|TR|TRA|None|01
ATGAAGACACTTACTGGATCTTTGTTCCTGTTTTTGTGGCTGCAGCTGGACTGTATGAGTAGAGGAGAGG
ATGTGGAGCAGAGTCTTTTCCTGAGTGTCCGAGAGGGAGACATCTCCGTTATAAACTGCACTTACACGGA
CAGTTCCTCCACCTACTTATATTGGTATAAGCAAGAACCTGGAGCAGGTCTCCAGTTGCTGACATATATT
CTTTCAAATATGGACCTGAAACAAGACCAAAGACTCACTGTTCTATTGAATAAGAAGGATAAACATCTGT
CTCTGTGCATCGCAGACACCCAGACTGAGGACTCAGCTATCTACTTCTGTGCAGAGAGTA
>62|TRAV6*01 IMGT000013|TRAV6|L-REGION+V-REGION|TR|TRA|None|01
ATGGAGTCATTCCTGGGAGGTGTTTTGCTGATTTTGTGGCTTCAAGTGGATTGGGTGAAGAGCCAAAAGA
TAGAACAGAATTCCGAGGCCCTGAACATTCAGGAGGGCAAAACGGCCACCCTGACCTGCAACTACACAAA
CTATTCTCCATCATACTTACAGTGGTACCGACAAGATCCAGGAAGAGGCCCTGTTTTCTTGCTACTCATA
CGTGAAAATGAGAAAGAAAAATGGAAAGAAAGACTGAAGGTCACCTTTGATACCACTCTTAAACAGAGTT
TGTTTCATATCACAGCCTCCCAGCCTGCAGACTCAGCTACCTACCTCTGTGCTCTAGACA
>63|TRAV8-1*01 IMGT000013|TRAV8-1|L-REGION+V-REGION|TR|TRA|None|01
ATGCTACTGTTGCTCATACCAGTGCTGGGGATGATTTTTGCCCTGAGAGATGCCAGAGCCCAGTCTGTAA
GCCAGCATAACCACCATGTGATTCTCTCTAAAGGGGCCTCACTGGAGTTAGGATGCAACTATTCCTACGG
TGGCACTGTTAATCTCTTCTGGTATGCCTAGCACCCTGGTCAAGACCTTCCGTTTCTCCTCAAGTACCTT
TCAGGGGACCCATTGGTTAAAGACATCAAAGGCTTTGAGGCTGAATTTATGAAGAGTAAATCCTTCTTTA
ACCTGAGGAAACCCTCTGGGCAGTGGAGTGACATGGCTGAGTACTTCTGTGCCGTGAATGC
>64|TRAV8-2*01 IMGT000013|TRAV8-2|L-REGION+V-REGION|TR|TRA|None|01
ATGCTCCTGCTGCTCGTCCTAGTGCTCGAGGTGATTTTTACCCTGGGAGGAACCAGAGCCCAGTCTGTGA
CCCAGCTTGACAGCCAAGTCTCTGTCTCTGAAGGAGTCCCTGTGCTGCTGAGGTGCAACTACTCATCGTC
TTTTTCACCATATCTCTTCTGGTACGTGCAATACCCCAACCAAGGACTCCAGCTTCTCCTGAAGTACACA
TCAGGGACCACCCTGGTTAAAGGCATCAACGGTTTCGAGGCTGAATTTAAAAAGAGTGAAACCTCCTTCC
ACCTGACGAAAGCCTCAGCCCATGTGAGCGACGCGGCTGAGTACTTCTGTGCTGTGAATGA
>65|TRAV8-3*01 IMGT000013|TRAV8-3|L-REGION+V-REGION|TR|TRA|None|01
ATGCTCCTGCTGCTCGTCCCAGTGCTCGAGGTGATTTTTACCCTGGGAGGAACCAGAGCCCAGTCTGTGA
CCCAGCTTGACAGCCAGGTCTCTGTCTCTGAAGGAGTCCCTGTGCTGCTGAGGTGCAACTACTCATCGTC
TTTTTCACCAAATCTCTTCTGGTATGTGCAATACCCCAACCAAGGTCTCCAGCTTCTCCTGAAGTACATA
TCAGGGACCACGCTGGTTAAAGGCATCAATGGTTTTGAGGCTGAATTTAAGAAGAGTGAAACCTCCTTCC
ACCTGACGAAAGCCTCAGCCCATGTGAGCGACGCGGCTGAGTACTTCTGTGCTGTGAGTGA
>66|TRAV8-4*01 IMGT000013|TRAV8-4|L-REGION+V-REGION|TR|TRA|None|01
ATGCTCCTGGTGTTCATCCCACTGCTGGGGATACATTTTGTCCTGAGAACTGCCAGATCCCAGTCAGTGA
CCCAGCCTGATATCCATATCACCGTCTCTGAAGGAGCCTCACTGGAGTTGAGATGTAACTATTCCTATGG

GGCAACACCTTCTCTCTTCTGGTATGTCCAGTCCCCCGGCCAAGGCCTCCAGCTGCTCCTGAAGTACTTT
TCAGGAGACAGTGTGGTTCAAGGCATTAAAGGCTTTGAGGCTGAATTTAAGAGGAGTCAATATTCCTTCA
ACCTGAGGAAACCCTCTGTGCATTGGAGTGATGCTGCTGAGTACTTCTGTGCTGCGGGTGC
>67|TRAV8-5*01 IMGT000013|TRAV8-5|L-REGION+V-REGION|TR|TRA|None|01
ATGCTCCTGCTGCTCGTCCCAGTGCTCGAGGTGATTTTTACTCTGGGAGGAACCAGAGCCCAGTCTGTGA
CCCAGCTTGACAGCCAAGTCCCTGTCTTTGAAGAAGCCCCTGTGCTGCTGAGGTGCAACTACTCATCATC
GTCTGTTTCAGTGTATCTCTTCTGGTATGTGCAATACCCCAACCAAGGACTCCGGCTTCTCCTGAAGTAT
TTATCAGGACCCACCCTGGTTAAAGGCATCAATGGTTTTGAGGCTGAATTTAAGAAGAGTGAAACTTCCT
TCCACTTGAGGAAACCCTCAGCCCATATAAGCGACACGGCTGAGTACTTCTGTGCTGTGAGTGA
>68|TRAV8-7*01 IMGT000013|TRAV8-7|L-REGION+V-REGION|TR|TRA|None|01
ATGCTCTTAGTGGTCATTCTACTGCTTGGAATGTTCTTCACACTGAGAACCAGAGCCCAGTCGGTGACCC
AGCTTGATGGCCACATCATTGTCTCTGAAAGAGACCCTCTGGAACTGAAGTGCAACTATTCCTATAGTGG
AATTCCTTCTCTCTTCTGGTATGTCCAATACCCCAGCCAAAGCCTTGAGCTTCTCCTCAAGGACCTATCA
GGGGCCACCCAGGTTAAAGGCATCAAAGGTTTTGAGGCTGAATTTAAGAAGAGCGAAACCTCCTTCTACC
TGAGGAAGCCATCAGCCCATGTGAGCGATGCTGCTGAGTACTTCTGTGCTGTGAGTGA
>69|TRAV9-1*01 IMGT000013|TRAV9-1|L-REGION+V-REGION|TR|TRA|None|01
ATGAACTCTTTTCCAGGATCAGTGATTGCACTATTCTTAATGTTTGGGGGAATCAATGGAGATTCAGTGG
TCCAGACAGAAGGCCAAGTGCTCCTCTCTGAAGGGGATTCCCTGATTGTGAACTGCTCCTATGAAAGCAC
ACAGTACCCTTCCCTTTTCTGGTATGTCCAATATCCTGGAGAAGGTCTACAGCTCCTCCTGAAAGCCATG
AAGTTCAATGACAAGGAAAGCAACAAAGGTTTTGAAGCCACATACCATAAAGAAACCACTTCTTTCCACT
TGGAGAAGGACTCAGTTCAAGAGTCAGACTCAGCTGTGTACTTCTGTGCTCTGAGTGA
>70|TRAV9-2*01 IMGT000013|TRAV9-2|L-REGION+V-REGION|TR|TRA|None|01
ATGAACTGTTCTCCAGGCTTAGTATCTGTGATACTCTTACTGCTTGGAAGAACGCGTGGAGATTCAGTGA
CCCAGATGGAAGGGCCAGTGACTCTCTCAGAAAGGGCCTTCCTGACTATAAACTGCACGTACACAGCCAC
AGGATATCCTTCCCTTTTCTGGTATGTCCAATATCCTGGAGAAGGTCCACAGCTCCTCCTGAAAGCCGCG
AAGACTGATGAGAAGGGAAGCAACAAAGGTTTTGAAGCCACATACCGTAAAGAAACCACTTCTTTCCACT
TGAAGAAAGACTCAGTTCAAGAGTCAGATTCAGCTGTGTACTTCTGTGCTCTGAGTGA
>71|TRBV10-1*01   NW_001114291|TRBV10-1|L-REGION+V-REGION|TR|TRB|None|01
ATGGGCACCAGGCTCTTCTTCTATGTGGCCCTTTGTCTGCTGTGGGCAGGACACAGGGATGCTGAAATCA
TCCAGAGCCCAAGACACAAGATCACAGAGACAGGAAGGAAGGTGACCTTGACGTGTCACCAGACTTGGAA
CCACAACAATATGTTCTGGTACCGACAAGACCTGGGACACGGGCTGAGACTGATCCATTACTCATATGGT
GTTCCAGACACTAACAAAGGAGAAGTCCCAGATGGCTACAGTGTCTCTAGATCAAATAAAGAGGATTTCC
CCCTCACTCTGGAGTCTGCTGCCTCCTCCCAGACATCTGTATATTTCTGCGCCAGCAGTGAGTC
>72|TRBV10-1*02 IMGT000012|TRBV10-1|L-REGION+V-REGION|TR|TRB|None|02
ATGGGCACCAGGCTCTTCTTCTATGTGGCCCTTTGTCTGCTGTGGGCAGGACACAGGGATGCTGAAATCA
CCCAGAGCCCAAGACACAAGATCACAGAGACAGGAAGGAAGGTGACCTTGACGTGTCACCAGACTTGGAA
CCACAACAATATGTTCTGGTACCGACAAGACCTGGGACACGGGCTGAGACTGATCCATTACTCATATGGT
GTTCCAGACACTAACAAAGGAGAAGTCCCAGATGGCTACAGTGTCTCTAGATCAAATAAAGAAGATTTCC
CCCTCACTCTGGAGTCTGCTGCCTCCTCCCAGACATCTGTGTATTTCTGCGCCAGCAGTGAGTC
>73|TRBV10-2*01   NW_001114291|TRBV10-2|L-REGION+V-REGION|TR|TRB|None|01
ATGGGCACCAGGCTCTTCTTCTATGTGGCGCTTTGTCTGCTGTGGGCAGGACACAGGGATGCCGGAATCA
CCCAGAGCCCGAGATACAAGGTCACAGAGACAGGAAGGCAGGTGACCTTGACGTGTCACCAGACTTGGAG
CCACAGCTATATGTTCTGGTACCGACAAGATCTGGGACATGGGCTGAGGCTGATCCATTACTCAGCAGGT
GCTGGTATCACAGATAAAGGAGAAGTCCCCGATGGCTATGTTGTCTCCAGATCAAAGACAGAGGATTTCC
TCCTCACTCTGGAGTCAGCTACCCGCTCCCAAACATCTGTGTATTTCTGTGCCAGCAGTGAGTC
>74|TRBV10-3*01   NW_001114291|TRBV10-3|L-REGION+V-REGION|TR|TRB|None|01
ATGGGCACAAGGTTCTTCTTCTATGTGGCCCTTTGTCTCCTGTGGACAGGACACATGGATGCTGGAATCA
CCCAGAGCCCAAGACACAAGATCACAGAGACAGGAACACCAGTGACTCTGAGATGTCACCAGACTGAGAA
CCACCGCTATATGTACTGGTATCGACAAGACCTGGGGAATGGGCTGAGGCTGATCCATTACTCATATGGT
GTTGAAGACACTGACAAAGTAGAAGTCTCAGATGGCTACAGTGTCTCTAGATCAAAGACAGAGGATTTCC

TCCTCACTCTGGAGTCCGCTACCCGCTCCCAGACATCTGTGTACTTTTGTGCCAGCAGTGAATC
>75|TRBV10-3*02 IMGT000012|TRBV10-3|L-REGION+V-REGION|TR|TRB|None|02
ATGGGCACAAGGTTCTTCTTCTATGTGGCCCTTTGTCTCCTGTGGACAGGACACATGGATGCTGGAATCA
CCCAGAGCCCAAGACACAAGATCACAGAGACAGGAACACCAGTGACTCTGAGATGTCACCAGACTGAGAA
CCACCGCTATATGTACTGGTATCGACAAGACCTGGGGAATGGGCTGAGGCTGATCCATTACTCATATGGT
GTTGAAGACACTGACAAAGTAGAAGTCTCAGATGGCTACAGTGTCTCTAGATCAAAGACAGAGGATTTCC
TCCTCACTCTGGAGTCCACTACCCGTTCCCAGACATCTGTGTACTTTTGTGCCAGCAGTGAATC
>76|TRBV11-1*01   NW_001114291|TRBV11-1|L-REGION+V-REGION|TR|TRB|None|01
ATGGGCACCAGGCTCCTCTGCTGGGCGGCCGTCCGTCTCCTGCGGGCAGAATTCACAGAAGCTGGAGTTG
CCCAGTCCCCCAGATATAAGATTACAGAGAAAAGCCAGACTGTGGCTTTTTGGTGTGATCCTATATCTGG
CCATGCTACCCTTTACTGGTACCGGCAGATCCTGGGTCAGGGCCCAGAGCTTCTGGTTCAGTTTCAGAAT
AAGGGTATAGTAGATGATTCACAGTTGCCTAAGGATCGATTTTCTGCAGAGAGGCTCAAAGGAGTAAACT
CCACTCTCAAGATCCAGCCTGCAGAGCTTGGGGACTCGGCCGTGTATCTCTGTGCCAGCAGCTTAGC
>77|TRBV11-2*01   NW_001114291|TRBV11-2|L-REGION+V-REGION|TR|TRB|None|01
ATGGGCACCAGGCTCCTCTGCTGGGCGGCCCTCTGTCTTCTGGGAGCAGAACTCACAGAAGCTGGAGTTG
CCCAGTCCCCCAGATATAAGGTTATAGAAAAAAGCCAGGCTGTGACTTTTTGGTGCAATCCTATATCTGG
CCATGCTACCCTTTACTGGTACCAGCAGATCCTGGGACAGGGCCCGGAGCTTCTGGTTCAATTTCGCAAT
AACGATGTAGTAGATGATTCACAGTTGCCTAAGGATCGATTTTCTGCAGAGAGGCTCAAAGGAGTAGACT
CCACTCTCAAGATCCAGCCTGCAGAGCTTGGGGACTCGGCCGTGTATCTCTGTGCCAGCAGCTTAGC
>78|TRBV11-2*02 IMGT000073|TRBV11-2|L-REGION+V-REGION|TR|TRB|None|02
ATGGGCACCAGACTCCTCTGCTGGGCGGCCCTCTGTCTTCTGGGAGCAGAACTCACAGAAGCTGGAGTTG
CCCAGTCCCCCAGATATAAGGTTATAGAGAAAAGCCAGGCTGTGACTTTTTGGTGCAATCCTATATCTGG
CCATGCTACCCTTTACTGGTACCAGCAGATCCTGGGACAGGGCCCGGAGCTTCTGGTTCAGTTTCGCAAT
AACGATGTAGTAGATGATTCACAGTTGCCTAAGGATCGATTTTCTGCAGAGAGGCTCAAAGGAGTAAACT
CCACTCTCAAGATCCAGCCTGCAGAGCTTGGGGACTCGGCCGTGTATCTCTGTGCCAGCAGCTTAGC
>79|TRBV11-3*01   NW_001114291|TRBV11-3|L-REGION+V-REGION|TR|TRB|None|01
ATGGGCACCGGGCTCCTCTGCTGGGCGGCCCTCTGTCTCCTGGGGGCAGAACTCACAGAAGCTGGAGTGA
CCCAGTCTCCGAGATATAAGATCACAGAGAAAAAACAGCCTGTGGCTTTTTGGTGCAATCCTATTTCTGG
CCACAATACCCTTTACTGGTACCGGCAGAACTTGGGACAGGGCCCGGAGCTTCTGGTTCAATATGAGAAT
GAGGAAGCAGTAGATGATTCACAGTTGCCTAAGGATCGATTTTCTGCAGAGAGGCTCAAAGGAATAGACT
CCACTCTCAGGATCCAGCCTGCAGAGCTTGGGGACTCGGCCGTGTATCTCTGTGCCAGCAGCTTAGC
>80|TRBV12-1*01   NW_001114291|TRBV12-1|L-REGION+V-REGION|TR|TRB|None|01
ATGGACTCCTGGACCCTCTGCGTGTCCCTTTGTATCCTGGTAGCGACATGCACAGATGTTGGCATTATCC
AGTCACCCAAGCATGAGGTGACAGAAATGGGACAAGCAGTGACTCTCAGATGTGAGCCAATTTTAGGCCA
CAATCTCCTTTTCTGGTACAGACAGACCTTCATGCAGGGACTGGAATTGCTGAGTTACTTCTGGAGCCAA
TCTATTATAGATGACGCAGGTATGCCCATGGATGGATTCTCAGCTGAGAGGCCTGATGGACCATTCTCTA
CTTGGAAGATCCAGCCCACAGAGCAGGGGGACTCGGCCGTGTGTGTCTGTGCAAGTCGCTTAGC
>81|TRBV12-1*02 IMGT000073|TRBV12-1|L-REGION+V-REGION|TR|TRB|None|02
ATGGACTCCTGGACCCTCTGCGTGTCCCTTTGTATCCTGGTAGCGACATGCACAGATGTTGGCATTATCC
AGTCACCCAAGCATGAGGTGACAGAAATGGGACAAGCAGTGACTCTCAGATGTGAGCCAGTTTTAGGCCA
CAATCTCCTTTTCTGGTACAGACAGACCTTCATGCAGGGACTGGAATTGCTGAGTTACTTCTGGAGCCAA
TCTATTATAGATGACGCAGGTATGCCCATGGATGGATTCTCAGCTGAGAGGCCTGATGGACCATTCTCTA
CTTGGAAGATCCAGCCCACAGAGCAGGGGGACTCGGCCGTGTGTGTCTGTGCAAGTCGCTTAGC
>82|TRBV12-2*01   NW_001114291|TRBV12-2|L-REGION+V-REGION|TR|TRB|None|01
ATGGGCTTCTGGACTCTCTGCTGTGTGTCCTTTTGTATCCTGGTAGCGAAGCACACAGATGCTGGAGTTA
TCCAGTCACCCCGGCATAAGGTGACAGAGATGGGAAAAGAAGTGACTCTGAGATGCGAACCAATTTCAGG
CCACAGCTCCCTTTTCTGGTACAGACAGACCATGATGCGGGGACTGGAGTTCCTGATTTACTTTAACAAC
AAGTCTCCGATAGATGATTCAGGGATGCCCAAGGATCGATTCTCAGCTACGATGCCTGATGCGTCATTCT
CCACTCTGAAGATTCAGCCCTCAGAACCCAGGGACTCGGCTGTGTACTTCTGTGCCAGCAGTTTAGC
>83|TRBV12-2*02   IMGT000012|TRBV12-2|L-REGION+V-REGION|TR|TRB|None|02

ATGGGCTTCTGGACTCTCTGCTGTGTGTCCTTTTGTATCCTGGTAGCGAAGCACACAGATGCTGGAGTTA
TCCAGTCACCCCGGCATAAGGTGACAGAGATGGGAAAAGAAGTGACTCTGAGATGCGAACCAATTTCAGG
CCACAGCTCCCTTTTCTGGTACAGACAGACCATGATGCGGGGACTGGAGTTCCTGATTTACTTTAACAAC
AAGTCTCCGATAGATGATTCAGGGATGCCCAAGGATCGATTCTCAGCTACGATGCCTGATGCGTCATTCT
CCACTCTGAAGATCCAGCCCTCAGAACCCAGGGACTCGGCTGTGTACTTCTGTGCCAGCAGTTTAGC
>84|TRBV12-2*03 IMGT000073|TRBV12-2|L-REGION+V-REGION|TR|TRB|None|03
ATGGGCTCCTGGACTCTCTGCTGTGTGTCCCTTTGTATCCTGGTAGCGAAGCACACAGATGCTGGAGTTA
TCCAGTCACCCCGGCATAAGGTGACAGAGATGGGAAAAGAAGTGACTCTGAGATGCGAACCAATTTCAGG
CCACAGCTCCCTTTTCTGGTACAGACAGACCATGATGCGGGGACTGGAGTTCCTGATTTACTTTAACAAC
AAGTCTCCGATAGATGATTCAGGGATGCCCAAGGATCGATTCTCAGCTAAGATGCCTGACGCATCATTCT
CCACTCTGAAGATCCAGCCCTCAGAACCCAGGGACTCGGCTGTGTACTTCTGTGCCAGCAGTTTAGC
>85|TRBV12-3*01   NW_001114291|TRBV12-3|L-REGION+V-REGION|TR|TRB|None|01
ATGGGCTCCTGGACTCTCTGCTGTGTGTCCCTTTGTATCCTGGTAGCGAAGCACACAGATGCTGGAGTTA
TCCAGTCACCCCAGCATGAGGTGACAGAGATGGGAAAAGAAGTGACTCTGAGGTGCGAACCAATTTCAGG
CCATACCTACCTCTTCTGGTACAGACAGACCATGATGCGGGGACTGGAGTTCCTGATTTACTTTAACAAC
AAGTCTCCGATAGATGATTCAGGGATGCCCAAGGATCGATTCTCAGCTACGATGCCTGAGGTATCATTCT
CCACTCTGAAGATCCAGCCCTCAGAACCCAGGGACTCGGCTGTGTACTTCTGTGCCAGCAGTTTAGC
>86|TRBV12-3*02 IMGT000073|TRBV12-3|L-REGION+V-REGION|TR|TRB|None|02
ATGGGCTCCTGGACTCTCTGCTGTGTGTCCCTTTGTATCGTGGTAGCGAAGCACACAGATGCTGGAGTTA
TCCAGTCACCCCGGCATGAGGTGACAGAGATGGGAAAAGAAGTGACTCTGAGATGCGAACCAATTTCAGG
CCATACCTACCTCTTCTGGTACAGACAGACCATGATGCGGGGACTGGAGTTCCTGATTTACTTTAACAAC
AAGTATCCGATAGATGATTCAGGGATGCCCAAGGATCGATTCTCAGCTAAGATGCCTGACGCATCATTCT
CCACTCTGAAGATCCAGCCCTCAGAACCCAGGGACTCGGCTGTGTACTTCTGTGCCAGCAGTTTAGC
>87|TRBV12-4*01   NW_001114291|TRBV12-4|L-REGION+V-REGION|TR|TRB|None|01
ATGGCCACCAGGCTCCTCTGCTGTCTGGTCCTTTGTCTCCTGGGAGAAGAGCTCATAGATGCTGGAGTCA
CCCAGACACCAAGGCACAAGGTGACAGAAATGGGACAAGAAGTAACTATGAGATGTCAGCCAATTTTAGG
CCACAATACTGTTTTCTGGTACAGACAGACCGTGATGCAAGGACTGGAGTTGCTGGTTTACTTACGCAAC
AAGGCTTCTCTAGATGATTCAGGAATGCCCAAGGATCGATTCTCAGCAGAGATGCCTGATGCATCTTTAG
CCACTCTGAAGATCCAGCCCTCAGAACCCAGGGACTCGGCTGTGTATCTTTGTGCCAGTGGTTTGGT
>88|TRBV12-4*02 IMGT000073|TRBV12-4|L-REGION+V-REGION|TR|TRB|None|02
ATGGCCACCAGGCTCCTCTGCTGTCTGGTCCTTTGTCTCCTGGGAGAAGAGCTCATAGATGCTGGAGTCA
CCCAGACACCAAGGCACAAGGTGACAGAAATGGGACAAGAAGTAACTATGAGATGTCAGCCAATTTTAGG
CCACAATACTGTTTTCTGGTACAGACAGACCGTGATGCAAGGACTAGAGTTGCTGGTTTACTTACGCAAC
AAGGCTTCTCTAGATGATTCAGGAATGCCCAAGGATCGATTCTCAGCAGAGATGCCTGATGCATCTTTAG
CCACTCTGAAGATCCAGCCCTCAGAACCCAGGGACTCGGCTGTGTATCTTTGTGCCAGTGGTTTGGT
>89|TRBV13*01 NW_001114291|TRBV13|L-REGION+V-REGION|TR|TRB|None|01
ATGCTTGGTCCTGACCTGCCTGACTCTGCCTGGAACACCAGGCTCCTCTGCTATGTCATGCTTTGTCTCC
TGGGAGCAGGTTCAGTGGCTGCTGGAGTCATCCAGTCCCCAAGGCACCTGATCAAAGAAAAGAGGGAAAC
AGCCACTCTGCAATGCTATCCTCTCCCTGAACACGACACTGTCTACTGGTACCAGCAGGGTCCAGGTCAG
GGCCCCCAGTTCCTCATTTCGTTTTATCAAAAGATGCAGAGAGAAAAAGGAAGCATCCCTGATCGATTCT
CAGCTCAACAGTTCAGTGACTATCATTCTGAACTGAACATGAGCTCCTTGGAGCTGGGGGACTCAGCCGT
GTACCTCTGTGCCAGCAACTTAGG
>90|TRBV13*02 IMGT000073|TRBV13|L-REGION+V-REGION|TR|TRB|None|02
ATGCTTGGTCCTGACCTGCCTGACTCTGCCTGGAACACCAGGCTCCTCTGCCATGTCATGCTTTGTCTCC
TGGGAGCAGGTTCAGTGGCTGCTGGAGTCATCCAGTCCCCAAGGCACCTGATCAAAGAAAAGAGGGAAAC
AGCCACTCTGCAATGCTATCCTATCCCTGAACACGACACTGTCTACTGGTACCAGCAGGGTCCAGGTCAG
GGCCCCCAGTTCCTCATTTCGTTTTATCAAAAGATGCAGAGAGAAAAAGGAAGCATCCCTGATCGATTCT
CAGCTCAACAGTTCAGTGACTATCATTCTGAACTGAACATGAGCTCCTTGGAGCTGGGGGACTCAGCCGT
GTACCTCTGTGCCAGCAGCTTAGG
>91|TRBV14*01   NW_001114291|TRBV14|L-REGION+V-REGION|TR|TRB|None|01

ATGGTTTCTAGGCTTCTCAGTTTAGTGTCCCTTTGTCTCCTGGGAGCAAAGCATACAGAAGCTGGAGTTA
CTCAGTTCCCCAGCCACAGGGTAATAGAGAAGGGCCAGGCTGTAACTCTGAGATGTGACCCAATTTCTGG
ACATGATTATCTTTATTGGTATCGACGTGTTATGGGAAAAGAAATAAAATTTCTGATATACTTTCTGAGA
GCGTCTATGCAGGATGAGTCCGGTATGCCCAACAAGCGATTCTCAGCTGAAAGGACTGGAGGGACGTATT
CTACTCTGAAGGTGCAGCCTGCAGAACTGGAGGATTCTGGAGTTTATTTCTGTGCCAGCAGCCAAGA
>92|TRBV15*01 NW_001114291|TRBV15|L-REGION+V-REGION|TR|TRB|None|01
ATGAGTCCTGGGCTTCTCCACTGGATGGCCCTTTGTCTCCTTGGAACAGGTCATGGGGATGTCATGGTCA
TCCAGAACCCAAGATACCAGGTTACTCAGTTGGAAAAGCCAGTGACCTTGAGTTGTTCTCAGAATCTGAA
CCATAAGGTCATGTACTGGTACCAGCAGAAGCCAAGTCAGGCCCCAAAGCTGCTGTTCCACTACTATGAC
AAAGATTTTAACAATGAAGCAGACACCCCTGATAACTTCCAATCCAGGAGGCCAAACACTTCTTTCTGCT
TTCTTGACATCCGCTCACCAGGCCTGGAGGATGCAGCTGTGTACCTGTGTGCCAGCAGCAAAGA
>93|TRBV16*01 NW_001114291|TRBV16|L-REGION+V-REGION|TR|TRB|None|01
ATGAGCCCCATATTCACCTACTTCACAATCCTTTGTCTCCTGGCTGCAAGTTCTCCCGGTGAAGAAGTCA
CCCAGACTCCAAAACATCTTGTCAAAGGGGAAGGACAGAAAGCAAAATTATATTGTGCCCCAATAAAAGG
ATACAGTTACTTTTTTTGGTACCAACGGGTCCTGAAAAAAGAGTTCAAGTTCTTGATTTCCTTCCAGAAT
GAAAATGTCTTTGATGAAACAGGGATGCCCAAGGAAAGATTTTCAGCTAAGTGCCCCCCAAATTCACCCT
GTAGCCTTGAGATCCAGGCTACGAAGCTGAAGGATTCAGCAGCGTATTTTTGTGCCAGCAGCCAATC
>94|TRBV16*02 IMGT000073|TRBV16|L-REGION+V-REGION|TR|TRB|None|02
ATGAGCCCCATATTCACCTACTTCACAATCCTTTGTCTCCTGGCTGCAAGTTCTCCCGGTGAAGAAGTCA
CCCAGACTCCAAAACATCTTGTCAAAGGGGAAGGACAGAAAGCAAAATTATATTGTGCCCCAATAAAAGG
ATACAGTTACTTTTTTTGGTACCAACGGGTCCTGAAAAAAGAGTTCAAGTTCTTGATTTCCTTCCAGAAT
GAAAATGTCTTTGATGAAACAGGGATGCCCAAGGAAAGATTTTCTGCTAAGTGCCCCCCAAATTCACCCT
GTAGCCTTGAGATCCAGGCTACGAAGCTGAAGGATTCAGCAGCGTATTTTTGTGCCAGCAGCCAATC
>95|TRBV18*01 NW_001114291|TRBV18|L-REGION+V-REGION|TR|TRB|None|01
ATGGACACCAGAGTCCTCTCCTGTGTGGTCATCTGTCTTCTGGGGACAGGTCTCTCAAATGCCGGCATCA
CACAGAACCCAAGACACCTGGTCAGGAGGAGGGGACAGGAGGCAAGACTGAGATGCAGCCCAATGAAAGG
ACACAGTCATGTTTATTGGTATCGGCAGCTCCCAGAGGAAGGTCTGAAATTCATGGTTTATCTCCAGAAA
GAAAAAATCATAGATGAGTCAGGAATGCCAAAGGAATGGTTTTCTGCTGAATTTCCCAAAGAAGGCCCCA
GCATCCTGAGGATCCAGCAGGCAGAGCAAGAAGACTCAGCAGCTTATTTCTGTGCCAGCTCACCACC
>96|TRBV19*01 NW_001114291|TRBV19|L-REGION+V-REGION|TR|TRB|None|01
ATGAGCAACCAGGTGCTCTGCTGTGTGGTCCTTTGTCTCCTTGGAGCAAACACCATGGATGGCAGAATCA
CTCAGTCCCCAAAGTACCTGTTCAGAAAGGAAGGACAGAATGTGACCCTGAGTTGTGAACAGAATTTGAA
CCACGATGCCATGTACTGGTACCGACAGGACCCAGGTCAAGGACTGAGATTGATCTACTACTCACAGATA
GTAAATGACATTCAGAAAGGAGACATAGCTGAAGGGTACAGTGTGTCTCGGGAGAGGAAGGAATCCTTTC
CTCTCACTGTGACATCAGCCCAAAGGAACCCAACAGCTTTCTATCTCTGTGCCAGTAGTATAGA
>97|TRBV19*02 IMGT000073|TRBV19|L-REGION+V-REGION|TR|TRB|None|02
ATGAGCAACCAGGTGCTCTGCTGTGTGGTCCTTTGTCTCCTTGGAGCAAACACCATGGATGGCGGAATCA
CTCAGTCCCCAAAGTACCTGTTCAGAAAGGAAGGACAGAATGTGACCCTGAGTTGTGAACAGAATTTGAA
CCACGATGCCATGTACTGGTACCGACAGGACCCAGGTCAAGGACTGAGATTGATCTACTACTCACAGATA
GTAAATGACATTCAGAAAGGAGACATAGCTGAAGGGTACAGTGTGTCTCGGGAGAGGAAGGAATCCTTTC
CTCTCACTGTGACATCAGCCCAAAGGAACCCAACAGCTTTCTATCTCTGTGCCAGTAGTATAGA
>98|TRBV2-1*01 NW_001114291|TRBV2-1|L-REGION+V-REGION|TR|TRB|None|01
ATGGATACCTGGCTCCTATGCTGGGCAATTTTTAGTCTCTTGAAAGCAGGACACACAGAACCTGAAGTCA
CCCAGACTCCCAGCCATCAGGTCACACAGATGGGACAGGAAGTGATCTTGCGGTGTGTCCCCATCCCTAA
TCACTTAAACTTCTATTGGTACAGACAAATCTTGGGGCAGAAAGTCGAGTTTCTGGTTATCTTTTTTGAT
AATAACATCTCAGAGAAGTCTGAAATATTTGAAGATCGATTCTCAGTCGGAAGGCCTGATGGATCAAATT
TCACTCTGAAGATCAAGTCCACAAAGCTGGAGGACTCAGCCATGTACTTCTGTGCCAGCAGTGAAGC
>99|TRBV2-1*02 IMGT000073|TRBV2-1|L-REGION+V-REGION|TR|TRB|None|02
ATGGATACCCGCCTCCTATGCTGGGCAATTTTTAGTCTCTTGAAAGCAGGACACACAGAACCTGAAGTCA
CCCAGACTCCCAGCCATCAGGTCACACAGATGGGACAGGAAGTGATCTTGTGGTGTGTCCCCATCCCTAA

TCACTTAAACTTCTATTGGTACAGACAAATCTTGGGGCAGAAAGTCGAGTTTCTGGTTATCTTTTTTGAT
AATAACATCTCAGAGAAGTCTGAAATATTTGATGATCGATTCTCAGTCGGAAGGCCTGATGGATCAAATT
TCACTCTGAAGATCAAGTCCACAAAGCTGGAGGACTCAGCCATGTACTTCTGTGCCAGCAGTGAAGC
>100|TRBV2-2*01 NW_001114291|TRBV2-2|L-REGION+V-REGION|TR|TRB|None|01
ATGGATACCTGGCTCCTATGTTGGGCAATTTTTAGTCTCTTGAAAGCAGGACACACAGAACCTGAAGTCA
CCCAGACTCCCAGCCATCAGGTCACACAGATGGGACAGGAAGTGATCTTGCGGTGTGTCCCCATCTCTAA
TCATTTCTACTTCTATTGGTACAGACAAATCTTGGGGCAGAAAGTCGAGTTTCTGGTTTCCTTCTATAAT
GGTAAGATCTCAGAGCAGTCTGAAATATTTGAAGATCAATTCTCAGTTGGAAGGTCTGATGGAGTACATT
TCACGCTGAAGATCAAGTCCACAAAGCTGGAGGACTCAGCCATGTACTTCTGTGCCAGCAGTGAAGC
>101|TRBV2-3*01 NW_001114291|TRBV2-3|L-REGION+V-REGION|TR|TRB|None|01
ATGGATACCTGGCTCCTATGCTGGGCAATTTTTAGTCTCTTGAAAGCAGGACACACAGAACCTGAAGTCA
CCCAGACTCCCAGCCATCAGGTCACACGGATGGGACAGGAAGTGATCTTGCGGTGTGTCCCCATCCCTAA
TCACTTATACTTCTATTGGTACAGACAAATCTTGGGGCAGAAAGTCGAGTTTCTGGTTTACTTCTATAAT
GGTGAGATCTCAGAGAAGTCTGAAATATTTGAAGATCGATTCTCAGTTGGAAGGTCTGATGGAGTACATT
TCACTCTGAAGATCAAGTCCACAAAGCTGGAGGACTCAGCCACGTACTTCTGTGCCAGCAGTGAAGC
>102|TRBV2-3*02 IMGT000073|TRBV2-3|L-REGION+V-REGION|TR|TRB|None|02
ATGGATACTTGGCTCCTATGCTGGGCAATTTTTAGTCTCTTGAAAGCAGGACACACAGAACCTGAAGTCA
CCCAGACTCCCAGCCATCAGGTCACACGGATGGGACAGGAAGTGATCTTGCGGTGTGTCCCCATCCCTAA
TCACTTATACTTCTATTGGTACAGACAAATCTTGGGGCAGAAAGTCGAGTTTCTGGTTTACTTCTATAAT
GATAAGATCTCAGACAAGTCTGAAATGTTCGATGATCGATTCTCAGTTGAAAGGCCTGATGGATCAAATT
TCACTCTGAAGATCCAGTCCACAAAGCTGGAGGACTCAGCCATGTACTTCTGTGCCAGCAGTGAAGC
>103|TRBV20-1*01 NW_001114291|TRBV20-1|L-REGION+V-REGION|TR|TRB|None|
01
ATGCTGTTGCTTCTGCTGCTTCTGGGGACAGGCTCCGGGCTTGCTGCTGTCGTCTCTCAGTATCCAAGCA
GGGTTATCTGTAAGAGAGGAACCTCTGTGAAGATCGAGTGCCGTTGCCTGGACTTTCAGGCCACAACTAT
GTTTTGGTATCGTCAGTTCCAGACACAGAGCCTCATACTCATGGCAACTTCCAATGAGGGCTCCGGCGTC
ACATACGAGCAAGGCGTCAAGCAGGACAAGTTTCCCATCAACCATCCAAACCTGACCTTCTCCACTCTGA
CAGTGACCAATGCCCATCCTGAAGACAGCAGCTTCTACATCTGCAGTGCTAGAGA
>104|TRBV20-1*02 IMGT000073|TRBV20-1|L-REGION+V-REGION|TR|TRB|None|02
ATGCTGTTGCTTCTGCTGCTTCTGGGGACAGGCTCCGGGCTTGCTGCTGTCGTCTCTCAATATCCAAGCA
GGGTTATCTGTAAGAGAGGAACCTCTGTGAAGATCGAGTGCCGTTGCCTGGACTTTCAGGCCACAACTAT
GTTTTGGTATCGTCAGTTCCAGACACAGAGCCTCATACTCATGGCAACTTCCAATGAGGGCTCCGGCGTC
ACATACGAGCAAGGCGTCAAGCAGGACAAGTTTCCCATCAACCATCCAAACCTGACCTTCTCCACTCTGA
CAGTGACCAATGCCCATCCTGAAGACAGCAGCTTCTACATCTGCAGTGCTAGAGA
>105|TRBV21-1*01 NW_001114291|TRBV21-1|L-REGION+V-REGION|TR|TRB|None|
01
ATGTGCCTCAGACTTCTCTGCTGTGTGGCCCTTTCTTTCTGGGGAGCAGCCTCCACGGACACCAAGGTCA
CCCAGAGACCTAGACTTCTGGTCAAAGCAAACAAACAGAAAGCAAAGATGGATTGTGTTCCTGTAAAAGG
ACATAGTTATGTTTACTGGTATCGTAAGAAGCTGGACGAAGAGCTCAAGTTTTTGGTTTACTTTCAGAAT
GAAGAAATTATTCAGAAAGCAGAAATAATCAATAAGAGATTTTCAGCCCAATGTCCCCAAAACTCATCCT
GTACCTTGGAGATCCAGTCCACAGAGTCAGGGGACGCAGCACTGTATTTCTGTGCCAGCAAAGC
>106|TRBV21-1*02 IMGT000073|TRBV21-1|L-REGION+V-REGION|TR|TRB|None|02
ATGTGCCTCAGACTTCTCTGCTGTGTGGCCCTTTCTTTCTGGGGAGCAGCCTCCACGGACACCAAGGTCA
CCCAGAGACCTAGACTTCTGGTCAAAGCAAACAAACAGAAAGCAAAGATGGATTGTGTTCCTGTAAAAGG
ACATAGTTATGTTTACTGGTATCGTAAGAAGCTGGACGAAGAGCTCAAGTTTTTGGTTTACTTTCAGAAT
GAAGAAATTATTCAGAAAGCAGAAATAATCAATAAGAGATTTTCAGCCCAATGTCCCCAAAACTCATCTT
GTACCTTGGAGATCCAGTCCACAGAGTCAGGGGACGCAGCACTGTATTTCTGTGCCAGCAGCAAAGC
>107|TRBV23-1*01 NW_001114291|TRBV23-1|L-REGION+V-REGION|TR|TRB|None|
01
ATGGGCACCAGGCTCCTCGGCTGTGCAGCCCTGTGTCTCCTGGCAGCAGGCTCTTTTCATGCCGAAGTCA

CACAGACTCCAGGATATTTGGTCAAAGGAAAAGGACAGAAAACAAAGATGGATTGTACCCCCGAAAAAGG
ACATACTTATGTTTATTGGTATCAACAGAATCAGAATAAAGAGTTTACATTTTTGATTTCCTTTCAGAAT
GAACAAGTTCTTCAAGAAACGGAGCTGCACAAGAAGCGGTTCTCATCTCAATGCCCCAAGAACCTACCTT
GCAGCCTGGAGATCCCGTCCTCGGAACCAGGAGACACCGCACTCTATCTCTGTGCCAGCAGCCAATC
>108|TRBV24−1*01 NW_001114291|TRBV24−1|L−REGION+V−REGION|TR|TRB|None|
01
ATGGCCTCCCTGCTCCTCTTCTGTGTGACCTTTTGTCTCCTGGGAACAGGGTCCATGGATGCTGATGTTA
CCCAGACCCCAAGGAATAAGATCGCAAAGACAGGAAAGAGGATTATGCTGGAATGTTCTCAGACTAAGGG
TCATGATCAAATGTACTGGTATCGACAAGACCCAGGACTGGGGCTACGGTTGATCTATTACTCCTTTGAT
GTCCAAGATATAAACAAAGGAGAGATCTCCAATGGATACAGTGTCTCTCGACAGGAACAGGCTAAATTCT
CCCTGTCCCTAGAGTCTGCCACCCCCAACCAGACAGCTCTTTACTTCTGTGCCACCAGTGATTTG
>109|TRBV25−1*01 NW_001114291|TRBV25−1|L−REGION+V−REGION|TR|TRB|None|
01
ATGACTATCAGGCTCCTCTGCCACATGGCCTTTTATTTTCTGGGGGCAGGCCTTACGGAAGCTGATATCT
ACCAGAACCCAAGACACCGTGTTATAGGGACAGGAAAGAAGATCACTCTGGAATGTTCTCAAACCATGGG
CCATGACAAAATGTACTGGTATCAACAAGATCCAGGAATGGAATTACACCTCATCCACTATTCCTATGGG
GTTAATTCCACAGAGAGGGGAGATTTTTCCTCTGAGTCAACAGTCTCCAGACTAAGGATAGAGCATTTTC
CCCTGACCCTGGAGTCTGCCAGCCCCTCACACACCTCTCAGTACCTCTGTGCCAGCAGTGAATA
>110|TRBV27*01 NW_001114291|TRBV27|L−REGION+V−REGION|TR|TRB|None|01
ATGGGCCCCCAGCTCCTTGGCTATGTGGTCCTTTGCCTTCTAGGAGCAGGCCCCTTGGAAGCCCAAGTGA
CCCAGAACCCAAGATACCTCATCACAGTGACTGGAAAGAAGTTGACAGTCACTTGTTCTCAGAATATGAA
CCATGAGTATATGTCCTGGTATCGACAAGACCCAGGACTGGGCCTGAGGCAGATCTACTATTCAGTTAAT
GTTGAGATGGTTGATAAGGGAGATATTCCTGAAGGGTACAACGTCTCTCGAAAAGAGAAGAGGAATTTCC
CCCTGATCCTGGAGTCGCCCAGCCCCAGCCAGACCTCTCTGTACCTCTGTGCCAGCAGTTTATC
>111|TRBV27*02 IMGT000073|TRBV27|L−REGION+V−REGION|TR|TRB|None|02
ATGGGCCCCCAGCTCCTTGGCTATGTGGTCCTTTGCCTTCTAGGAGCAGGCCCCTTGGAAGCCCAAGTGA
CCCAGAACCCAAGATACCTCATCACAGTGACTGGAAAGAAGTTGACAGTCACTTGTTCTCAGAATATGAA
CCATGATTATATGTCCTGGTATCGACAAGACCCAGGACTGGGCCTGAGGCAGATCTACTATTCAGTTAAT
GTTGAGATGGTTGATAAGGGAGATATTCCTGAAGGGTACAACGTCTCTCGAAAAGAGAAGAGGAATTTCC
CCCTGATCCTGGAGTCGCCCAGCCCCAGCCAGACCTCTCTGTACCTCTGTGCCAGCAGTTTATC
>112|TRBV28*01 NW_001114291|TRBV28|L−REGION+V−REGION|TR|TRB|None|01
ATGGGAACCAGGCTCCTCTGTTATGTGGCCTTTTGTTTCCTGGCTGTAGGCCTCGTGGATGTGAAAGTAA
CCCAGAGCTCAAGATATCTAATCAAAAGGACAGGAGAGAAAGTTTTTCTGGAATGTGTCCAGGATATGGA
CCATGAAAGAATGTTCTGGTATCGACAAGACCCAGGTCTGGGGCTACGGCTGATCTATTTCTCATATGAT
GTTAAACTGAACGAAAAAGGAGATATTCCTGAGGGGTACAGTGTCTCTAGAGAGAAGAAGGAGCGCTTCT
CCCTGATTCTGGGGTCCGCCAGCACCAACCAGACATCTATGTACCTCTGTGCCAGCAGTTTATC
>113|TRBV29−1*01 NW_001114291|TRBV29−1|L−REGION+V−REGION|TR|TRB|None|
01
ATGCTGGGTCTCCTGCTCCTTCTCCTGGGACGAGGCTCTGTGTTCAGTGCTGTCATCTCTCAAAAGCCAA
GCAGGGATGTCTGTCAACGTGGAACCTCCGTGAAGATCCAGTGTCAAGTCGATAGCCAAGTCACCATGAT
GTTCTGGTACCGTCAGCAACCTGGACAGAGCATGACACTGATTGCAACTGCAAATCAGGGCTCTGAGGCC
ACATATGAGAGTGGATTTGTCATTGACAAGTTTCCCATCAGTCGCCCAAACCTAACATTCTCAACTCTAA
CTGTGAGCAACACGAGCCCTGAAGACAGCAGCATATACCTCTGCAGCGTTGAAGA
>114|TRBV29−1*02 IMGT000073|TRBV29−1|L−REGION+V−REGION|TR|TRB|None|02
ATGCTGGGTCTCCTGCTCCTTCTCCTGGGACGAGGCTCAGTGTTCAGTGCTGTCGTCTCTCAAAAGCCAA
GCAGGGATGTCTGTCAACGTGGAACCTCCGTGACGATCCAGTGTCAAGTCGATAGCCAAGTCACCATGAT
GTTCTGGTACCGTCAGCAACCTGGACAGAGCATGACACTGATTGCAACTGCAAATCAGGGCTCCGAGGCC
ACATATGAGAGTGGATTTGTCATTGACAAGTTTCCCATCAGTCGCCCAAACCTAACATTCTCAACTCTGA
CTGTGAGCAACACGAGCCCTGAAGACAGCAGCATATACCTCTGCAGCGTTGAAGA
>115|TRBV3−1*01   NW_001114291|TRBV3−1|L−REGION+V−REGION|TR|TRB|None|01

ATGAGCTGCAGGCTCCTCTGCTGTGTGGCCCTCTGCCTCCTCCAAGCAGGTTCCTTGGACACAGCTGTTT
CCCAGACTCCAAAATACCTGGTCACACAGACAGGAAAGAACGAGTACCTTAAATGTGAACAAAATCTGGG
CCATGATACTATGTATTGGTATAAGCAGGACTCTAAGAAATTGCTGAAGATAATGTTTAGCTACAATAAC
AAGCAGCTCATTATAAATGAAACAGTTCCAAATCGCATCTCACCTGACTCTCCAGACAAAGCTCATTTAA
ATCTTCACATCAAGTCCCTGGAGCTTGGTGACTCTGCTGTATATTTCTGTGCCAGCAGCCAAGG
>116|TRBV3-2*01 NW_001114291|TRBV3-2|L-REGION+V-REGION|TR|TRB|None|01
ATGGGCTGCAGGCTCCTCTGCTGTGTGACCCTCTGCCTCCTCCAAGCAGGTTCCTTGGACACAGCTGTTT
CCCAGACTCCAAAATACCTGGTCACACAGACAGGAAAGGAGGAGTACCTTAAATGTGAACAAAATCTGGG
TCATGATACTATGTATTGGTATAAGCAGGACTCTAAGAAATTGCTGAAGATAATGTTTAGCTACAATAAC
AAGAAGCTCTTTATAAATGAAACAGTTTCAAATCGCTTCTCACCTGACTCTCCAGACAAAGCTCGTTTAA
TTCTTCACATCAAGCCCCTGGAGCTTGGTGACTCTGCTGTGTATTTCTGTGCCAGCAGCCAAGG
>117|TRBV3-2*02 IMGT000073|TRBV3-2|L-REGION+V-REGION|TR|TRB|None|02
ATGGGCTGCAGGCTCCTCTGCTGTGTGACCCTCTGCCTCCTCCAAGCAGGTTCCTTGGACACAGCTGTTT
CCCAGACTCCAAAATACCTGGTCACACAGACAGGAAAGGAGGAGTCCCTTAAATGTGAACAAAATCTGGG
TCATGATACTATGTATTGGTATAAGCAGGACTCTAAGAAATTGCTGAAGATAATGTTTAGCTACAATAAC
AAGAAGCTCTTTATAAATGAAACAGTTTCAAATCGCTTCTCACCTGACTCTCCAGACAAAGCTCGTTTAA
TTCTTCACATCAAGCCCCTGGAGCTTGGTGACTCTGCTGTATATTTCTGTGCCAGCAGCCAAGG
>118|TRBV3-3*01 NW_001114291|TRBV3-3|L-REGION+V-REGION|TR|TRB|None|01
ATGGGCTGCAGGCTCCTCTGCTGTGTGGTCCTCTGCCTCCTCCAAGCAGGTCCCTTGGACACAGCTGTTT
TCCAGACTCCAAAATACCTGGTCACACAGATGGGAAAGAAGGAGTCCCTTAAATGTGAACAAAATCTGGG
TCATGATACTATGTATTGGTATAAGCAGGACTCCAAGAAATTGCTGAAGATAATGTTTATCTACAGTAAT
AAGGAGCCCATTTTAAATGAAACAGTTCCAAATCGCTTCTCACCTGAATCTCCAGACAAAGCTCATTTAA
ATCTTCACATCAAGTCTGTGGAGCTTGGTGACTCTGCTGTGTATTTCTGTGCCAGCAGCCAAGG
>119|TRBV3-3*02 IMGT000073|TRBV3-3|L-REGION+V-REGION|TR|TRB|None|02
ATGGGCTGCAGGCTCCTCTGCTGTGTGGTCCTCTGCCTCCTCCAAGCAGGTCCCTTGGACACAGCTGTTT
TCCAGACTCCAAAATACCTGGTCACACAGATGGGAAAGAAGGAGTCCCTTAAATGTGAACAAAATCTGGG
TCATGATACTATGTATTGGTATAAGCAGGACTCCAAGAAATTGCTGAAGATAATGTTTATCTACAGTAAT
AAGGAGCCCATTTTAAATGAAACAGTTCCAAATCGCTTCTCACCTGAATCTCTAGACAAAGCTCATTTAA
ATCTTCACATCAAGTCTGTGGAGCTCGGTGACTCTGCTGTGTATTTCTGTGCCAGCAGCCAAGG
>120|TRBV3-4*01 NW_001114291|TRBV3-4|L-REGION+V-REGION|TR|TRB|None|01
ATGGGCTGCAGGCTCCTCTGCTGTGTGGCCCTCTGCCTCCTCCAAGCAGGTTCCTTGGACACAGCTGTTT
CCCAGACTCCAAAATATCTGGTCAGGCAGACGGGAAAAAACGAGTCCCTTAAATGTGAACAAAATCTGGG
CCATGATGCTATGTATTGGTATAAGCAGGACTCTAAGAAATTGCTGAAGATAATGTTTATCTACAATAAT
AAGGAGCCCATTTTAAATGAAACAGTTCCATATCGCTTCTCACCTAAGTCTCCAGACAAAGCTCATTTAA
ATCTTCACATCAAGTCCCTGGAGCTTGGTGACTCTGCTGTATATTTCTGTGCCAGCAGCCAAGA
>121|TRBV3-4*02 IMGT000073|TRBV3-4|L-REGION+V-REGION|TR|TRB|None|02
ATGGGCTGCAGGCTCCTCTGCTGTGTGGCCCTCTGCCTCCTCCAAGCAGGTTCCTTGGACACAGCTGTTT
CCCAGACTCCAAAATATCTGGTCAGGCAGACGGGAAAAAACGAGTCCCTTAAATGTGAACAAAATCTGGG
CCATAATGCTATGTATTGGTATAAGCAGGACTCTAAGAAATTGCTGAAGATAATGTTTATCTACAATAAT
AAGGAGCCCATTTTAAATGAAACAGTTCCATATCGCTTCTCACCTAAGTCTCCAGACAAAGCTCATTTAA
ATCTTCACATCAAGTCCCTGGAGCTTGGTGACTCTGCTGTATATTTCTGTGCCAGCAGCCAAGA
>122|TRBV30*01 NW_001114291|TRBV30|L-REGION+V-REGION|TR|TRB|None|01
ATGCTCTGCTCTCCCCTTGCCCTTCTCCTGGGCACTTTCTTCGGGGTCAGATCTCAGACTGTTCATCAAT
GGCCAGCGACCCTGGTGCAGCCTGCGGGCAGCCCGCTTTCTCTGGAGTGCACTGTGGAGGGAACATCAAA
CCCCAACCTATACTGGTACCGACAGGCTGCAGGCAGGGGCCTCCAGCTGCTCTTCTACTCCATTGGTGTT
GACCAGATCAGCTCTGAGGTGCCCCAGAATCTCTCAGCCTCCAGGCCCCAGGACAGGCGGTTCATCCTGA
GTTCTAAGAAGCTCCTCCTCAGTGACTCAGGCTTCTATCTCTGTGCCTGGAGTGT
>123|TRBV4-1*01 NW_001114291|TRBV4-1|L-REGION+V-REGION|TR|TRB|None|01
ATGGGCTGCAGGCTGCTCTGCTGTGTGGTTCTCTGTCTCCTGGGAGCAGTCCCCATAGACAGTGGAGTTA
CCCAAACACCAAAGCACCTGGTCATGGGAATGACAAACAAGAAGTCTTTGAAATGTGAACAACATATGGG

ACACAGGGCTGTGTACTGGTACAAGCAGAAAGTTAAGAAGCCGCCGGAGATCATGTTTATCTACAACTAT
GAGAAACTTTCTATTAATGAAAGTGTGCCCAGTCGCTTCTCACCTGAATGCTCCAAGAGCTCTCTCTTAT
ACCTTCACCTACGCGCCCTGCAGCCAGAAGACTCAGCCCTGTATCTCTGCGCCAGCAGCCAAGA
>124|TRBV4-1*02 IMGT000073|TRBV4-1|L-REGION+V-REGION|TR|TRB|None|02
ATGGGCTGCAGGCTGCTCTGCTGTGTGGTTCTCTGTCTCCTGGGAGCAGTCCCCATAGACAGTGGAGTTA
CCCAAACACCAAAGCACCTGGTCATGGGAATGACAAACAAGAAGTCTTTGAAATGTGAACAACATATGGG
ACACAGGGCTGTGTACTGGTACAAGCAGAAAGTTAAGAAGCCGCCGGAGATCATGTTTATGTACAACTAT
GAGAAACTTTCTATTAATGAAAGTGTGCCCAGTCGCTTCTCACCTGAATGCTCCAAGAGCTCTCTCTTCT
ACCTTCACCTACGCGCCCTGCAGCCAGAAGACTCAGCCCTGTATCTCTGCGCCAGCAGCCAAGA
>125|TRBV4-2*01 NW_001114291|TRBV4-2|L-REGION+V-REGION|TR|TRB|None|01
ATGGGCTGCAGGCTGCTCTGCTGTGCGGTTCTCTGTCTCCTGGGAGCAGTCCCCACAGACACTGGAGTTA
CCCAGACACCAAAGCACCTGGTCATGGGAATGACAAATAAGAAGTCTTTGAAATGTGAACAACATATGGG
ACACAATGCTATGTACTGGTACAAGCAGAAAGCTAAGAAGCCGCCGGAGCTCATGTTTGTCTACCAGTAT
GAGAAACTCTCTATTAATGAAAGTGTGCCCAGTCGCTTCTCACCTGAATGCTCCAAGAGCTCTCTCTTAT
ACCTTCACCTACGCGCCCTGCAGCCAGAAGACTCAGCCCTGTATCTCTGCGCCAGCAGCCAAGA
>126|TRBV4-2*02 IMGT000073|TRBV4-2|L-REGION+V-REGION|TR|TRB|None|02
ATGGGCTGCAGGCTGCTCTGCTGTGCGGTTCTCTGTCTCCTGGGAGCAGTCCCCACAGACACTGGAGTTA
CCCAGACACCAAAGCACCTGGTCATGGGAATGACAAATAAGAAGTCTTTGAAATGTGAACAACATATGGG
ACACAATGCTATGTACTGGTACAAGCAGAAAGCTAAGAAGCCGCCGGAGATCATGTTTGTCTACCAGTAT
GAGAAACTCTCTATTAATGAAAGTGTGCCCAGTCGCTTCTCACCTGAATGCTCCAAGAGCTCTCTCTTAT
ACCTTCACCTACGCGCCCTGCAGCCAGAAGACTCAGCCCTGTATCTCTGCGCCAGCAGCCAAGA
>127|TRBV4-3*01 NW_001114291|TRBV4-3|L-REGION+V-REGION|TR|TRB|None|01
ATGGGCTGCAGGCTGCTCTGCTGTGCGGTTCTCTGTCTCCTGGGAGCAGTCCCCATGGAAACTGGAGTTA
CGCAGACACCAAGACACCTGGTCATGGGAATGACAAATAAGAAGTCTTTGAAATGTGAACAACATCTGGG
GCACAATGCTATGTACTGGTACAAGCAGAAAGCTAAGAAGCCACCGGAGCTCATGTTTGTCTACAACTTT
AAAGAACGGGCTGAAAACAACAGTGTGCCCAGTCGCTTCGTACCTGAATGCCCAGACAGCTCTCACTTAC
ACCTTCACCTACACGCCCTGCAGCCAGAAGACTCAGCCCTGTATCTCTGCGCCAGCAGCCAAGA
>128|TRBV4-3*02 IMGT000012|TRBV4-3|L-REGION+V-REGION|TR|TRB|None|02
ATGGGCTGCAGGCTGCTCTGCTGTGCGGTTCTCTGTCTCCTGGGAGCAGTCCCCATGGAAACTGGAGTTA
CGCAGACACCAAGACACCTGGTCATGGGAATGACAAATAAGAAGTCTTTGAAATGTGAACAACATCTGGG
GCACAATGCTATGTACTGGTACAAGCAGAAAGCTAAGAAGCCACCGGAGCTCATGTTTATCTACAACTTT
AAAGAACGGGCTGAAAACAACAGTGTGCCCAGTCGCTTCTCACCTGAATGCCCAGACAGCTCTCACTTAT
ACCTTCACCTACGCGCCCTGCAGCCAGAAGACTCAGCCCTGTATCTCTGCGCCAGCAGCCAAGA
>129|TRBV4-3*03 IMGT000073|TRBV4-3|L-REGION+V-REGION|TR|TRB|None|03
ATGGGCTGCAGGCTGCTCTGCTGTGCGGTTCTCTGTCTCCTGGGAGCAGTCCCCATGGAAACTGGAGTTA
CGCAGACACCAAGACACCTGGTCATGGGAATGACAAATAAGAAGTCTTTGAAATGTGAACAACATCTGGG
GCACAATGCTATGTACTGGTACAAGCAGAAAGCTAAGAAGCCACCGGAGCTCATGTTTGTCTACAACTTT
AAAGAACGGGCTGAAAACAACAGTGTGCCCAGTCGCTTCTCACCTGAATGCCCAGACAGCTCTCACTTAC
ACCTTCACCTACGCGCCCTGCAGCCAGAAGACTCAGCCCTGTATCTCTGCGCCAGCAGCCAAGA
>130|TRBV5-10*01 NW_001114291|TRBV5-10|L-REGION+V-REGION|TR|TRB|None|
01
ATGGGCCCCGGGCTCCTCTGCTGGGCGCTGCTCTGTCTCCTGGGAGCAGGCCCAGTGGACGCTGGAGTCG
TCCAAAGTCCCACGCACCTGATCAAAACGAGAGGACAGCAAGTGACTCTGAGATGCTCTCCTATCTCTGG
GCACAACACTGTGTCCTGGTACCAACAGGCACTCGGTCAGGGGCCCCAGTTGATATTTCAGTATTATCGG
GGGGAAGACAGAGGCAGAGGAAACTTCCCTGATCGATTCTCAGGTCACCAGTTCCCTAACTATAGCTCTG
AGCTGAATGTGAATGCCTCGGAGATGGGGGACTCGGCCCTGTATCTCTGTGCCAGCAGCTTGG
>131|TRBV5-10*02 IMGT000073|TRBV5-10|L-REGION+V-REGION|TR|TRB|None|02
ATGGGCCCCGGGCTCCTCTGCTGGGCGCTGCTCTGTCTCCTGGGAGCAGGCCCAGTGGACGCTGGAGTCG
TCCAAAGTCCCACGCACCTGATCAAAACGAGAGGACAGCAAGGGACTCTGAGATGCTCTCCTATCTCTGG
GCACAACACTGTGTCCTGGTACCAACAGGCACTCGGTCAGGGGCCCCAGTTGATATTTCAGTATTATCGG

```
GGGGAAGACAGAGGCAGAGGAAACTTCCCTGATCGATTCTCAGGTCACCAGTTCCCTAACTATAGCTCTG
AGCTGAATGTGAATGCCTCGGAGATGGGGGACTCGGCCCTGTATCTCTGTGCCAGCAGCTTGG
>132|TRBV5-3*01 NW_001114291|TRBV5-3|L-REGION+V-REGION|TR|TRB|None|01
ATGGGCCCCGGGCTCCTCTGCTGGGCGCTGCTCTGTCTCCTGGGAGCAGGCTCAGTGGACACTGGAGTCA
CCCAAAGTCCCACACACCTGATCAAAACGAGAGGACAGCAAGTGACTCTGAGATGGTCTCCTATCTCTGG
GCACAACACTGTGTCCTGGTACCAACAGGCCCCTGGTCAGGGGCCCCAGTTTATCTTCGAATATGCTAAT
GAGTCAAGGACATCAGAAGGAAACTTCCCTCATCGATTCTCAGGGCGCCAGTTCCGTGACTATCATCACT
CTGAGATGAATGTGAGTGCCTTGGAGCTGGGGGACTCGGCCCTGTATCTCTGTGCCAGCAGCTTGG
>133|TRBV5-3*02 IMGT000012|TRBV5-3|L-REGION+V-REGION|TR|TRB|None|02
ATGGGCCCCGGGCTCCTCTGCTGGGCGCTGCTCTGTCTCCTGGGAGCAGGCTCAGTGGACACTGGAGTCA
CCCAAAGTCCCACACACCTGATCAAAACGAGAGGACAGCAAGTGACTCTGAGATGCTCTCCTATCTCTGG
GCACAACACTGTGTCCTGGTACCAACAGGCCCCTGGTCAGGGGCCCCAGTTTATCTTCGAATATGCTAAT
GAGTCAAGGACATCAGAAGGAAACTTCCCTCATCGATTCTCAGGGCGCCAGTTCCGTGACTATCATCACT
CTGAGATGAATGTGAGTGCCTTGGAGCTGGGGGACTCGGCCCTGTATCTCTGTGCCAGCAGCTTGG
>134|TRBV5-3*03 IMGT000073|TRBV5-3|L-REGION+V-REGION|TR|TRB|None|03
ATGGGCCCCGGGCTCCTCTCCTGGGCGCTGCTTTGTCTCCTGGGAGCAGGCTCAGTGGACACTGGAGTCA
CCCAAAGTCCCACACACCTGATCAAAACGAGAGGACAGCAAGTGACTCTGAGATGCTCTCCTATCTCTGG
GCACAGCACTGTGTCCTGGTACCAACAGGCCCCTGGTCAGGGGCCCCAGTTTATCTTCGAATATGCTAAT
GAGTTAAGGACATCAGAAGGAAACTTCCCTCATCGATTCTCAGGGCGCCAGTTCTGTGACTATCATCACT
CTGAGCTGAATGTGAGCGCCTTGGAGCTGGGGGACTCGGCCCTGTATCTCTGTGCCAGCAGCTTGG
>135|TRBV5-4*01 NW_001114291|TRBV5-4|L-REGION+V-REGION|TR|TRB|None|01
ATGGGCCCCGGGCTCCTCTGCTGGGCGCTGCTCTGTCTCCTGGGAGCAGGCCCAGTGCAGGCTGGAGTCA
CCCAAAGTCCCACACACCTGATCAAAACGAGAGGACAGCAAGTGACTCTGAGATGCTCTCCTATCTCTGG
GCACACCAATGTGTACTGGTACCAACAGGCCCTGGGTCAGGGACTCCAGTTCATCCTTTGGTATGACGAG
GAAGAAGAGAGAGACAGAGGAACCTTCCCTCCTAGATTTTCAGGTCATCAGTTCCCTAACTATAGCTCTG
AGCTGAATGTGAACGCCTTGGAGGTGGAGGACTCGGCCCTGTATCTCTGTGCCAGCAGCTTGG
>136|TRBV5-4*02 IMGT000012|TRBV5-4|L-REGION+V-REGION|TR|TRB|None|02
ATGGGCCCCGGGCTCCTCTGCTGGGCGCTGCTCTGTCTCCTGGGAGCAGGCCCAGTGCAGGCTGGAGTCA
CCCAAAGTCCCACACACCTGATCAAAACGAGAGGACAGCAAGTGACTCTGAGATGCTCTCCTATCTCTGG
GCACACCAATGTGTACTGGTACCAACAGGCCCTGGGTCAGGGACTCCAGTTCATCCTTTGATATGACGAG
GAAGAAGAGAGAGACAGAGGAACCTTCCCTCCTAGATTTTCAGGTCATCAGTTCCCTAACTATAGCTCTG
AGCTGAATGTGAACGCCTTGGAGGTGGAGGACTCGGCCCTGTATCTCTGTGCCAGCAGCTTGG
>137|TRBV5-4*03 IMGT000073|TRBV5-4|L-REGION+V-REGION|TR|TRB|None|03
ATGGGCCCCGGGCTCCTCTGCTGGGCGCTGCTCTGTCTCCTGGGAGCAGGCCCAGTGCAGGCTGGAGTCA
CCCAAAGTCCCACACACCTGATCAAAACGAGAGGACAGCAAGTGACTCTGAGATGCTCTCCTATCTCTGG
GCACACCAATGTGTACTGGTACCAACAGGCCCTGGGTCAGGTACTCCAGTTCATCCTTTGGTATGACGAG
GAAGAAGAGAGAGACAGAGGAACCTTCCCTCATAGATTTTCAGGTCATCAGTTCCCTAACTATAGCTCTG
AGCTGAATGTGAACGCCTTGGAGGTGGAGGACTCGGCCCTGTATCTCTGTGCCAGCAGCCTGG
>138|TRBV5-5*02 IMGT000012|TRBV5-5|L-REGION+V-REGION|TR|TRB|None|02
ATGGGCCCCGGGCTCCTCTGCTGGGCGCTGCTTTGTCTACTGGGAGCAGGCTCAGTGGACGGTGGAGTCA
CCCAAAGTCCCAGGCATTTGATCAAAACAACAGGACAGCAAGTGACACTGAGATGCTCTCCTATCTCTGG
GCACACCAGTGTGTCCTGGTACCAACAGGCCCTGGGTCAGGGGCCCCAGCTTATCTTTGAGTATTATGAG
GAGAAAGAGAGAGAGAGAGAGGCAACTTCCCTGATCGATTCTCAGGTCACCAGTTCCCTAACTATAGCTCTG
AGCTGAACGTGAATGCCTTGGAGGCGGGGGACTCGGCCCTGTATCTCTGTGCCAGCAGCTTGG
>139|TRBV5-5*03 IMGT000073|TRBV5-5|L-REGION+V-REGION|TR|TRB|None|03
ATGGGCCCCGGGCTCCTCTGCTGGGCGCTGCTTTGTCTCCTGGGAGCAGGCTCAGTGGACGGTGGAGTCA
CCCAAAGTCCCAGGCATTTGATCAAAACAACAGGACAGCAAGTGACACTGAGATGCTCTCCTATCTCTGG
GCACAACAGTGTGTCCTGGTACCAACAGGCCCTGGGTCAGGGGCCCCAGTTTATCTTTGAGTATTATGAG
GAGAAAGAGAGAGAGAGAGAGGCAACTTCCCTGATCGATTCTCAGGTCACCAGTTCCCTAACTATAGCTCTG
AGCTGAACGTGAATGCCTTGGAGGCGGGGGACTCGGCCCTGTATCTCTGTGCCAGCAGCTTGG
```

>140|TRBV5-6*01 NW_001114291|TRBV5-6|L-REGION+V-REGION|TR|TRB|None|01
ATGGGCCCCGGGCTCCTCTGCTGGGCGCTGCTTTGTCTCCTGGGAGCAGGCTCAGTGGACACTGGAGTCA
CCCAAAGTCCCACACACCTGATCAGAAAGAGAGGACAGCAAGTGACACTGAGATGCTCTCCTATCTCTGG
GCACAACACTGTGTCCTGGTACCAACAGGCCCTGGGTCAGGGGCCCCAGTTTATCTTTCAGTATTATGAG
AAGGAAGAGAGAGAGAGAGAGGCAACTTTCCTGGTCGATTCTCAGGTCACCAGTTCCCTAACTATAGCTCTG
AGCTGAATGTGAGCGCCTTGTTGCTGGGGGACTCAGCCCTGTATCTCTGTGCCAGCAGCTTGG
>141|TRBV5-6*02 IMGT000073|TRBV5-6|L-REGION+V-REGION|TR|TRB|None|02
ATGGGCCCCGGGCTCCTCTGCTGGGCGCTGCTTTGTCTCCTGGGAGCAGGCTCAGTGGACGCTGGAGTCA
CCCAAAGTCCCACACACCTGATCAGAAAGAGAGGACAGCAAGTGACTCTGAGATGCTCTCCTATCTCTGG
GCACAACACTGTGTCCTGGTACCAACAGGCCCTGGGTCAGGGGCCCCAGTTTATCTTTCAGTATTATGAG
GAGGAAGAGAGAGAGAGAGAGGCAACTTCCCTGGTCGATTCTCAGGTCACCAGTTCCCTAACTATAGCTCTG
AGCTGAATGTGAGCGCCTTGTTGCTGGGGGACTCAGCCCTGTATCTCTGTGCCAGCAGCTTGG
>142|TRBV5-7*01 NW_001114291|TRBV5-7|L-REGION+V-REGION|TR|TRB|None|01
ATGGGCCCCGGGCTCCTCTGCTGGGCGCTGCTTTGTCTCCTGGGAGCAGGCCCAGTGGACGCTGGAGTCA
CCCAAAGTCCCACACACCTGATCAAAACGAGAGGACAGCAAGTGACTTTGAGATGCTCTCCTATCTCTGG
GCACAGCAGTGTGTCCTGGTACCAACAGGCCCTGGGTCAGGGGCCCCAGCTTATCTTTGAGTATTATGAG
AAGGAAGAGAGAGTCAGAGGCAACTTCCCTGATCGATTCTCAGGTCGCCAGTTCCCTAACTATAGCTCTG
AGCTGAATGTGAACGCCTCGGAGATGGGGGACTCGGCCCTGTATCTCTGTGCCAGCAGCTTGG
>143|TRBV5-8*01 NW_001114291|TRBV5-8|L-REGION+V-REGION|TR|TRB|None|01
ATGGGCCCCGGGCTCCTCTGCTGGGCGCTGCTTTGTCTCCTGGGAGCAGGCCCAGTGGACACTGGAGTCA
CCCAAAGTCCCACACACCTGATTAAAACAAGAGGACAGCAAGTGACTCTGAGATGCTCTCCTATCTCTGG
GCACAGCAGTGTGTCCTGGTACCAACAGGCCCTGGGTCAGGGGCCCCAGTTTATCTTTGAGTATTATGAG
AAGGAAGAGAGAGAGAGAGAGGCAACTTCCCTGGTCGATTCTCAGGTCACCAGTTCCCTAACTATAGCTCTG
AGCTGAATGTGAGCGCCTTGTTGCTGGGGGACTCGGCCTTGTATCTCTGCACCAGCAGCTTGG
>144|TRBV5-8*02 IMGT000073|TRBV5-8|L-REGION+V-REGION|TR|TRB|None|02
ATGGGCCCCGGGCTCCTCTGCTGGGCGCTGCTTTGTCTCCTGGGAGCAGGCCCAGTGGACACTGGAGTCA
CCCAAAGTCCCACACACCTGATTAAAACAAGAGGACAGCAAGTGATTCTGAGATGCTCTCCTATCTCTGG
GCACAGCAGTGTGTCCTGGTACCAACAGGCCCTGGGTCAGGGGCCCCAGTTTATCTTTGAGTATTATGAG
AAGGAAGAGAGAGAGAGAGAGGCAACTTCCCTGGTCGATTCTCAGGTCACCAGTTCCCTAACTATAGCTCTG
AGCTGAATGTGAGCGCCTTGTTGCTGGGGGACTCGGCCTTGTATCTCTGCACCAGCAGCTTGG
>145|TRBV5-9*01 NW_001114291|TRBV5-9|L-REGION+V-REGION|TR|TRB|None|01
ATGGGCCCCGGGCTCCTCTGCTGGGCGCTGCTTTGTCTCCTGGGAGCAGGCCCAGTGGACACTGGAGTCA
CCCAAAGTCCCACACACCTGATCAAAACGAGAGGACAGCAAGTGACACTGAGATGCTCTCCTATCTCTGG
GCACAGCAATGTGTACTGGTACCAACAGGCCCTGGGTCAGGGGCCCCAGTTTATCTTTCAGTATTATGAG
AAGGAAGAGAGAGAGAGAGAGGCAACTTCCCTGATCGATTCTCAGGTCACCAGTTCCCTAACTATAGCTCTG
AGCTGAATGTGAGCGCCTTGTTGCTGGGGGACTCGGCCCTGTATCTCTGTGCCAGCAGCTTGG
>146|TRBV5-9*02 IMGT000012|TRBV5-9|L-REGION+V-REGION|TR|TRB|None|02
ATGGGCCCCGGGCTCCTCTGCTGGGCGCTGCTTTGTCTCCTGGGAGCAGGCCCAGTGGACACTGGAGTCA
CCCAAAGTCCCACACACCTGATCAAAACGAGAGGACAGCAAGTGACACTGAGATGCTCTCCTATCTCTGG
GCACAGCAATGTGTCCTGGTACCAACAGGCCCTGGGTCAGGGGCCCCAGTTTATCTTTCAGTATTATGAG
AAGGAAGAGAGAGAGAGAGAGGCAACTTCCCTGATCGATTCTCAGGTCACCAGTTCCCTAACTATAGCTCTG
AGCTGAATGTGAGCGCCTTGTTGCTGGGGGACTCGGCCCTGTATCTCTGTGCCAGCAGCTTGG
>147|TRBV5-9*03 IMGT000073|TRBV5-9|L-REGION+V-REGION|TR|TRB|None|03
ATGGGCCCCGGGCTCCTCTGCTGGGCGCTGCTTTGTCTCCTGGGAGCAGGCCCAGTGGACACTGGAGTCA
CCCAAAGTCCCACACACCTGATCAAAACGAGAGGACAGCAAGTGACACTGAGATGCTCTCCTATCTCTGG
GCACAGCAGTGTGTCCTGGTACCAACAGGCCCTGGGTCAGGGGCCCCAGTTTATCTTTCAGTATTATGAG
AAGGAAGAGAGAGAGAGAGAGGCAACTTCCCTGATCGATTCTCAGGTCACCAGTTCCCTAACTATAGCTCTG
AGCTGAATGTGAGCGCCTTGTTGCTGGGGGACTCGGCCCTGTATCTCTGTGCCAGCAGCTTGG
>148|TRBV6-1*01 NW_001114291|TRBV6-1|L-REGION+V-REGION|TR|TRB|None|01
ATGAGCATCGGCCTCCTGTGCTGTGCGGCCTTTTCTCTCTTGTGGGCAGGTCCTGCAAATGCTGATGTCA

CTCAGAGCCCAAAATTCCAGATCCTGAAGACAGGACAGAGCATGACACTGCAGTGTGCCCAGGATATGAA
CCATAACTCCATGTACTGGTATCGACAAGACCCAGGCATGGGGCTGAGGCTGATTCATTACTCAGCTGCT
GAGGGTACCACTGACCCAGGAGAAGTCGCCGATGGCTACAGTGTCTCCAGATTAAACAAAACGGAGTTCC
TGCTCAGGCTGGAGTCGGCTGCTCCCTCCCAGACATCTGTGTACTTCTGTGCCAGCAGTGAAGC
>149|TRBV6-1*02 IMGT000012|TRBV6-1|L-REGION+V-REGION|TR|TRB|None|02
ATGAGCATCGGCCTCCTGTGCTGTGCGGCCTTTTCTCTCTTGTGGGCAGGTCCTGCAAATGCTGATGTCA
CTCAGAGCCCAAAATTCCAGATCCTGAAGACAGGACAGAGCATGACACTGCAGTGTGCCCAGGATATGAA
CCATAACTCCATGTACTGGTATCGACAAGACCCAGGCATGGGGCTGAGGCTGATTCATTACTCAGCTGCT
GAGGGTACCACTGACCCAGGAGAAGTCCCCGATGGCTACAGTGTCTCCAGATTAAACAAAACGGAGTTCC
TGCTCAGGCTGGAGTCGGCTGCTCCCTCCCAGACATCTGTGTACTTCTGTGCCAGCAGTGAAGC
>150|TRBV6-2*02 IMGT000012|TRBV6-2|L-REGION+V-REGION|TR|TRB|None|02
ATGAGCATCAGGCTCCTGTGCTGTGCGGCCTTTTCTCTCCTGTGGCCAGGTCCAGTGAATGCTGGTGTCA
CTCAGACCCCAAAATTCCAGGTCCTGAAGACAGGACAGAACATGACACTGAAGTGTGCCCAGGATATGAA
CCATAACCGTATGTACTGGTATCGACAAGACCCAGGCATGGGACTGAGGCTGATTCATTACTCAGCTGGT
GAAGGTAGCACTGAGAAAGGAGAAGTCCCCAATGGCTACAATGCCTCCAGATTAAACAAAAAGGATTTCC
TGCTCAGGCTGGAGTCAGCTGCTCCCTCCCAGACATCTATGTACTTCTGTGCCAGCAGTGAAGC
>151|TRBV6-2*03 IMGT000073|TRBV6-2|L-REGION+V-REGION|TR|TRB|None|03
ATGAACATCAGGCTCCTGTGCTGTGCGGCCTTTTCTCTCCTGTGGCCAGGTCCAGTGAATGCTGGTGTCA
CTCAGACCCCAAAATTCCAGGTCCTGAAGACAGGACAGAACATGACACTGAAGTGTGCCCAGGATATGAA
CCATGACCGTATGTACTGGTATCGACAAGACCCAGGCATGGGACTGAGGCTGATTCATTACTCAGCTGGT
GAAGGTAGCACTGAGAAAGGAGAAGTCCCCAATGGCTACAATGCCTCCAGATTAAACAAAAAGGATTTCC
TGCTCAGGTTGGAGTCAGCTGCTCCCTCCCAGACATCTATGTACTTCTGTGCCAGCAGTGAAGC
>152|TRBV6-2-1*02 IMGT000073|TRBV6-2-1|L-REGION+V-REGION|TR|TRB|None|
02
ATGAGCATCAAGCTCCTGTGCTGTGTGGCCTTTTCTCTCCTGTGGCCAGGTCCAGTGAATGCTGGTGTCA
CTCAGAGCCCAAAATTCCAGATCCTGAAGACAGGACAGAGCATGACACTGCAGTGTGCCCAGGATATGAA
CCATGACTCCATGTACTGGTATTGACAAGACCCAGGCATGGGGCTGAGGCTGATTCATTACTCAGCTACC
GAGGATACCACTGACCCAGGAGAAGTCCCCGATGGCTACAGTGTCTCCAGATTAAACAAAAAGGAGTTCC
TGCTCAGGCTGGAGTCGGCTGCTCCCTCCCAGACATCTGTGTACTTCTGTGCCAGCAGTGAAGC
>153|TRBV6-3*01 NW_001114291|TRBV6-3|L-REGION+V-REGION|TR|TRB|None|01
ATGAGCATCGGCCTCCTGTGCTGTGCGGCCTTTTGTCTCCTGTGGGCAGGTCCAGTGAATGCTGGTGTCA
CTCAGACCCCAAAATTCCAGGTCCTGAAAACAGGACAGAGCATGACACTGCAGTGTGCCCAGGATATGAA
CCATGACCGCATGTACTGGTATCGACAAGACCCAGGCATGGGGCTGAGGCTGATTCATTACTCAGTTGGT
GAAGGTAGCACTGAGAAAGGAGAAGTCCCCGATGGCTACAATGTCACCAGATCAAACACAGAGGATTTCC
CACTCAGACTGGAGTCGGCTGCTCCCTCCCAGACATCTGTGTACTTCTGTGCCAGCAGTTACTC
>154|TRBV6-3*02 IMGT000012|TRBV6-3|L-REGION+V-REGION|TR|TRB|None|02
ATGAGCATCGGCCTCCTGTGCTGTGCGGCCTTTTGTCTCCTGTGGGCAGGTCCAGTGAATGCTGGTGTCA
CTCAGACCCCAAAATTCCAGGTCCTGAAAACAGGACAGAGCATGACACTGCAGTGTGCCCAGGATATGAA
CCATGACTACATGTACTGGTATCGACAAGACCCAGGCATGGGGCTGAGGCTGATTCATTACTCAGTTGGT
GAAGGTAGCACTGAGAAAGGAGAAGTCCCCGATGGCTACAATGTCACCAGATCAAACACAGAGGATTTCC
CACTCAGACTGGAGTCGGCTGCTCCCTCCCAGACATCTGTGTACTTCTGTGCCAGCAGTTACTC
>155|TRBV6-4*01 NW_001114291|TRBV6-4|L-REGION+V-REGION|TR|TRB|None|01
ATGAGCATTGAGCTCCTGTGCTGTGCGGCCTTTTCTCTCCTGTGGGCAGGTCCAGTGAACGCTGGTGTCA
CTCAGACCCCAAAATTCCAGGTCCTGAAGACAGGACAGAGCATGACAGTGCAGTGTGCCCAGGATATGAG
CCATAACTCCAGTTACTGGTATCCACAAGACCCAGGCATGGGTCTGAGGCTGATTCGTTACTCAGCTGCT
GCTGGTGTCACTGACAAAGGAGAAGTCCCCAGTGGCTACAATGTCTCCAGATTAAACACAGAGGATTTCC
CGCTCAGGCTGGAGTCGGCTGCTCCCTCCCAGACATCTGTGTACTTCTGTGCCAGCAGTTACTC
>156|TRBV6-4*03 IMGT000073|TRBV6-4|L-REGION+V-REGION|TR|TRB|None|03
ATGAGCATTGAGCTCCTGTGCTGTGCGGCCTTTTCTCTCCTGTGGGCAGGTCCAGTGAATGCTGGTGTCA
CTCAGACCCCAAAATTCCAGGTCCTGAAGACAGGACAGAGCATGACAGTGCAGTGTGCCCAGGATATGAG

CCATAACTCCAGTTACTGGTATCCACAAGACCCAGGCATGGGTCTGAGGCTGATTCGTTACTCAGCTGCT
GCTGGTGTCACTGACAAAGGAGAAGCCCCCAGTGGCTACAATGTCTCCAGATTAAACACAGAGGATTTCC
CGCTCAGGCTGGAGTCGGCTGCTCCCTCCCAGATATCTGTGTAGTTCTGTGCCAGCAGTTACTC
>157|TRBV6-5*01 IMGT000012|TRBV6-5|L-REGION+V-REGION|TR|TRB|None|01
ATGAGCATTGAGCTCCTGTGCTGTGTGGACTTTTCTCTCCTGTGGGCAGGTCTAGTGAATGTTGGTGTCA
CTCAGACCCCAAAATTCCAAGTCCTGAAGACAGGACAGAGCATGACAGTGCAGTGTGCCCAGGATATGAA
CCATAACTTCATGTACTGGTATCGACAAGACCCAGGCATGGGGCTGAGGTTGATTCATTACTCAGGTGCT
GCTGGTACTACTGATAAAGGAGAAGTCCCCAGTGGCTACAATGTCTCCAGATTAAACACAGAGGATTTCC
CACTCAGACTGGAGTCGGCTGATCCTTCCCAGACATCTGTGTACTTCTGTGCCAGCAGTTACTC
>158|TRBV6-6*01 NW_001114291|TRBV6-6|L-REGION+V-REGION|TR|TRB|None|01
ATGAGCATTGAGCTCCTGTGCTGTGTGGCCTTTTCTCTCCTGTGGGCAGGTCCAGGGAATGCTGCTGTCA
ATCAGACCCCAAAATTCCAGGTCCTGAAGACAGGACAGAGCATGACACTGCAGTGTGCCCAGGATATGAA
CCATGAATGCATGTCCTGGTATCGACAAGACCCAGGCATGGGGCTGAGGCTGATTCATTACTCAGCTGCT
GCTGGTATCACTGACAAAGGAGAAGTCCCCAATGGCTACAATGTCTCTAGATCAAACACAGAGGATTTCC
CGCTCAGGCTGGAGTCGGCTGCTTCCTCCCAGACATCTGTGTACTTCTGTGGCAGCAGTTACTC
>159|TRBV6-6*02 IMGT000073|TRBV6-6|L-REGION+V-REGION|TR|TRB|None|02
ATGAGCATTGAGCTCCTGTGCTGTGCGGCCTTTTCTCTCCTGTGGGCAGGTCCAGGGAATGCTGCTGTCA
ATCAGACCCCAAAATTCCAGGTCCTGAAGACAGGACAGAGCATGACACTGCAGTGTGCCCAGGATATGAA
CCATGAATGCATGTCCTGGTATCGACAAGACCCAGGCATGCGGCTGAGGCTGATTCATTACTCAGCTGCT
GCTGGTATCACTGACAAAGGAGAAGTCCCCAATGGCTACAATGTCTCTAGATCAAACACAGAGGATTTCC
CGCTCAGGCTGGAGTCGGCTGCTTCCTCCCAGACATCTGTGTACTTCTGTGGCAGCAGTTACTC
>160|TRBV6-7*01 NW_001114291|TRBV6-7|L-REGION+V-REGION|TR|TRB|None|01
ATGAGCATCGGCCTCCTGTGCTGTGTGGCCTTTTCTCTCCTGTGGGCAGGTCCAGGGAATGCTGATGTCA
CTCAGACACCAAAATTCCAGGTCCTGAAGACAGGACAGAGCATGACAGTGCAGTGTGCCCAGGATATGAA
CCATGACTGCATGTACTGGTATCGACAAGACCCAGGCATGGGGCTGAAGCTGATTCATTACTCAGTTGGT
GCTGGTATCACTGACAAAGGAGAAGTCCCCAATGGCTACAATGTCTCCAGATTAAACACAGAAGATTTCC
CGCTCAGGCTGGAGTCGGCGGCTCTCTCCCAGACATCTGTGTACTTCTGTGCCAGCAGTGACAC
>161|TRBV6-8*01 IMGT000012|TRBV6-8|L-REGION+V-REGION|TR|TRB|None|01
ATGAGCATTGAGCTCCTGTGTTGTGCGGCCTTTTCTCTCCTGTGGACAGGTCCAGTGAATGCTGGTGTCA
CTCAGACACCAAAATTCCAGGTCCTGAAGACAGGACAGAGCCTGACAGTGCAGTGTGCCCAGGATATGAA
ACATGACTACATGTTCTGGTATCGACAAGACCCAGGCATGGGGCTGAGACTGATTTATTACTCAGTTACT
GCTGGTATCACTACCAAAGGAGAAGTCCCCAATGGCTACAATGTCTCCAGAATAAACACAGAGGATTTCC
TGCTCAGGCTGGAGTCGGCTGATCCCTCCCAGACATCTGTGTACTTCTGTGCCAGCAGTGAATC
>162|TRBV6-8*02 IMGT000073|TRBV6-8|L-REGION+V-REGION|TR|TRB|None|02
ATGAGCATTGAGCTCCTGTGTTGTGCGGCCTTTTCTCTCCTGTGGACAGGTCCAGTGAATGCTGGTGTCA
CTCAGACACCAAAATTCCAGGTCCTGAAGACAGGACAGAGCCTGACAGTGCAGTGTGCCCAGGATATGAA
ACATGACTACATGTTCTGGTATCGACAAGACCCAGGCATGGGGCTGAGACTGATTTATTACTCAGTTACT
GCTGGTATCACTACCAAAGGAGAAGTCCCCAATGGCTACAATGTCTCCAGAATAAACACAGAGGATTTCC
TGCTCAGGCTGGAGTCGGCTGATCCCTCCCAGACATCTGTGTACTTCTGTGGCAGCAGTTACTC
>163|TRBV7-10*01 NW_001114291|TRBV7-10|L-REGION+V-REGION|TR|TRB|None|
01
ATGGGCACCAGGCTCCTCTGCTGGGTGGCCCTGTGTGTCCTGTGGGCAGATCACGCAGATGCTGGAGTCT
CCCAAGACCCCAGACACAAGATCACAAAGACGGGACAGAATGTATCTTTCAAGTGTGTTCCAATTTCCGA
ACACAACCGCCTTTACTGGTACCGACAGACCTTGGGGCAGGGCCCAGAGTTTCTGACTTACTTCCAGAAT
GAAGCTCAACAAGACAGATCAGGGCTGTTCAGTGATCGGTTCTCTGCAGAGAGGCCTGAGGGATCTATCT
CCACCCTGATGATCCAGCACACAGAGCAAGGGGACTTGGCCATGTATCTCTGTGCCAGCAGCTTAGC
>164|TRBV7-10*02 IMGT000073|TRBV7-10|L-REGION+V-REGION|TR|TRB|None|02
ATGGGCACCAGGCTCCTCTGCTGGGTGGCCCTGTGTGTCCTGTGGGCAGATCACGCAGATGCTGGAGTCT
CCCAAGACCCCAGACACAAGATCACAAAGACGGGACAGAATGTATCTTTCAAGTGTGTTCCAATTTCCGA
ACACAACCGCCTTTACTGGTACCGACAGACCTTGGGGCAGGGCCCAGAGTTTCTGACTTACTTCCAGAAT

```
GAAGCTCAACAAGACAGATCAGGGCTGTTCAGTGATCGGTTCTCTGCAGAGAGGCCTGAGGGATCTATCT
CCACCCTGATGATCCAGCACACAGAGCAAGGGGACTCGGCTGTGTATCTCTGTGCCAGCAGCTTAGC
>165|TRBV7-2*03 IMGT000073|TRBV7-2|L-REGION+V-REGION|TR|TRB|None|03
ATGGGCACCAGGCTCCTCTGCTGGGCAGCCCTGTGCCTCCTGGGGGCAGATCACACAGGTGCTGGAGTTT
CCCAGTCCCCCAGGAACAAGGTCACAGAGGAGGGAAAGGATGTAGTGCTCAGGTGTGATCCAATTTCAGG
TCATACCGCCCTTTATTGGTACCGACAGAGCCTGGGGAAGGGCCTGGAGTTTTTAATTTACTTCCAAGGC
AACGATGCACCAGACAAATCAGGGCTGCCCAGTGGTCGGTTCTCTGCAGAGAGGACTGAGGGATCCGTCT
CCACTCTGAAGATCCAGCGCACAGAGCAGGGGGACTCGGCCGTGTATCTCTGTGCCAGCAGCTTAGC
>166|TRBV7-3*01 NW_001114291|TRBV7-3|L-REGION+V-REGION|TR|TRB|None|01
ATGGGCACCAGGCTCCTCTGCTGGGCTGCCCTGTGCCTCCTGGGGGCAGATCACACAGGTGCTGGAGTTT
CCCAGTCCCCCAGTAACAAGGTCACAGAGAAGGGAAAGGATGTAGCGCTCAGGTGTGATCCAATTTCAGG
TCATGCTGTCCTTTACTGGTACCGACAGAACCTGGGGCAGGGCCTAGAGTTTCTAATTTACTTCCAAGGC
ACGGGTGCAGCAGATGACTCAGGGCTGCGCAGTGATCGGGTCTCTGCAACACGGCCTGAAGGATCCGTCT
CTACTCTGAAGATCCAACACACAGAGCAGGGGGACTCAGCCATGTACTTCTGTGCCAGCAGCTTAAC
>167|TRBV7-4*01 NW_001114291|TRBV7-4|L-REGION+V-REGION|TR|TRB|None|01
ATGGGCACCAGGCTCCTCTGCTGGGTGGTCCTGGGTTTCCTAGGGACAGATCACACAGGTGCTGGAGTCT
CGCAGTCCCCAAGATACAAAGTCACGAAGAGGGGACAGGATGTAGCTCTCAGGTGTGATCCAATTTCGGG
TCATGAGTACCTTTACTGGTACCAACAGGCCCTGGGGCAGGGCCCAGCGTTTCTGACTTACTTCCAGAAT
GATGCTCAACGAGACAAATCAGGGCTGCCCAATGATCGGTTCTCTGCAGAGAGGACTGAGGGATCCGTCT
CCACTCTGAAGATCCAGCGCACACAGCAGGGGGACTCAGCCGTGTATCTCTGTGCCAGTAGCTTAGC
>168|TRBV7-4*02 IMGT000012|TRBV7-4|L-REGION+V-REGION|TR|TRB|None|02
ATGGGCACCAGGCTCCTCTGCTGGGTGGTCCTGGGTTTCCTAGGGACAGATCACACAGGTGCTGGAGTCT
CGCAGTCCCCAAGATACAAAGTCACGAAGAGGGGACAGGATGTAGCTCTCAGGTGTGATCCAATTTCGGG
TCATGAGTACCTTTACTGGTACCAACAGGCCCTGGGGCAGGGCCCAGCGTTTCTGACTTACTTCCAGAAT
GATGCTCAACGAGACAAATCAGGGCTGCCCAATGATCGGTTCTCTGCAGAGAGGACTGAGGGATCCGTCT
CCACTCTGAAGATCCAGCGCACACAGCAGGGGGACTCAGCCGTGTATCTCTGTGCCAGCAGCTTAGC
>169|TRBV7-4*03 IMGT000073|TRBV7-4|L-REGION+V-REGION|TR|TRB|None|03
ATGGGCACCAGGCTCCTCTGCTGGGTGGTCCTGGGTTTCCTAGGGACAGATCACACAGGTGCTGGAGTCT
CGCAGTCCCCAAGATACAAAGTCACGAAGAGGGGCCAGGATGTAGCTCTCAGGTGTGATCCAATTTCCGG
TCATGATTACCTTTACTGGTACCAACAGGCCCTGGGGCAGGGCCCAGCGTTTCTGACTTACTTCCAGGAT
GATGCTCAACGAGACAAATCAGGGCTGCCCAATGATCGGTTCTCTGCAGAGAGGACTGAGGGATCCGTCT
CCACTCTGAAGATCCAGCGCACAGAGCAGGGGGACTCGGCCGTGTATCTCTGTGCCAGCAGCTTAGC
>170|TRBV7-5*01 NW_001114291|TRBV7-5|L-REGION+V-REGION|TR|TRB|None|01
ATGGGCACCAGGCTCCTCTGCTGGGTGGTCCTGGGTTTCCTAGGGACAGATCACACAGGTGCTGGAGTCT
CCCAGTCCCCGAGGTACAAAGTCACGAAGAGGGGCCAGGATGTAGCTCTCAGGTGTGATCCAATTTCGGG
TCATGTAGCCCTTTATTGGTACCGACGGACTCTGGGACAGGGGCCAGAGCTTCTGACTTACTTCCAGAAT
GATGCTCAACCAGACAAATCAGGGCTGCCCAGTGATCGCTTCTCTGCAGAGAGGACTGAGGGATCCGTCT
CCACTCTGAAGATCCAGTGCACAGAGCAGGGGGACTCAGCCGTGTTTCTCTGTGCCAGCAGCTTAGC
>171|TRBV7-5*02 IMGT000073|TRBV7-5|L-REGION+V-REGION|TR|TRB|None|02
ATGGGCACCAGGCTCCTCTGCTGGGTGGTCCTGGGTTTCCTAGGGACAGATCACACAGGTGCTGGAGTCT
CCCAGTCCCCGAGGTACAAAGTCACGAAGAGGGGACAGGATGTAGCTCTCAGGTGTGATCCAATTTCGGG
TCATGTAGCCCTTTATTGGTACCGACGGACTCTGGGACAGGGGCCAGAGCTTCTGACTTACTTCCAGAAT
GATGCTCAACCAGACAAATCAGGGCTGCCCAGTGATCGCTTCTCTGCAGAGAGGACTGAGGGATCCGTCT
CCACTCTGAAGATCCAGCGCACAGAGCAGGGGGACTCGGCCGTGTTTCTCTGTGCCAGCAGCTTAGC
>172|TRBV7-6*01 NW_001114291|TRBV7-6|L-REGION+V-REGION|TR|TRB|None|01
ATGGGCACCAGGCTCCTCTGCTGGGTGGTCCTGGGTTTCCTAGGGACAGGTCACACAGGTGCTAGAGTCT
CCCAGTCTCCAAGGTACAAAGTCACAAAGAGGGGCCAGGATGTAGCTCTCAGATGTGATCCAATTTCGGG
TCATGCAACTCTTTATTGGTACCAACAGGCCCTAGGACAGGGCCCAGAGCTTCTGACTTACTTCAGTTAT
GAAGCTCAACAAGATAAATCAGGGCTGCCCAGTGATCGCTTCTCTGCAGAGAGGCCCGAGGGATCCGTCT
CCACTCTGAAGATCCAGCGCACAGAGCAGGGGGACTCGGCCGTGTATCTCTGTGCCAGCAGCTTAGC
```

>173|TRBV7-6*02 IMGT000073|TRBV7-6|L-REGION+V-REGION|TR|TRB|None|02
ATGGGCACCAGGCTCCTCTGCTGGGTGGTCCTGGGTTTCCTAGGGACAGGTCACACAGGTGCTAGAGTCT
CCCAGTCTCCAAGGTACAAAGTCACAAAGAGGGGACAGGATGTAGCTCTCAGGTGTGATCCAATTTCGGG
TCATGCAACTCTTTATTGGTACCAACGGGCCCTAGGACAGGGCCCAGAGCTTCTGACTTACTTCAGTTAT
GAAGCTCAACAAGATAAATCAGGGCTGCCCAGTGATCGCTTCTCGGCAGAGAGGCCCGAGGGATCCGTCT
CCACTCTGAAGATCCAGCGCACACAGCAGGGGGACTCGGCCGTGTATCTCTGTGCCAGCAGCTTAGC
>174|TRBV7-7*01 NW_001114291|TRBV7-7|L-REGION+V-REGION|TR|TRB|None|01
ATGGGCACCAGGCTCCTCTGCTGGGTGGTCCTGGGTTTCCTAGGGACAGATCACACAGGTGCTGGAGTCT
CCCAGTCCCCAAGGTACAAAGTCACAGAGAGGGGACAGGATGTAGCTCTCAGGTGTGATCCAATTTCGGG
TCATGATTACCTTTACTGGTACCAACAGGCCCTGGGGCAGGGCCCAGAGCTTCTGACTTACTTCAATGAC
AAAGCTCAACCAGACAAATCAGGGCTGCCCAATGATCGGTTCTCTGCAGAGAGGCCTGAGGGATCCGTCT
CCACTCTGAAGATCCAGCGAACAGAGCAACGAGACTCGGCCGTGTATCTCTGTGCCAGCAGCTTAGC
>175|TRBV7-7*02 IMGT000073|TRBV7-7|L-REGION+V-REGION|TR|TRB|None|02
ATGGGCACCAGGCTCCTCTGCTGGGTGGTCCTGGGTTTCCTAGGGACAGATCACACAGGTGCTGGAGTCT
CCCAGTCCCCAAGGTACAAAGTCACAAAGAGGGGACAGGATGTAGCTCTCAGGTGTGATCCAATTTCGGG
TCATGATTACCTTTACTGGTACCAACAGGCCCTGGGGCAGGGCCCAGAGCTTCTGACTTACTTCAATTAC
AAAGCTCAACCAGACAAATCAGGGCTGCCCAATGATCGCTTCTCTGCAGAGAGGCCTGAGGGATCCATCT
CCACTCTGAAGATCCAGGGAACAGAGCAACGAGACTCGGCCGTGTATCTCTGGGCCAGCAGCTTAGC
>176|TRBV7-7-1*02 IMGT000073|TRBV7-7-1|L-REGION+V-REGION|TR|TRB|None|
02
ATGGGCACCAGGCTCCTCTGCTGGGTGGTCCTGGGTTTCCTAGGGACAGATCACACAGGTGCTGGAGTCT
CACAGTTCCCAAGATACAGAGTCACAAAGAGGGGACAGGATGTAACTCTCAGGTGTGATCCAATTTCGGG
TCATGAGTACCTTTACTGGTACCAACAGGCCCCGGGGCAGGGCCCAGAGTTTCTGACTTACTTCCAGAAT
GATGCTCAACGAGACAAATCAGGGCTGCCCAATGATCGCTTCTCTGCAGAGAGGACTGAGGGATCCGTCT
CTACTCTGAAGATCCAGCGCACAGAGCAGGGGGACTCGGCCGTGTATCTCTGTGCCAGTAGCTTAGC
>177|TRBV7-9*01 NW_001114291|TRBV7-9|L-REGION+V-REGION|TR|TRB|None|01
ATGGGCACCAGGCTCCTCTGCTGGGTGGTCCTGGGTTTCCTAGGGACAGATCACACAGGTGCTGGAGTCT
CCCAGTCTCCCAGGTACAAAGTAATAAAGAAGGGACAGGATGTAGCTCTGAGGTGTGATCCAATTTCGGG
TCATGCAGCCCTCTATTGGTACCAACAGGCCCTGGGGCAGGGCCCAGAGTTTCTGACTTACTTCAATTAT
AAAGCTCAACCAGACAAATCAGGGCTGCCCAATGATCGGTTCTCTGCAGAGAGGCCTGAGGGATCTGTCT
CCACTCTGACGATCCAGCGAACAGAGCAGCAGGACTCGGCCGTGTATCTCTGTGCCAGCAGCTTAGC
>178|TRBV7-9*02 IMGT000073|TRBV7-9|L-REGION+V-REGION|TR|TRB|None|02
ATGGGCACCAGGCTCCTCTGCTGGGTGGTCCTGGGTTTCCTAGGGACAGATCACACAGGTGCTGGAGTCT
CCCAGTCTCCTAGGTACAAAGTAATAAAGAAGGGACAGGATGTAGCTCTGAGGTGTGATCCAATTTCGGG
TCATGCAGCCCTCTATTGGTACCAACAGGCTCTGGGGCAGGGCCCAGAGTTTCTGACTTACTTCAATTAT
CAAGCTCAACCAGACAAATCAGGGCTGCCCAATGATCGGTTCTCTGCAGAGAGGCCTGAGGGATCTGTCT
CCACTCTGACGATCCAGCGAACAGAGCAGGGGGACTCGGCCGTGTATCTCTGTGCCAGCAGCTTAGC
>179|TRBV9*01 NW_001114291|TRBV9|L-REGION+V-REGION|TR|TRB|None|01
ATGGGCTTCAGGCTCCTCTGCTGTGTGGCCTTTTGTCTCCTGGGAGCAGGCTCAGTGGATTGTGGAGTCA
CACAAACCCCAAAGCACCTGATCACAGCAATTGGACAGCAAGTGACGCTGAGATGCTCCCCTAGGTCTGG
AGACCTCTCCGTGTACTGGTACCAACAGAGCCTGGGCCAGGGCCTCCAGTTCCTCATTCAGTATTATAAT
GGGGAAGAGAGAGCAAAAGGAAACATTCTTGAAAGATTCTCAGCACAACAGTTCGCTGACTTGCACTCTG
AACTAAACCTGAGCTCTCTGGAGCTGGGGGACTCGGCGTTGTATTTCTGTGCCAGCAGCTTAG
>180|TRBV9*02 IMGT000012|TRBV9|L-REGION+V-REGION|TR|TRB|None|02
ATGGGCTTCAGGCTCCTCTGCTGTGTGGCCTTTTGTATCCTGGGAGCAGGCTCAGTGGATTGTGGAGTCA
CACAAACCCCAAAGCACCTGATCACAGCAATTGGACAGCAAGTGACGCTGAGATGCTCCCCTAGGTCTGG
AGACCGCTCCGTGTACTGGTACCAACAGAGCCTGGGCCAGGGCCTCCAGTTCCTCATTCAGTATTATAAT
GGGGAAGAGAGAGCAAAAGGAAACATTCTTGAAAGATTCTCAGCACAACAGTTCGCTGACTTGCACTCTG
AACTAAACCTGAGCTCTCTGGAGCTGGGGGACTCGGGGTTGTATTTCTGTGCCAGCAGCGAAG
>181|TRBV9*03   IMGT000073|TRBV9|L-REGION+V-REGION|TR|TRB|None|03

```
ATGGGCTTCAGGCTCCTTTGTTGTGTGGCCTTTTGTCTCCTGGGAGCAGGCTCAGTGGATTGTGGAGTCA
CACAAACCCCAAAGCACCTGATCACAGCAATTGGACAGCAAGTGACGCTGAGATGCTCCCCTAGGTCTGG
AGACCGCTCCGTGTACTGGTACCAACAGAGCCTGGGCCAGGGCCTCCAGTTCCTCATTCAGTATTATAAT
GGGGAAGAGAGAGCAAAAGGAAACATTCTTGAAAGATTCTCAGCACAACAGTTCGCTGACTTGCACTCTG
AACTAAACCTGAGCTCTCTGGAGCTGGGGGACTCGGGGTTGTATTTCTGTGCCAGCAGCTTAG
>182|TRDV1*01 IMGT000013|TRDV1|L-REGION+V-REGION|TR|TRD|None|01
ATGCTGTTCTCCAGCTTGCTGTGCATATTTGTGGCCTTCAGCTACTCTGGATTCAGTGTGGCCCAGAAGG
TTACTCAAGCCCAGTCATCAGTATCCATGCCAGTGGAGAAAGCAGTCACCCTGAACTGCCAGTATGAAAC
AAGTTCGTGGTCATATGACCTTTTTTGGTACAAGCAACTTCCCGGCAAAGAGATGATTTTCCTTATTCGC
CAGGGTTCTTCTGAACAGAATGCAAGAGATGGTCGCTATTCTGTCAACTTCAAGAAAGAAGCTAGCTTCA
TCGCCTTAACCATTTCAGCCTTACAGCTAGAAGACTCAGCAACATACTTCTGTGCTCTCCGGGAACT
>183|TRDV1*02 IMGT000076|TRDV1|L-REGION+V-REGION|TR|TRD|None|02
ATGCTGTTCTCCAGCTTGCTGTGCATATTTGTGGCCTTCAGCTACTCTGGATCCAGCGTGGCCCAGAAGG
TTACTCAAGCCCAGTCATCAGTATCCATGCCAGTAGGGAAAGCAGTCACCCTGAACTGCCAGTATGAAAC
AAGTTCGTGGTCATATTACCTTTTTTGGTACGAGCAACTTCCCGGCAAAGAGATGATTTTCCTTATTCGC
CAGGGTTCTTCTGAACAGAATGCAAGAAATGGTCGCTATTCTGTCAACTTCAAGAAAGAAGCTAGCTCCA
TCGCCTTAACCATTTCAGCCTTACAGCTAGAAGACTCAGCAATGTACTTCTGTGCTCTCTGGGAACT
>184|TRDV1-1*01 IMGT000076|TRDV1-1|L-REGION+V-REGION|TR|TRD|None|01
ATGCTGTTCTCTAGCTTGCTGTGCATATTTGTGGCCTTCAGCTACTCTGGATCCAGCGTGGCCCAGAAGG
TTACTCAAGCCCAGTCATCAGTATCCATGCCAGTAGGGAAAGCAGTCACCCTGAACTGCCAGTATGAAAC
AAGTTCGTGGTCATATTACCTTTTTTGGTACAAGCAACTTCGCGGCAAAGAGATGATTTTCCTTATTCGC
CAGGGTTCTTCTGAACAGAATGCAAGAAATGGTCGCTATTCTGTCAACTTCCAGAAAGCAGCTAGCTCCA
TCACCGTAACCATTTCAGCCTTACAGCTAGAAGACTCAGCAACATACTTCTGTGCTCTCCGGGAACT
>185|TRDV2*01 IMGT000013|TRDV2|L-REGION+V-REGION|TR|TRD|None|01
ATGCAGAGGATCTCCTCCCTCATCCATCTCTCCCTCTTCTGGGCAGGAGTCATGTCAGCTGTTGAGTTGG
TGCCTGAACACCAAACAGTGATTGTGTCAGTGGGGGACCCTGCCACCCTCAAGTGCTCCATGAAAGGAGA
AGCAATCAGTAACTACTATATCAACTGGTACAGGAAGACCCTAGGTAACACAATGACTTTCATATACCGA
GAAAAGGGCATCTATGGCCCTGGTTTCAAGGACAATTTCCAAGGTGACATTGATACTGAAGAGAACCAGG
CTGTACTTAAGATCCTTGCACCATCAGAGAGAGATGAAGGGTCTTACTACTGTGCCAGTGACATC
>186|TRDV2*02 IMGT000076|TRDV2|L-REGION+V-REGION|TR|TRD|None|02
ATGCAGAGGATCTCCTCCCTCATCCATCTCTCCCTCTTCTGGGCAGGAGTCATGTCAGCTGTTGAGTTGG
TGCCTGAACACCAAACAGTGATTGTGTCAGTGGGGGACCCTGCCACCCTCAAGTGCTCCGTGAAAGGAGA
AGCAATCAGTAACTACTATATCAACTGGTACAGGAAGACCCAAGGTAACACAATGACTTTCATATACCGA
GAAAAGGGCATCTATGGCCCTGGTTTCAAGGACAATTTCCAAGGTGACATTGATACTGAAGAGAACCAGG
CTGTACTTAAGATCCTTGCACCATCAGAGAGAGATGAAGGGTCTTACTACTGTGCCAGTGACATC
>187|TRDV3*01 IMGT000013|TRDV3|L-REGION+V-REGION|TR|TRD|None|01
ATGATTCTTACTGTGGGCTTTAGCTTTTTGTTTTTCTACAGGGGCATGCTGTGTGACAAAGTAACTCAGA
GTTCCCCGGACCAGATGGTGGCGAGTGGCAGTGAGGTGGTACTGCTCTGCACTTACGACAGTACATATTC
AGATCCAGGTTTATTCTGGTACCGCATAAGGCCAGATTATAATTTTCAGTTTGTCTTCTATGGGGATAAA
AGCAGATCTCACGGTTCATATTTTGCTAAAGGACGGTTTTCTGTGAAGCACCTTCTAACCCAGAAAGCCT
TTCACTTGGTAATCTCTCCAGTGAGTACTGAAGACAGTGCCACTTACTACTGTGCCATGAG
>188|TRDV3*02 IMGT000026|TRDV3|L-REGION+V-REGION|TR|TRD|None|02
ATGATTCTTACTGTGGGCTTTAGCTTTTTGTTTTTCTACAGGGGCATGCTGTGTGACAAAGTAACTCAGA
GTTCCCCGGACCAGATGGTGGCGAGTGGCAGTGAGGTGGTACTGCTCTGCACTTACGACAGTACATATTC
AGATCCAGGTTTATTCTGGTACCGCATAAGGCCAGATTATAATTTTCAGTTTGTCTTCTATGGGGATAAA
AGCAGATCTCACGGTTCATATTTTGCTAAAGGACGGTTTTCTGTGAAGCACCTTCTGACCCAGAAAGCCT
TTCACTTGGTAATCTCTCCAGTGAGTACTGAAGACAGTGCCACTTACTACTGTGCCATGAG
>189|TRDV4*01 IMGT000013|TRDV4|L-REGION+V-REGION|TR|TRD|None|01
ATGCCAAAGACCTGTCTCCTCTTCTTCCTCACCATCTTCTGCGCAGGGGTCTCATTGGATATAATTTTGA
AACCGGATACCAAAGCACTGACTGTCCTCATTGGGGAGGCTGCTACCTTCCGTTGCAGTGTAACAGGAGG
```

CGACCTGAAGAACTATCAAATAAGCTGGTATAAGAAGAGTGAAGATAACTCTCTGATTTTGATCTCCAAA
CTAAGCAACAATTCTAATGACAATTTAGGGAATAATTTCAAGGTGAAAATCGATACTTTAAAAAGTCAAT
TTATGCTTGACATTCAAAAAGCAACAACACAAGATGTTGGGACTTACTACTGTGCGTCTGATATC
>190|TRGV1*01 IMGT000059|TRGV1|L-REGION+V-REGION|TR|TRG|None|01
ATGCGGTGGGCCCTAGCCCTGGTTCTAGCTCTCCTGTCTCCTGCCAGTCAGAAATCTTCCAACTTGGAAG
GGAAAACGAAGTCAATCACCGGGCCTACTGGGTCACCTGCTGAAATCACCTGTGATCTTCCTGGAGTCAG
TACCTTATACATCCACTGGTACCTACACCAGGAGGGGAAGGCCCCACAGCGTCTTCTGTACTATGAACCC
TACTACTTCACGGTTGTGTTGGAATCAGGAATCAGTCCAGGAAAGTATGACACTGCAAGCACAAGGAAGA
GCTGGAATTTGAGACTGCAAAATCTAATTGAAAATGATTCTGGGGTCTATTACTGTGCCACCTGGGACAG
G
>191|TRGV10*01 IMGT000059|TRGV10|L-REGION+V-REGION|TR|TRG|None|01
ATGTCACTGCTGGAAGCATTCGCCTTCTCGTCCTTCTGGGCACTTGGACTTGGATTATCGAAAGTGGAGC
AGTTCCAGCTATCCATTTCCACGGAAGTCAAGAAAAGTATTGACATATCTTGCAAGATAAGGAGCACAAA
CTTTGAAAACGATGTCATTCACTGGTACCGGCAGAAACCGAATCAGGCTTTGGAGCATCTGATCTATATT
ATCTCAACAAAATCCGCAGCTCATGGCAGCATGGGTAAGAGGAGTAACAAAGTGGAAGCGAGAAAGAATT
CTCAAACCCTCACTTCAATCCTTACCATCAAGTCCATAGAGAAAGAAGACATGGCCATTTACTACTGTGC
AGGATGGGATTA
>192|TRGV11*01 IMGT000059|TRGV11|L-REGION+V-REGION|TR|TRG|None|01
ATGCCACTGGTAGTAGCCATTATCTTCTTCTCCCTCTGGGCTTTTGGACTTGGGCATTTGAAGCAACCTG
AAACATATATTTCCAGACCAACAAATAAGAGTGCCCACATATCTTGAAAGGCATCCATCCAAGGCTTTAG
CAGTAAAATCATACACTCGTACTGGCAGAAACCAAACCAAAGCTTAGAATATTTATTACATGTCTTCTTG
GCAATCTCTGCTCAAGATGGCTCAGGTGGGAAGACTAAGACTAAGTTTCTTAAGACTAAGAAACTTGAGG
TAAGTAAAAATGTTCACACTTCCGCTTCCACTTTGAAAATAAATTTCTTAGAGAAAGATGAGGTGGTATA
CCACTGTGCCTGCTGGATTAGGCGC
>193|TRGV2*01 IMGT000059|TRGV2|L-REGION+V-REGION|TR|TRG|None|01
ATGTGGTGGGCCCCAGCCCTGCTTCTAGCTCTCCTGTCTCCTGCCAGTCAGAAATCTTCCAATTTGGAAG
GGAGAACAAAGTCAGTCACCAGGCCGACTGGGTCATCCGCTGAAATCACCTGTGATCTTCCTGAAGTCAG
TAGCTTCTACATCCACTGGTACCTACACCAGGAGGGGAAGGCCCCACAGCGTCTTCTGTACTATGACTCC
TCCAACTCCAGGGTTGTGTTGGAATCAGGAATCAGTCCAGGAAAGTATGATACTTATGGAAGCACAAGGA
GCAACTTGAGATTAATACTGCGAAATGTAATTGAAAATGATTCTGGGGTCTATTACTGTGCCAACTGGGA
CAGG
>194|TRGV3*01 IMGT000059|TRGV3|L-REGION+V-REGION|TR|TRG|None|01
ATGCGGTGGGCCCTAGCCCTGCTTCTAGCTTTCTTGTTTCCTGCCGGTCAGAAATCTTCCAACTTGGAAG
GGATAACAAAGTCAGTCACCAGGCCAACTGGGTCATCTGCTCAAATAACTTGTGATTTTTCTGGAGACAG
TAACTTTTATATCCACTGGTACCTACACCAGGAGGGGAAGGTCCCACAGCGTCTTCTGTACTATGACGTC
TCCAACTCAAGGGATGTGTTGGAATCAGGACTCAGTCCAGGAAAGTATTATACTCATACACGCACAAGGT
GGAGTTGGATATTGATACTGCGAAATCTAATTGAAAATGATTCTGGGGTCTATTACTGTGCCAACTGGGA
CAGG
>195|TRGV6*01 IMGT000059|TRGV6|L-REGION+V-REGION|TR|TRG|None|01
ATGCGGTGGACCCTAGCCGTGCTTCTAGCTTTCCTGTCTCCTGCCAGTCACATATCTACTAACTTGGAAG
CAAGAAGAAAGTCAGGCACCAGGGGGTCAGGGTCATCTGTTGTTATCACCTGTGATCTTCCTGTAGAAAA
TACCTTCTGCATCCACTGGTACCAACACCAGGAGGAGAAGGCCCCACAGCATCTTCTGTGCTATGACCCC
TTCACTCCAGGGATGTGTTGGAATCAGGAGTCAGCCTAGGAAAGCATGATAATTATGGAAGTACAAGGAT
AAGTCAGAAATTTATACCTCTAAAACTAAATGAAAGTGACTCTGGGGTCTATTACTGTGCCAACTGGGAC
AGG
>196|TRGV8*01 IMGT000059|TRGV8|L-REGION+V-REGION|TR|TRG|None|01
ATGTGGTGGGCCCCAGTCCTGCTTCTAGCTCTCCTGTCTCCTGCCAGTCAGAAATCTTCCAATTTGGAAG
GGAGAACAAAGTCAGTCACCAGGCCAACTGGGTCATCTGCTGAAATCACCTGTGATCTTCCTGAAGTCAG
TAGCTTCTACATCCACTGGTACCTACACCAGGTGGGGAAGGCCCCACAGCGTCTTCTGTACTATGACACC
TCCAACTCCAGGGTTGTGTTGGAATCAGGAATCAGTCCAGGAAAGTATGATAGTTATGGAAGCACAAGGA

ACAACTTGAGATTGAGACTGCGAAATCTAATTGAAAATGATTCTGGGGTCTATTACTGTGCCAACTGGGA
CAGG
>197|TRGV9*01 IMGT000059|TRGV9|L-REGION+V-REGION|TR|TRG|None|01
ATGCTGTCACTGCTCCACGCGTCAACACTGGCAGTCCTTGGGGCTCTGTGTGTATATGGTGCAGGTCACC
TAGAGCAACCTCAAATTTCCAGTACTAAAATGCTGTCAAAAACAGCCCGCCTGGAATGTGTGGTGTCTGG
AGTAACAATTTCTGAAACATCTATATATTGGTATCGAGAAAGACCTGGTGAAGTCATACAGTTCCTGGTG
TGCATTTTTTATGACGGCACTGTCAAAAAGGAATCCAGCATTCCATCGGGCAAATTTGAGGTGGATAGGA
TACCCAAAACGTCTACATCCACCCTCACCATTCACAATGTAGAGAAACAGGACATAGCTACCTACTATTG
TGCCTTGTGGGAGGTG
>198|TRGVA*01 IMGT000059|TRGVA|L-REGION+V-REGION|TR|TRG|None|01
GTGCTGGGCCGCCTTGCCTTCCTTTGGTCCATCCTGTTTCCAGCTGGCTGGTGGCTCATCAGGCCGAAGC
AGCTGGCCCATGTCCTGGGGCGCTAGGGAAACTCGGTCATCCTGCAGTGCTTGGTCTGCACCAGGATCAG
CTACATCCACTGGTACCGGCAGCAGAAGGGCCAGGTCCCTGAGGGGCTCCACCAGCTGGCCATGTCCACG
TTGGATGTGCAGTGGGATTCCATCTTGAAAGTAGATAAAATCACAGCCAAGGATGGCAGCAGCTGTACCC
TGGCAGCGCTGAAGCTGGAGACAGGCATCGAGGGCATGAACTACTGCACTGCCTGGGACCTG
>199|TRGVB*01 IMGT000059|TRGVB|L-REGION+V-REGION|TR|TRG|None|01
AAGTGATCTGCCCTCCTTGGTCTCCCAAAGTACTGTGATGACATTAAATTTAAAGCAATAAAAAATGTCA
ACTGCATTTTTGTCAACAGAGCAACAGATAAAAGTGTCTAGGTATCTTGTGTGGTGTCCACTGAAGACTA
TGTAAATATAGTTATACACTGGTACCAGCAGAAACTGATTCTAAATTATGAACCATCTGACATATATCAC
TTCAATAGAAAGTCCCAGTTCAGGTTTGGTTAGGTGAGAAAAACAACAAACTTGAGGTAAGAACAAATTT
TCAAATGCCTACTTAAGTCTTTACTATAAACTTCATAGAAAAGGAAGCGGAGGCCATATATACTGCACTG
CTTAGGACC
>200|TRBD1*01 L43137|TRBD1|D-REGION|TR|TRB|None|01
GGGACAGGGGGC
>201|TRBD2*01 L43138|TRBD2|D-REGION|TR|TRB|None|01
GGGACTGGGGGGAG
>202|TRDD1*01 IMGT000013|TRDD1|D-REGION|TR|TRD|None|01
GAAATAGTTGTT
>203|TRDD2*01 IMGT000013|TRDD2|D-REGION|TR|TRD|None|01
CCTTCGTAC
>204|TRDD3*01 IMGT000013|TRDD3|D-REGION|TR|TRD|None|01
ACTGGGTGGATACG
>205|TRDD4*01 IMGT000013|TRDD4|D-REGION|TR|TRD|None|01
ACTGGTGGGATACG
>206|TRAJ1*01 IMGT000013|TRAJ1|J-REGION|TR|TRA|None|01
ATATGAAGGTGTTACCTCCCAGTAGCAATTTGGCAAAGGAACCAGAGTTTCCATTTCTCACC
>207|TRAJ10*01 IMGT000013|TRAJ10|J-REGION|TR|TRA|None|01
ATACTCATGGGAGGAGGAAACAAACTCACCTTTGGGACAGGCACTCAGCTAAAAGTGGAACTGA
>208|TRAJ11*01 IMGT000013|TRAJ11|J-REGION|TR|TRA|None|01
TGAATTCAGGATACAGCACACTCACCTTTGGGAAGGGGACAATGCTTCTAGTCTTTCCAG
>209|TRAJ12*01 IMGT000013|TRAJ12|J-REGION|TR|TRA|None|01
GGATGGATGGCGGCTATAAATTGATCTTTGGGAGTGGGACCAGACTCCTGGTCAGGCCTG
>210|TRAJ13*01 IMGT000013|TRAJ13|J-REGION|TR|TRA|None|01
TGAATTCTGGAAGCTACCAGAAAGTTACCTTTGGAAATGGAACAAAGCTCCAAGTCATCCCAA
>211|TRAJ14*01 IMGT000013|TRAJ14|J-REGION|TR|TRA|None|01
CTGTGATTTATAACACATTCATCTCTGGGAGTGGGACAAGATTATCAGTAAAACCTG
>212|TRAJ15*01 IMGT000013|TRAJ15|J-REGION|TR|TRA|None|01
CCAACCAGGCTGGAACTGCACTGATCTTTGTGAAGGGAACCACCTTATCAGTGAGTTCCA
>213|TRAJ16*01 IMGT000013|TRAJ16|J-REGION|TR|TRA|None|01
GGTTTTCAGATGGCCAGAAGCTGCTCTTTGGAAGGGGGACCATGTTAAAGGTGGATCTTA

>214|TRAJ17*01 IMGT000013|TRAJ17|J-REGION|TR|TRA|None|01
TGATCAATGTTGCAAGCAACAAGCTAACTTTTGGAGGAGGAACCAGGGTGCTAGTTAAACCAA
>215|TRAJ18*01 IMGT000013|TRAJ18|J-REGION|TR|TRA|None|01
CCGACAGAGGCTCAACCCTGGGGAAGCTATACTTTGGAAGAGGAACTCAGTTGACTGTCTGGCCTG
>216|TRAJ19*01 IMGT000013|TRAJ19|J-REGION|TR|TRA|None|01
GCTATCAAAGATTTTACAAGTTCAGCTTTGGAAAGTCATCCAAATATAATGTCACTCCAA
>217|TRAJ2*01 IMGT000013|TRAJ2|J-REGION|TR|TRA|None|01
TGAATACTGGAGGAACAATTGATAAACTCACATTTGGGAAAGGAACCCATGTGTTCATTATATCTG
>218|TRAJ20*01 IMGT000013|TRAJ20|J-REGION|TR|TRA|None|01
CCTCTAACAACTACAAGCTCAGCTTTGGAGCCAGAACCACAGTAACTGTAAGAGCAA
>219|TRAJ21*01 IMGT000013|TRAJ21|J-REGION|TR|TRA|None|01
TACAACTTCAACAAATTTTACTTTGGATCTGGGACCAAACTCAATGTAAAACCAA
>220|TRAJ22*01 IMGT000013|TRAJ22|J-REGION|TR|TRA|None|01
TTTCTTCTGATTCTGGCTGGCAACTGACCTTTGGATCTGGGACACAATTGACCGTTGTACCTG
>221|TRAJ23*01 IMGT000013|TRAJ23|J-REGION|TR|TRA|None|01
TGAATTATAACCAGGCAGGAAAGCTTATCTTCGGACAGGGAACCGAGTTATCTGTGAAGCCCA
>222|TRAJ24*01 IMGT000013|TRAJ24|J-REGION|TR|TRA|None|01
TGACAACTGACAGCTGGGGGAAATTGCAGTTTGGAGCGGGGACCCAGGTTGTGGTCATACCAG
>223|TRAJ25*01 IMGT000013|TRAJ25|J-REGION|TR|TRA|None|01
CAGAAGGACAAAGCTTCTCCTTTATCTTTGGGAAGGGGACAAGGCTGCTTGTCAAGCCAA
>224|TRAJ26*01 IMGT000013|TRAJ26|J-REGION|TR|TRA|None|01
GGGATAACTATGGTCAGAATTTGATCTTCGGTCCCGGAACCAGATTGTCCGTGCTGCCCT
>225|TRAJ27*01 IMGT000013|TRAJ27|J-REGION|TR|TRA|None|01
TAACACCAATGCAGACAAATTAACCTTTGGGGATGGGACTACGCTCATTGTGAAGCCAA
>226|TRAJ28*01 IMGT000013|TRAJ28|J-REGION|TR|TRA|None|01
CATACTCTGGGGCTGGGAGTTACCAACTCACTTTCGGGAAGGGGACCAAACTCTCGGTCATACCAA
>227|TRAJ29*01 IMGT000013|TRAJ29|J-REGION|TR|TRA|None|01
GGAATTCAGGAAACAGAGCTCTTGTCTTTGGAAAGGGCACAAGACTTTCTGTGATTCCAA
>228|TRAJ3*01 IMGT000013|TRAJ3|J-REGION|TR|TRA|None|01
GGGGTACAGCAGTGCTTCCAAGATCATCTTTGGATCAGGGACCAGACTGAGCATCCAGCCAA
>229|TRAJ30*01 IMGT000013|TRAJ30|J-REGION|TR|TRA|None|01
TGAACAGAGATGACAAAATCATCTTTGGAAAAGGGACACGACTTCATATTCTCCCCA
>230|TRAJ31*01 IMGT000013|TRAJ31|J-REGION|TR|TRA|None|01
GAAATAACAATGACAGAGTCATCTTTGGAGATGGAACTCAGCTGGTGGTGAAGCCCA
>231|TRAJ32*01 IMGT000013|TRAJ32|J-REGION|TR|TRA|None|01
TGAATTATGGCGGTAGTGGAAACAAGCTCATCTTTGGAACTGGCACTCTGCTTGCTGTCCAGCCAA
>232|TRAJ33*01 IMGT000013|TRAJ33|J-REGION|TR|TRA|None|01
TGGATAGCAACTATCAGTTAATCTGGGGCGCTGGGACCAAGCTAATTATAAAGCCAG
>233|TRAJ34*01 IMGT000013|TRAJ34|J-REGION|TR|TRA|None|01
TCATAACGCCAACAAGCTCATCTTTGGGACTGGGACCAGATTACAAGTCTTTCCAA
>234|TRAJ35*01 IMGT000013|TRAJ35|J-REGION|TR|TRA|None|01
GATAGGCTTTGGGAATGTGTTGCATTTCGGGTCCGGCACTCAAGTGATTGTTTTACCAC
>235|TRAJ36*01 IMGT000013|TRAJ36|J-REGION|TR|TRA|None|01
TCAAACTGGGGTAAACAACCTCTTCTTTGGGACTGGAACAAGACTCACCGTTCTTCCCT
>236|TRAJ37*01 IMGT000013|TRAJ37|J-REGION|TR|TRA|None|01
TGCCTCTAGCAACACAGGCAAACTGATCTTTGGACAAGGGACAACTTTACAAGTACAACCAG
>237|TRAJ38*01 IMGT000013|TRAJ38|J-REGION|TR|TRA|None|01
TAATGCTGGCAACAACCGTAAGCTGATTTGGGGATTGGGAACAAGCCTGGCAGTAAATCCAA
>238|TRAJ39*01 IMGT000013|TRAJ39|J-REGION|TR|TRA|None|01
TGAATAATAATGCAGGCAACGTGCTCACATTTGGAGGGGGAACAAGGTTAATGGTCAAACCCC

>239|TRAJ4*01 IMGT000013|TRAJ4|J-REGION|TR|TRA|None|01
TGTTTTCTGGTGGCTACGATAAGCTGATTTTTGGAGGAGGGACCAGGCTGGCTGTACACCCAT
>240|TRAJ40*01 IMGT000013|TRAJ40|J-REGION|TR|TRA|None|01
ACTACCACAGGAAACTACAAATACATCTTTGGAACAGGCACCAGGCTGAATGTTTTAGCAA
>241|TRAJ41*01 IMGT000013|TRAJ41|J-REGION|TR|TRA|None|01
GAACTCAAACTCCGGGTATGCACTGAACTTCGGCAAAGGCACCTCGCTGTTGGTCACACCCC
>242|TRAJ42*01 IMGT000013|TRAJ42|J-REGION|TR|TRA|None|01
TGAATTATGGAGGAAGCCGAGGAAATCTCATCTTTGGAAAAGGCACTAAACTCTCTGTTAAACCAA
>243|TRAJ43*01 IMGT000013|TRAJ43|J-REGION|TR|TRA|None|01
ACAACAACAATGACATACGCTTTGGAGCAGGGACCAGACTGACAGTAAAACCAA
>244|TRAJ44*01 IMGT000013|TRAJ44|J-REGION|TR|TRA|None|01
TAAACACCGGCATTGCCAGTAAACTCACCTTTGGGACTGGAACAAGACTTCAGGTCACCCTTG
>245|TRAJ45*01 IMGT000013|TRAJ45|J-REGION|TR|TRA|None|01
CGTATTCAGGAGGAAGTGCTAACAGACTCACCTTTGGCAAAGGGACTCATCTAATCGTCCAGCCCT
>246|TRAJ46*01 IMGT000013|TRAJ46|J-REGION|TR|TRA|None|01
AGAAGAAAAGCAGCGGAGATAAGCTGACTTTTGGGACCGGGACTCGTTTAGCAGTTAGGCCCA
>247|TRAJ47*01 IMGT000013|TRAJ47|J-REGION|TR|TRA|None|01
TGGACTATGGAAACAAGCTGATCTTCGGCACAGGAACCATTCTGAGAGTCAAGCCTT
>248|TRAJ48*01 IMGT000013|TRAJ48|J-REGION|TR|TRA|None|01
TGTCTAACTTTGGAAATGAGAAATTAACCTTTGGGACTGGAACAAGACTCACCATTATACCCA
>249|TRAJ49*01 IMGT000013|TRAJ49|J-REGION|TR|TRA|None|01
GAACACCGGTAACCAGTTGTATTTTGGGAAAGGGACAAGTTTGACAGTCATTCCAA
>250|TRAJ5*01 IMGT000013|TRAJ5|J-REGION|TR|TRA|None|01
TGGACACGGGCAGGAGAGCACTTACTTTTGGGAGTGGAACAAGACTCCAAGTGAAACCAA
>251|TRAJ50*01 IMGT000013|TRAJ50|J-REGION|TR|TRA|None|01
TGACAACCTCCTACAACAAGCTGATGTTTGGGCGGGGGACAAGCTTATCAGTCATTCCAA
>252|TRAJ51*01 IMGT000013|TRAJ51|J-REGION|TR|TRA|None|01
AGATGCGTGACAGCTATGAGAAGCTGATATTTGGAAAGGAGACACGACTAACCGTGAAGTCAA
>253|TRAJ52*01 IMGT000013|TRAJ52|J-REGION|TR|TRA|None|01
CTAATGCTGGTGGTGCTGGCTATGGAAAGCTGACATTTGGACAAGGGACCATCTTGACTGTCTATCCAA
>254|TRAJ52*02 IMGT000026|TRAJ52|J-REGION|TR|TRA|None|02
CTAATGCTGGTGGTGCTGGCTATGGAAAGCTGACATTTGGACAAGGGACCATCTTGACTGTCTACCCAA
>255|TRAJ53*01 IMGT000013|TRAJ53|J-REGION|TR|TRA|None|01
AGAATAGTGGAAGTAGCAACTATAAACTGACATTTGGAAAAGGAACTCTCTTAACTGTGAATCCAA
>256|TRAJ54*01 IMGT000013|TRAJ54|J-REGION|TR|TRA|None|01
GTGTAACTCAGGGAGCCCAGAAGCTGGTATTTGGCCAAGGAACCAGGCTGAGCATCAACCTGA
>257|TRAJ55*01 IMGT000013|TRAJ55|J-REGION|TR|TRA|None|01
ACAAGTGCTGGTAATGCTCCTGTTGGGGGAAGGGAATGAGCACAAAAATAAATCCAA
>258|TRAJ56*01 IMGT000013|TRAJ56|J-REGION|TR|TRA|None|01
TGATACTGGAGCCAATAATAAGCTGACATTTGGAAAAGGAGTAACTCTGAATGTTAGACCAG
>259|TRAJ57*01 IMGT000013|TRAJ57|J-REGION|TR|TRA|None|01
TAACTCAGGGCGGATCTGAAAAGCTCGTCTTTGGAAAGGGAACAAAACTGACAGTAAACCCAT
>260|TRAJ58*01 IMGT000013|TRAJ58|J-REGION|TR|TRA|None|01
TTTAACAAACCGGTGGCTCTAGGTTGACCTTTGGGGAAGGAACACAGCTCACAGTGAATCCTG
>261|TRAJ59*01 IMGT000013|TRAJ59|J-REGION|TR|TRA|None|01
GGAAGGAAGGAAACAGGAAATTTACATTTGGAACGGGACGCGAGTGAGAGTGAAGCTTA
>262|TRAJ6*01 IMGT000013|TRAJ6|J-REGION|TR|TRA|None|01
TGCATCAGGAGGAGGCTACGTACTTACATTTGGAAGAGGAACCAGTCTTATTGTTCATCCGT
>263|TRAJ60*01 IMGT000013|TRAJ60|J-REGION|TR|TRA|None|01
TGAAGAACACCGAGATGCTCAACTTTGGGAAGGGGACTGAGTTAAGTGTGAGCCTGG

>264|TRAJ61*01 IMGT000013|TRAJ61|J-REGION|TR|TRA|None|01
GGTACCGGGTTAATAGGAAACTGACATTTGGAGCCAGCACTAGAGGAATCATGAAACTCA
>265|TRAJ7*01 IMGT000013|TRAJ7|J-REGION|TR|TRA|None|01
ACTATGGGAACAACAGACTCGCTTTTGGGAAGGGGACCCAAGTGGTGGTCATACCAA
>266|TRAJ8*01 IMGT000013|TRAJ8|J-REGION|TR|TRA|None|01
TGAACACAGGCTTTCAGAAACTCGTATTCGGAACTGGCACACAACTTCTGGTCAGTCCAA
>267|TRAJ9*01 IMGT000013|TRAJ9|J-REGION|TR|TRA|None|01
GGAAATACTGGAGGCTTCAAAACTGTCTTTGGAGCAGGAACAAAACTATTTGTTGAAGCAA
>268|TRBJ1-1*01 L43137|TRBJ1-1|J-REGION|TR|TRB|None|01
TGAACACTGAAGCTTTCTTTGGACAAGGCACCAGACTCACAGTTTTAG
>269|TRBJ1-1*02 IMGT000073|TRBJ1-1|J-REGION|TR|TRB|None|02
TGAACACTGAAGCTTTCTTTGGACAAGGCACCAGACTCACAGTTGTAG
>270|TRBJ1-2*01 L43137|TRBJ1-2|J-REGION|TR|TRB|None|01
CTAACTATGACTACACCTTCGGTTCAGGGACCAAGTTAACTGTTGTAG
>271|TRBJ1-3*01 L43137|TRBJ1-3|J-REGION|TR|TRB|None|01
TTCTGGAAACACCGTGTATTTTGGAGAGGGAAGTCGGCTCACTGTTGTAG
>272|TRBJ1-4*01 L43137|TRBJ1-4|J-REGION|TR|TRB|None|01
CAACTAATGAAAAACTGTTTTTTGGCAGTGGAACCCAGCTCTCTGTCTTGG
>273|TRBJ1-5*01 L43137|TRBJ1-5|J-REGION|TR|TRB|None|01
TAGCAATCAGCCCCAGTATTTTGGAGATGGCACTCGACTCTCCGTCCTAG
>274|TRBJ1-6*01 L43137|TRBJ1-6|J-REGION|TR|TRB|None|01
CTCCTATAATTCGCCCCTCTACTTTGGGAACGGGACCAGGCTCACTGTGACGG
>275|TRBJ1-6*02 NW_001114291|TRBJ1-6|J-REGION|TR|TRB|None|02
CTCCTATAATTCGCCCCTCCACTTTGGGAACGGGACCAGGCTCACTGTGACGG
>276|TRBJ1-6*03 IMGT000073|TRBJ1-6|J-REGION|TR|TRB|None|03
CTCCTATAATTCGCCCCTCTACTTTGGGAACGGGACCAGGCTCACTGTGACAG
>277|TRBJ2-1*01 L43138|TRBJ2-1|J-REGION|TR|TRB|None|01
CTCCTACAATGAGCAGTTCTTTGGGCCAGGGACACGGCTCACCGTGCTAG
>278|TRBJ2-1*02 NW_001114291|TRBJ2-1|J-REGION|TR|TRB|None|02
CTCCTACAATGAGCAGTTCTTTGGGCCAGGCACACGGCTCACCGTGCTAG
>279|TRBJ2-2*01 L43138|TRBJ2-2|J-REGION|TR|TRB|None|01
CTAACACCGCGCAGCTGTTCTTTGGAGAAGGCTCTAGGCTGACCGTGCTGG
>280|TRBJ2-2P*01 L43138|TRBJ2-2P|J-REGION|TR|TRB|None|01
CTGAGTGGCGCTGCTGGGCGTCTGGGCCGAGGACTCCTGGTTCTGG
>281|TRBJ2-2P*02 NW_001114291|TRBJ2-2P|J-REGION|TR|TRB|None|02
CTGAGAGGCGCTGCTGGGCGTCTGGGCCGAGGAGTCCTGGTTCTGG
>282|TRBJ2-3*01 L43138|TRBJ2-3|J-REGION|TR|TRB|None|01
AGCACAGATCCGCAGTATTTTGGCCCAGGCACCCGGCTGACAGTGCTCG
>283|TRBJ2-4*01 L43138|TRBJ2-4|J-REGION|TR|TRB|None|01
AGCCAAAACACTCAGTACTTCGGCGCCGGGACCCGGCTCTCAGTGCTGG
>284|TRBJ2-5*01 L43138|TRBJ2-5|J-REGION|TR|TRB|None|01
ACCAAGAGACCCAGTACTTCGGACCAGGCACGCGGCTCCTGGTGCTCG
>285|TRBJ2-6*01 L43138|TRBJ2-6|J-REGION|TR|TRB|None|01
CTCTGGGGCCAGCGTCCTGACTTTCGGGGCCGGCAGCCGGCTGACCGTGCTGG
>286|TRBJ2-7*01 L43138|TRBJ2-7|J-REGION|TR|TRB|None|01
CTCCTACGAGCAGTACTTCGGGCCGGGCACCAGGCTCACAGTCATAG
>287|TRDJ1*01 IMGT000013|TRDJ1|J-REGION|TR|TRD|None|01
ACACTGATAAACTCATCTTTGGAAAAGGAACCCGTGTGACTGTGGAACCAA
>288|TRDJ2*01 IMGT000013|TRDJ2|J-REGION|TR|TRD|None|01
CTTTCACAGCACAACTCTTCTTTGGAAAGGGAACTCAACTCATCGTGGAACCAG

>289|TRDJ3*01 IMGT000013|TRDJ3|J-REGION|TR|TRD|None|01
CTCCTGGGACACCCGACAGATGTTTTTGGAACTGGCACCAAACTCTTCGTGGAGCCCC
>290|TRDJ4*01 IMGT000013|TRDJ4|J-REGION|TR|TRD|None|01
CCAGACCACTGATCTTTGGCAAAGGAACCTATCTGGAGGTAGAAAGAC
>291|TRGJ1-1*01 IMGT000059|TRGJ1-1|J-REGION|TR|TRG|None|01
ATAGCACTGGTTGGATCAAGATATTTGCTGAAGGTACTAAGCTCATAGTAACTTCGCCTG
>292|TRGJ1-2*01 IMGT000059|TRGJ1-2|J-REGION|TR|TRG|None|01
TGGGCAACAGTTTGGCAGAAAAGTCAAGCTATTTGGTCCCGGAACAAAGCTCATCATTACAG
>293|TRGJ2-1*01 IMGT000059|TRGJ2-1|J-REGION|TR|TRG|None|01
ATAGCACTTGGTGGATCAAGAAATTTGCTGAAGGGACTAGGCTCATAGTAACTTCGCCTG
>294|TRGJ2-2*01 IMGT000059|TRGJ2-2|J-REGION|TR|TRG|None|01
GTAGGCAACAGTTTGGCAAAAAAATCAAGGTGTTTGGCCCCTGAACAAAGCTCATCATTACAG
>295|TRGJ2-3*01 IMGT000059|TRGJ2-3|J-REGION|TR|TRG|None|01
GAATTATTATAAGAAACTCTTTGGCAGTGGAACAACACTTGTTGTCACAG
>296|TRAC*01 IMGT|TRAC|C-REGION|TR|TRA|None|01
ATATCCAGAACCCTGACCCTGCCGTGTACCAGCTGAGAGGCTCTAAATCCAATGACACCTCTGTCTGCCT
ATTTACTGATTTTGATTCTGTAATGAATGTGTCACAAAGCAAGGATTCTGACGTGCATATCACAGACAAA
ACTGTGCTAGACATGAGGTCTATGGACTTTAAGAGCAACGGTGCTGTGGCCTGGAGCAACAAATCCGATT
TTGCATGTACAAGCGCCTTCAAGGACAGCGTTATTCCAGCAGACACCTTCTTCCCCGGCACAGAAAGTGT
CTGTGATGCCAACCTGGTTGAGAAAAGCTTTGAAACAGATATGAACCTAAACTTTCAAAACCTGTCAGTG
ATTGGGTTCCGAATCCTCCTCCTGAAAGTGGCCGGGTTTAATCTGCTCATGACGCTGCGGCTGTGGTCCA
GCTGA
>297|TRAC*02 TRAC_205_Ensembl_CDS|TRAC|C-REGION|TR|TRA|None|02
ATGAATGTGTCACAAAGCAAGGATTCTGACGTGCATATCACAGACAAAACTGTGCTAGACATGAGGTCTA
TGGACTTTAAGAGCAACGGTGCTGTGGCCTGGAGCAACAAATCCAATTTTGCATGTACAAGCGCCTTCAA
GGACAGCGTTATTCCAGCAGACACCTTCTTCCCCGGCACAGAAAGTGTCTGTGATGCCAACCTGGTTGAG
AAAAGCTTTGAAACAGATATGAACCTAAACTTTCAAAACCTGTCAGTGATTGGGTTCCGAATCCTCCTCC
TGAAAGTGGCCGGGTTTAATCTGCTCATGACGCTGCGGCTGTGGTCCAGCTGA
>298|TRAC*03 TRAC_201_Ensembl_CDS|TRAC|C-REGION|TR|TRA|None|03
ATGAATGTGTCACAAAGCAAGGATTCTGACGTGCATATCACAGACAAAACTGTGCTAGACATGAGGTCTA
TGGACTTTAAGAGCAACGGTGCTGTGGCCTGGAGCAACAAATCCAATTTTGCATGTACAAGCGCCTTCAA
GGACAGCGTTATTCCAGCAGACACCTTCTTCCCCGGCACAGAAAGTGTCTGTGATGCCAACCTGGTTGAG
AAAAGCTTTGAAACAGATATGAACCTAAACTTTCAAAACCTGTCAGTGATTGGGTTCCGAATCCTCCTCC
TGAAAGTGGCCGGGTTTAATCTGCTCATGACGCTGCGGCTGTGGTCCAGCTGA
>299|TRAC*04 TRAC_202_Ensembl_CDS|TRAC|C-REGION|TR|TRA|None|04
ATGAATGTGTCACAAAGCAAGGATTCTGACGTGCATATCACAGACAAAACTGTGCTAGACATGAGGTCTA
TGGACTTTAAGAGCAACGGTGCTGTGGCCTGGAGCAACAAATCCAATTTTGCATGTACAAGCGCCTTCAA
GGACAGCGTTATTCCAGCAGACACCTTCTTCCCCGGCACAGAAAGTGTCTGTGATGCCAACCTGGTTGAG
AAAAGCTTTGAAACAGATATGAACCTAAACTTTCAAAACCTGTCAGTGATTGGGTTCCGAATCCTCCTCC
TGAAAGTGGCCGGGTTTAATCTGCTCATGACGCTGCGGCTGTGGTCCAGCTGA
>300|TRAC*05 TRAC_203_Ensembl_CDS|TRAC|C-REGION|TR|TRA|None|05
ACTGGGGTAAACAACCTCTTCTTTGGGACTGGAACAAGACTCACCGTTCTTCCAGATATCCAGAACCCTG
ACCCTGCCGTGTACCAGCTGAGAGGCTCTAAATCCAATGACACCTCTGTCTGCCTATTTACTGATTTTGA
TTCTGTAATGAATGTGTCACAAAGCAAGGATTCTGACGTGCATATCACAGACAAAACTGTGCTAGACATG
AGGTCTATGGACTTTAAGAGCAACGGTGCTGTGGCCTGGAGCAACAAATCCAATTTTGCATGTACAAGCG
CCTTCAAGGACAGCGTTATTCCAGCAGACACCTTCTTCCCCGGCACAGAAAGTGTCTGTGATGCCAACCT
GGTTGAGAAAAGCTTTGAAACAGATATGAACCTAAACTTTCAAAACCTGTCAGTGATTGGGTTCCGAATC
CTCCTCCTGAAAGTGGCCGGGTTTAATCTGCTCATGACGCTGCGGCTGTGGTCCAGCTGA
>301|TRAC*06 TRAC_204_Ensembl_CDS|TRAC|C-REGION|TR|TRA|None|06
ATGAATGTGTCACAAAGCAAGGATTCTGACGTGCATATCACAGACAAAACTGTGCTAGACATGAGGTCTA

TGGACTTTAAGAGCAACGGTGCTGTGGCCTGGAGCAACAAATCCAATTTTGCATGTACAAGCGCCTTCAA
GGACAGCGTTATTCCAGCAGACACCTTCTTCCCCGGCACAGAAAGTGTCTGTGATGCCAACCTGGTTGAG
AAAAGCTTTGAAACAGATATGAACCTAAACTTTCAAAACCTGTCAGTGATTGGGTTCCGAATCCTCCTCC
TGAAAGTGGCCGGGTTTAATCTGCTCATGACGCTGCGGCTGTGGTCCAGCTGA
>302|TRBC1*01 NW_001114291|TRBC1|C-REGION|TR|TRB|None|01
AGGACCTGAAAAAGGTGTTCCCACCCAAGGTCGCTGTGTTTGAGCCATCAGAAGCAGAGATCTCCCACAC
CCAAAAGGCCACGCTGGTGTGCCTGGCCACAGGCTTCTACCCCGACCACGTGGAGCTGAGCTGGTGGGTG
AACGGGAAAGAGGTGCACAGTGGGGTCAGCACGGACCCACAGCCCCTCAAGGAGCAGCCCGCCCTCGAGG
ACTCCAGATACTGCCTGAGCAGCCGCCTGAGGGTCTCGGCCACCTTCTGGCACAACCCCCGCAACCACTT
CCGCTGCCAAGTCCAGTTCTATGGGCTCTCGGAGGATGACGAGTGGACCGAGGACAGGGACAAGCCCATC
ACCCAAAAGATCAGCGCCGAGGTCTGGGGTAGAGCAGACTGTGGCTTCACCTCGGTGTCCTACCAGCAAG
GGGTCCTGTCTGCCACCATCCTCTATGAGATCCTGCTGGGGAAGGCCACCCTGTATGCTGTGCTGGTCAG
TGCCCTCATGTTGATGGCCATGGTCAAGAGGAAGGATTTC
>303|TRBC1*02 IMGT000073|TRBC1|C-REGION|TR|TRB|None|02
AGGACCTGAAAAAGGTGTTCCCACCCAAGGTCGCTGTGTTTGAGCCATCAGAAGCAGAGATCTCCCACAC
CCAAAAGGCCACGCTGGTGTGCCTGGCCACAGGCTTCTACCCCGACCACGTGGAGCTGAGCTGGTGGGTG
AACGGGAAAGAGGTGCACAGTGGGGTCAGCACGGACCCACAGCCCCTCAAGGAGCAGCCCGCCCTCGAGG
ACTCCAGATACTGCCTGAGCAGCCGCCTGAGGGTCTCAGCCACCTTCTGGCACAACCCCCGCAACCACTT
CCGCTGCCAAGTCCAGTTCTATGGGCTCTCGGAGGATGACGAGTGGACCGAGGACAGGGACAAGCCCATC
ACCCAAAAGATCAGCGCCGAGGTCTGGGGTAGAGCAGACTGTGGCTTCACCTCAGTGTCCTACCAGCAAG
GGGTCCTGTCTGCCACCATCCTCTATGAGATCCTGCTGGGGAAGGCCTCCCTGTATGCTGTGCTGGTCAG
TGCCCTCATGTTGATGGCCATGGTCAAGAGGAAGGATTTC
>304|TRBC2*03 IMGT000073|TRBC2|C-REGION|TR|TRB|None|01
AGGACCTGAAAAAAGTGTTCCCACCCAAGGTCGCTGTGTTTGAGCCATCAGAAGCAGAGATCTCCCACAC
CCAAAAGGCCACGCTGGTGTGCCTGGCCACAGGCTTCTACCCCGACCACGTGGAGTTGAGCTGGTGGGTG
AACGGGAAAGAGGTGCACAGTGGGGTCAGCACGGACCCACAGCCCCTCAAGGAGCAGCCCACCCTCGAGG
ACTCCAGATACTGCCTGAGCAGCCGCCTGAGGGTCTCGGCCACCTTCTGGCACAACCCCCGCAACCACTT
CCGCTGCCAAGTCCAGTTCTATGGGCTCTCGGAGGATGACGAGTGGACCGAGGACAGGGACAAGCCCATC
ACCCAAAAGATCAGCGCTGAGGCCTGGGGTAGAGCAGACTGTGGCTTCACCTCTGAGTCTTACCAGCAAG
GGGTCCTGTCTGCCACCATCCTCTATGAGATCTTGCTAGGGAAGGCCACCTTGTATGCCGTGCTGGTCAG
TGCCCTCGTGCTGATGGCCATGGTCAAGAGAAAGGATTCC

Supplemantary Table 5: VDJ Segment Usage

Count of Regions in Our Reference, and Share Found in our 30 V1 Primer VDJ Samples

Chain	Type	Count in Reference	Count in Data	Percent of Reference Regions Found
TRA	C-REGION	1	1	100.0%
TRA	J-REGION	61	56	91.8%
TRA	L-REGION+V-REGION	54	47	87.0%
TRB	C-REGION	2	2	100.0%
TRB	D-REGION	2	2	100.0%
TRB	J-REGION	14	13	92.9%
TRB	L-REGION+V-REGION	62	58	93.5%

Supplementary Figure **1**
A − Distribution of TCR Chain Length

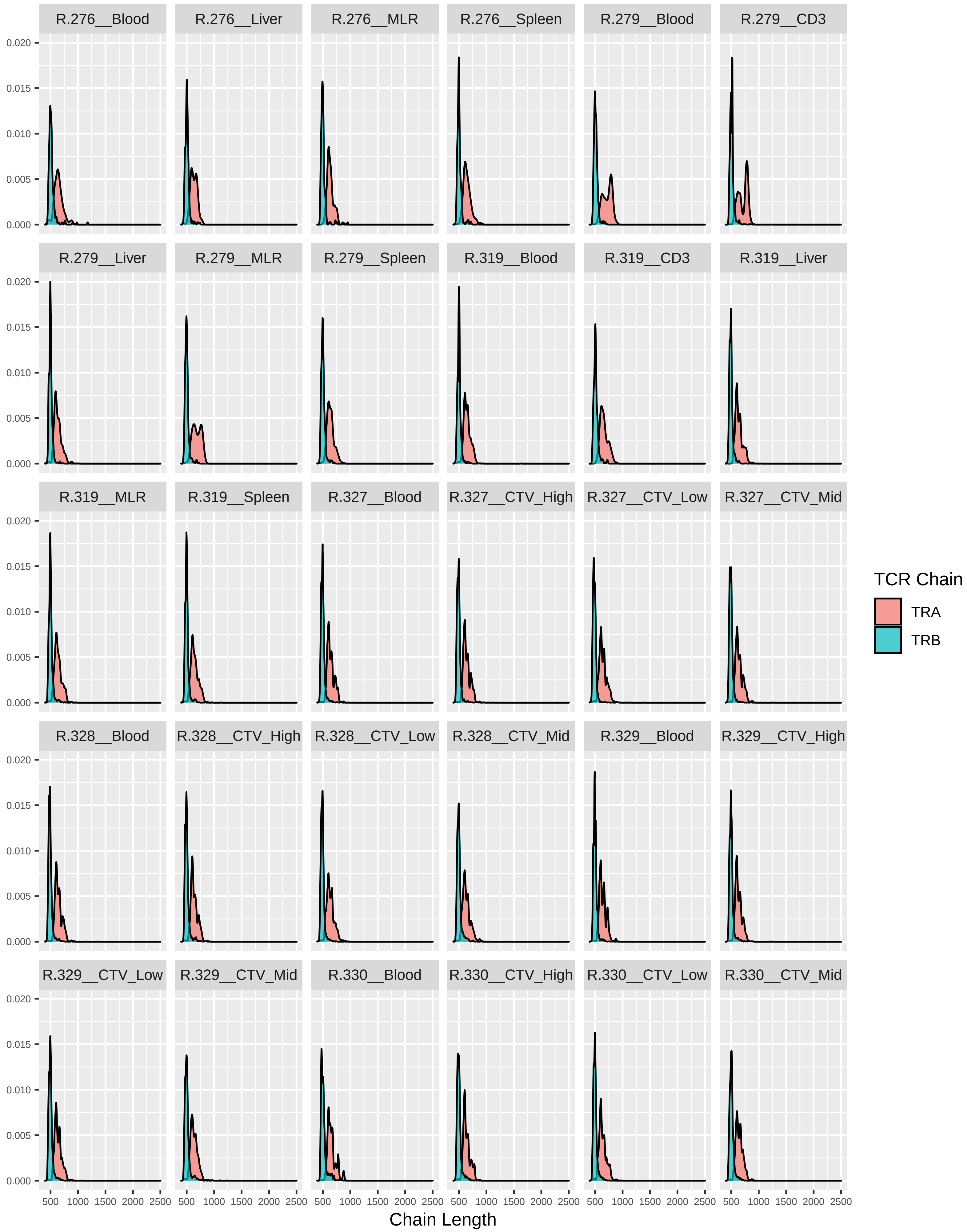

B − Distribution of TCR Chain Length (x−axis limited)

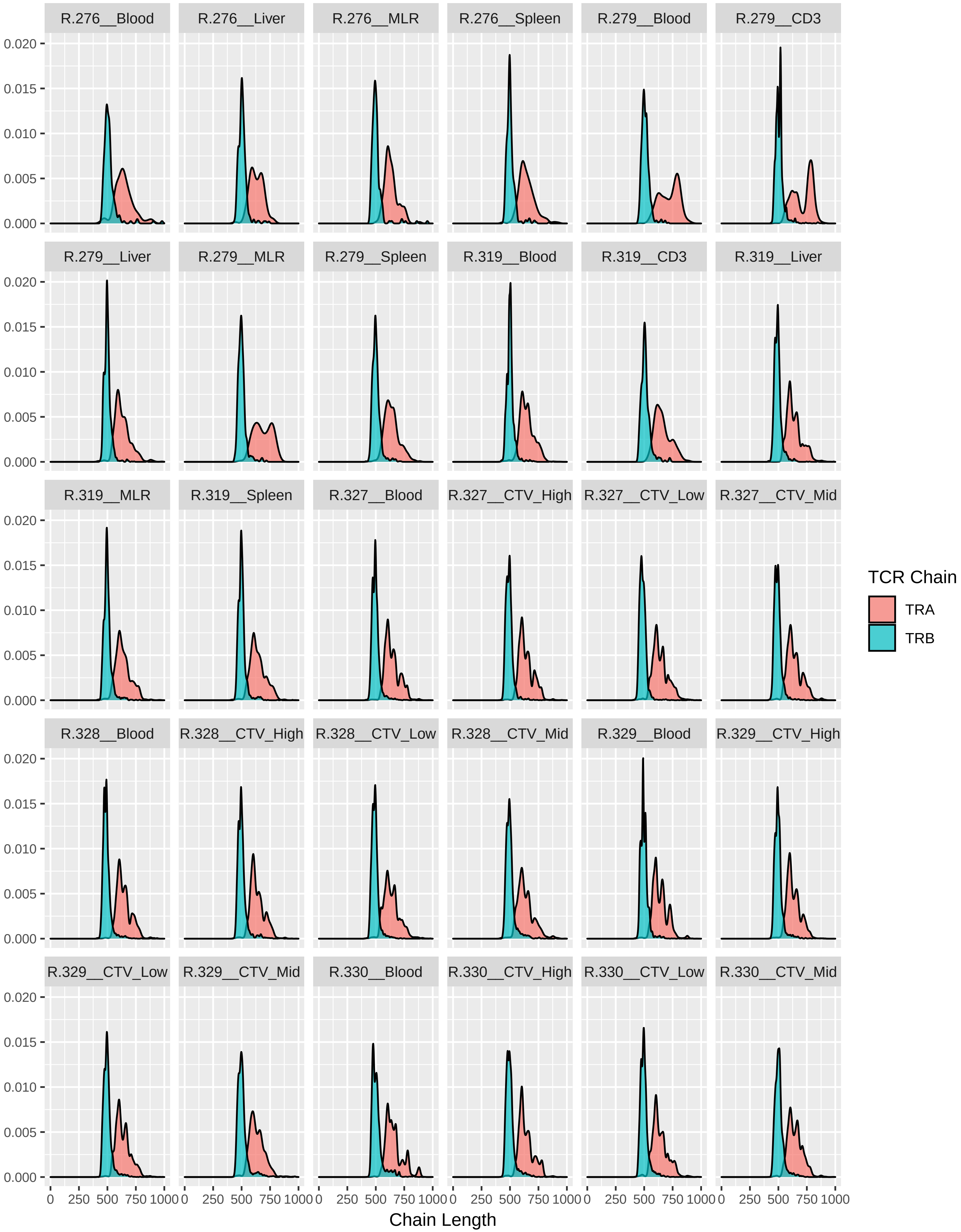

89 outlier chains with length > 1000bp removed

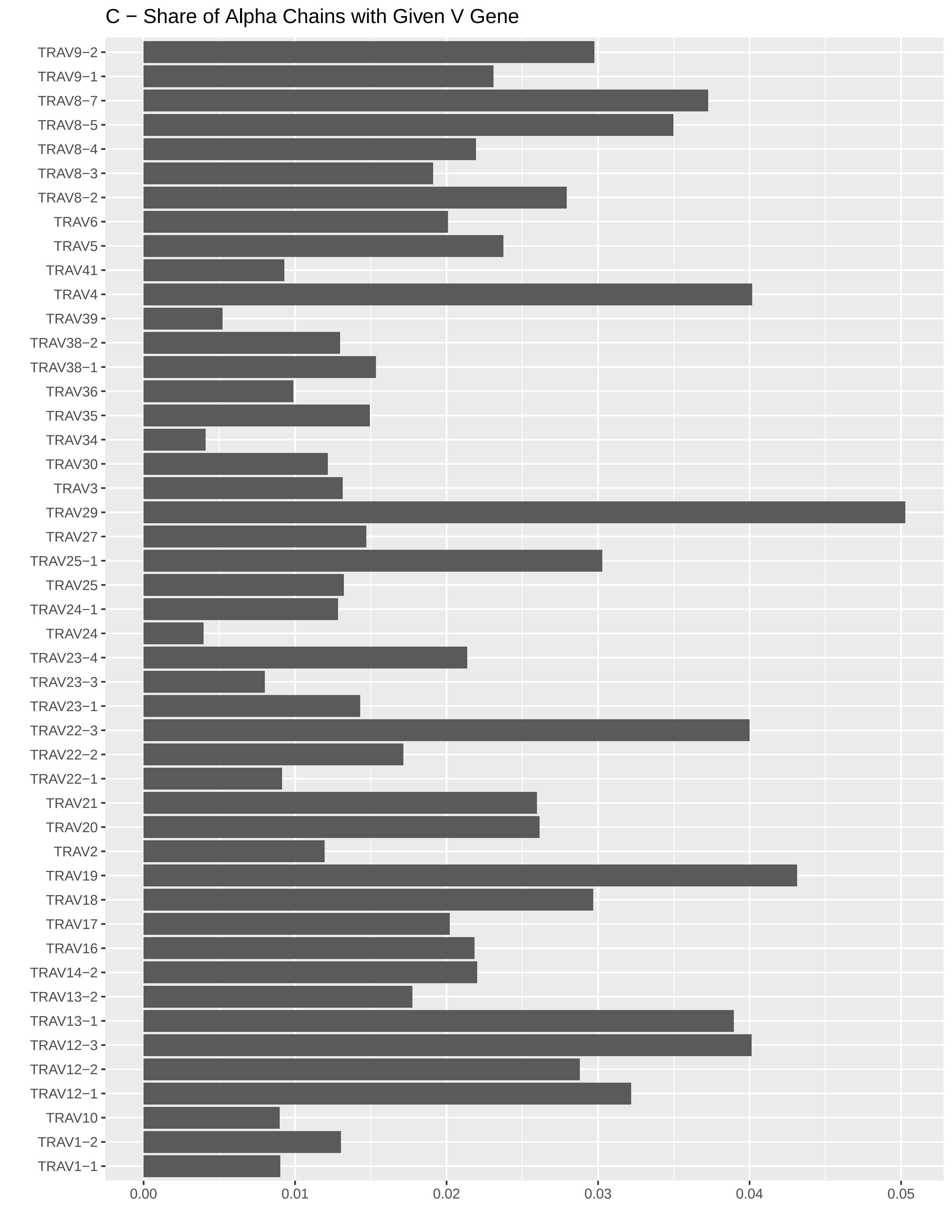
C − Share of Alpha Chains with Given V Gene
TRAV9−2
TRAV9−1
TRAV8−7
TRAV8−5
TRAV8−4
TRAV8−3
TRAV8−2
TRAV6
TRAV5
TRAV41
TRAV4
TRAV39
TRAV38−2
TRAV38−1
TRAV36
TRAV35
TRAV34
TRAV30
TRAV3
TRAV29
TRAV27
TRAV25−1
TRAV25
TRAV24−1
TRAV24
TRAV23−4
TRAV23−3
TRAV23−1
TRAV22−3
TRAV22−2
TRAV22−1
TRAV21
TRAV20
TRAV2
TRAV19
TRAV18
TRAV17
TRAV16
TRAV14−2
TRAV13−2
TRAV13−1
TRAV12−3
TRAV12−2
TRAV12−1
TRAV10
TRAV1−2
TRAV1−1
0.00
0.01
0.02
0.03
0.04
0.05

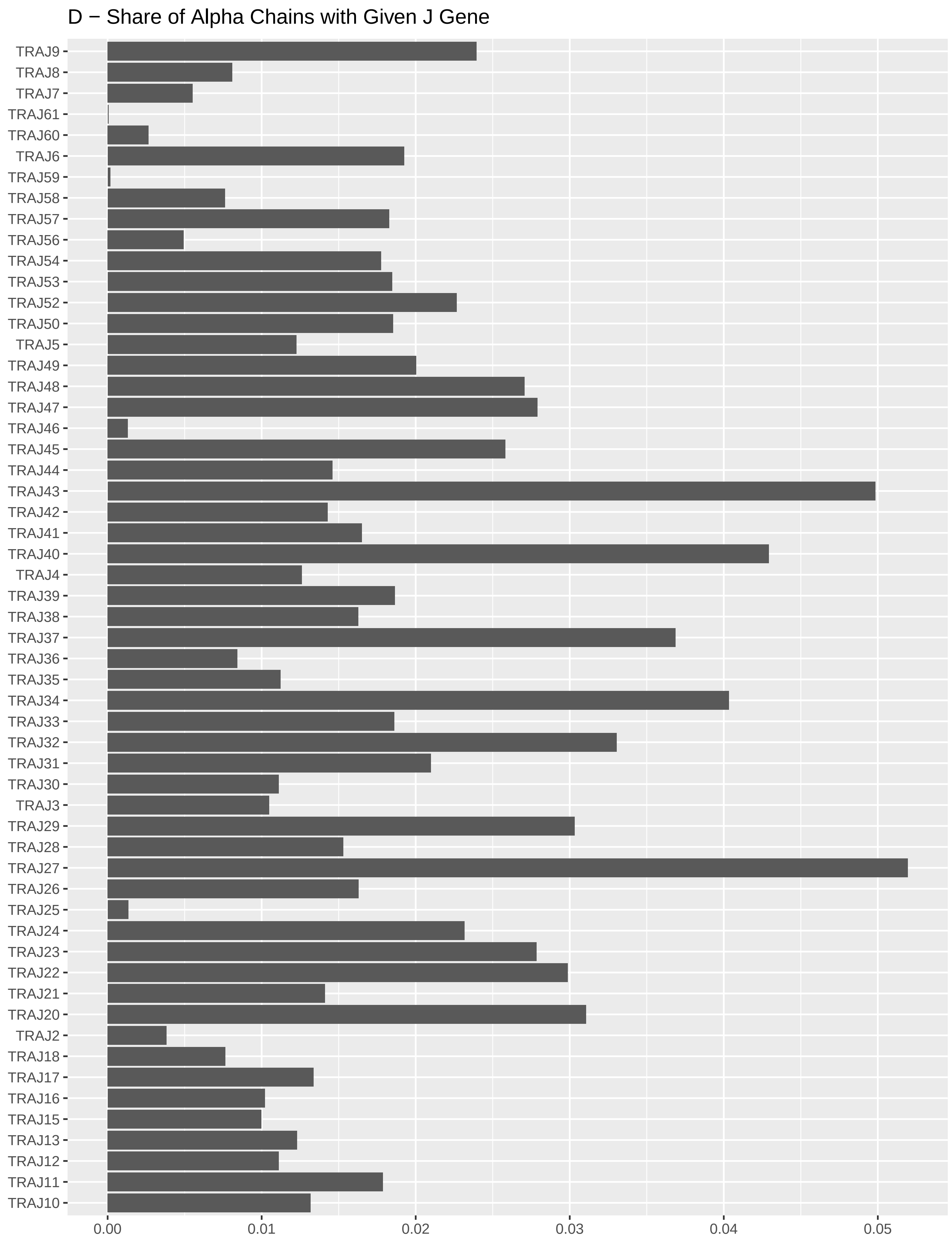

D − Share of Alpha Chains with Given J Gene
TRAJ9
TRAJ8
TRAJ7
TRAJ61
TRAJ60
TRAJ6
TRAJ59
TRAJ58
TRAJ57
TRAJ56
TRAJ54
TRAJ53
TRAJ52
TRAJ50
TRAJ5
TRAJ49
TRAJ48
TRAJ47
TRAJ46
TRAJ45
TRAJ44
TRAJ43
TRAJ42
TRAJ41
TRAJ40
TRAJ4
TRAJ39
TRAJ38
TRAJ37
TRAJ36
TRAJ35
TRAJ34
TRAJ33
TRAJ32
TRAJ31
TRAJ30
TRAJ3
TRAJ29
TRAJ28
TRAJ27
TRAJ26
TRAJ25
TRAJ24
TRAJ23
TRAJ22
TRAJ21
TRAJ20
TRAJ2
TRAJ18
TRAJ17
TRAJ16
TRAJ15
TRAJ13
TRAJ12
TRAJ11
TRAJ10
0.00
0.01
0.02
0.03
0.04
0.05

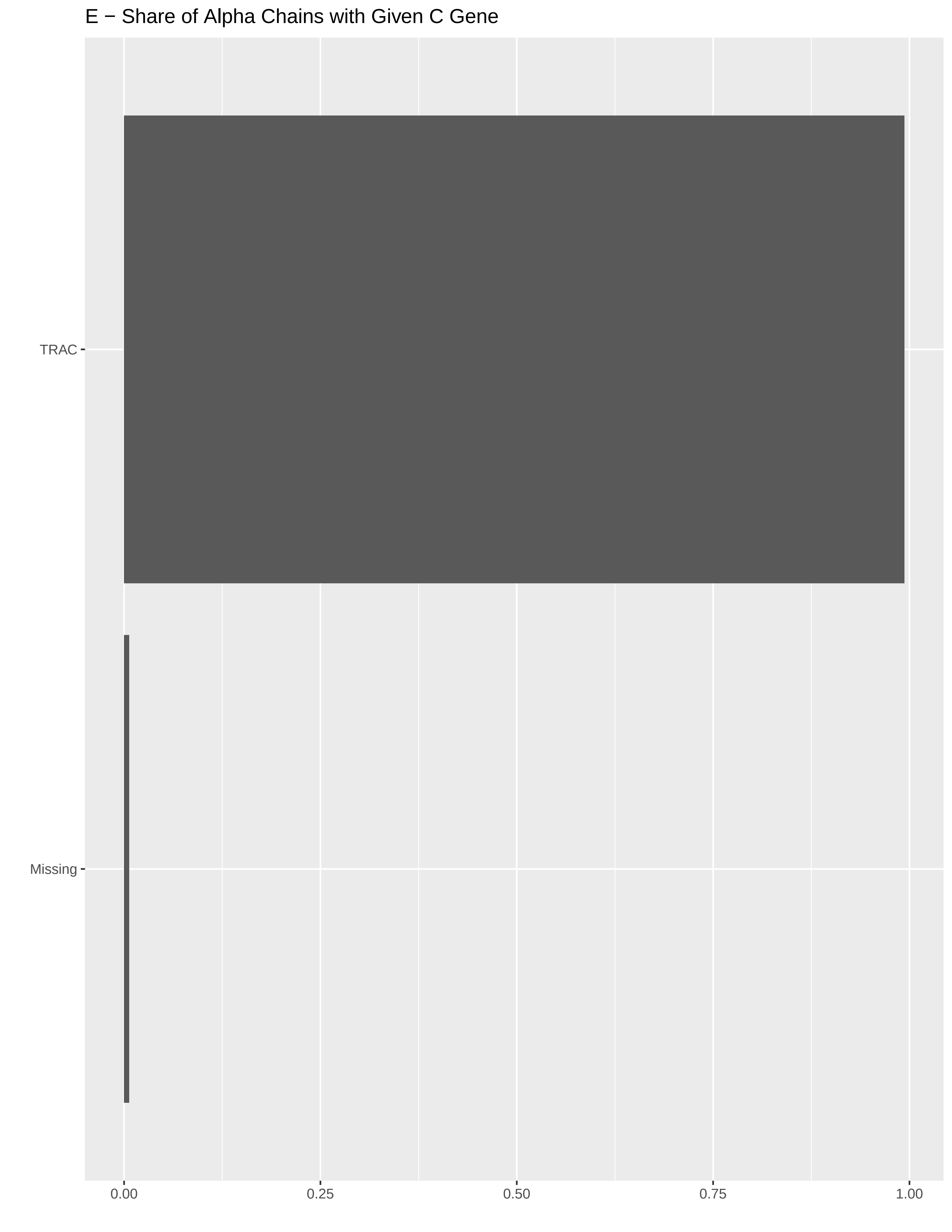
E − Share of Alpha Chains with Given C Gene
TRAC
Missing
0.00
0.25
0.50
0.75
1.00

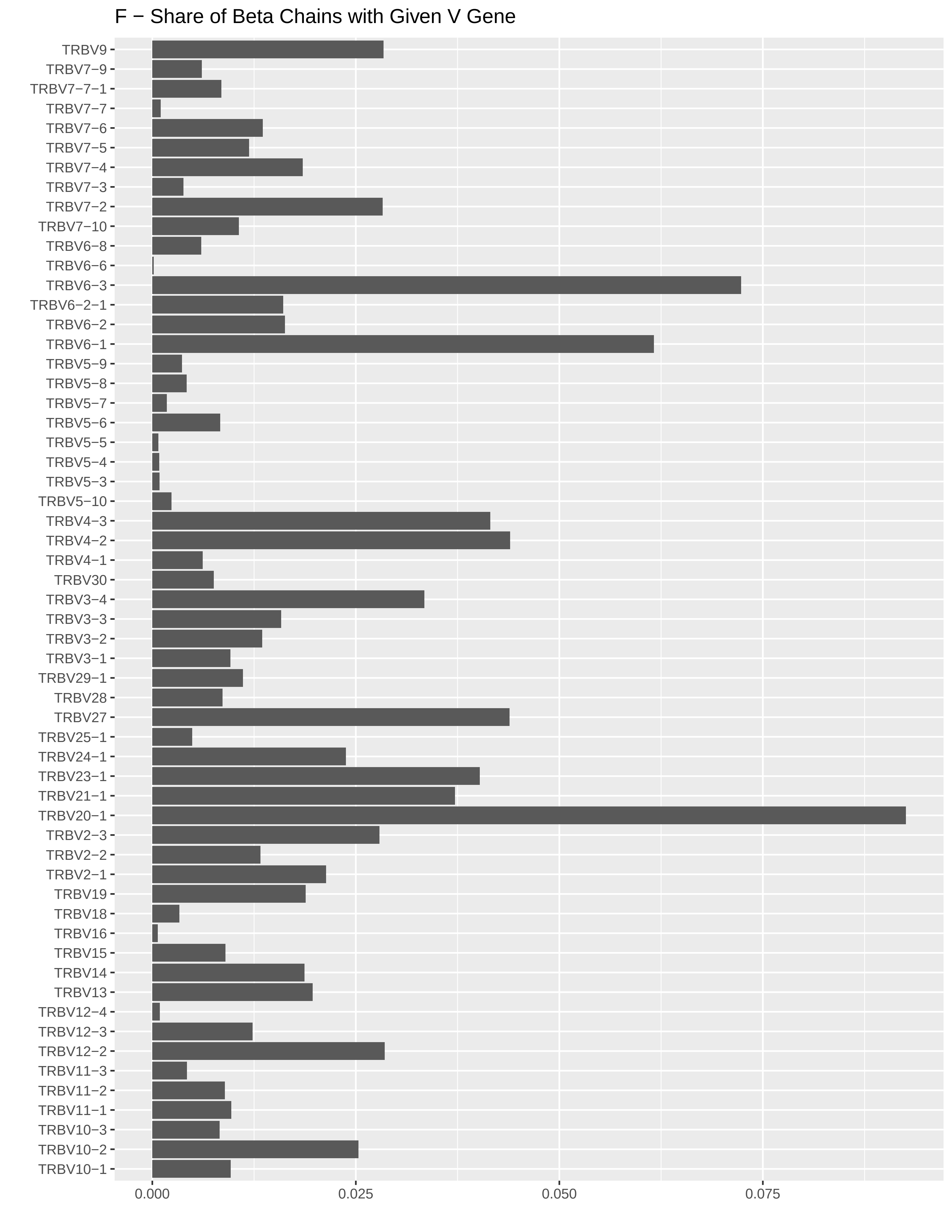
F − Share of Beta Chains with Given V Gene
TRBV9
TRBV7−9
TRBV7−7−1
TRBV7−7
TRBV7−6
TRBV7−5
TRBV7−4
TRBV7−3
TRBV7−2
TRBV7−10
TRBV6−8
TRBV6−6
TRBV6−3
TRBV6−2−1
TRBV6−2
TRBV6−1
TRBV5−9
TRBV5−8
TRBV5−7
TRBV5−6
TRBV5−5
TRBV5−4
TRBV5−3
TRBV5−10
TRBV4−3
TRBV4−2
TRBV4−1
TRBV30
TRBV3−4
TRBV3−3
TRBV3−2
TRBV3−1
TRBV29−1
TRBV28
TRBV27
TRBV25−1
TRBV24−1
TRBV23−1
TRBV21−1
TRBV20−1
TRBV2−3
TRBV2−2
TRBV2−1
TRBV19
TRBV18
TRBV16
TRBV15
TRBV14
TRBV13
TRBV12−4
TRBV12−3
TRBV12−2
TRBV11−3
TRBV11−2
TRBV11−1
TRBV10−3
TRBV10−2
TRBV10−1
0.000
0.025
0.050
0.075

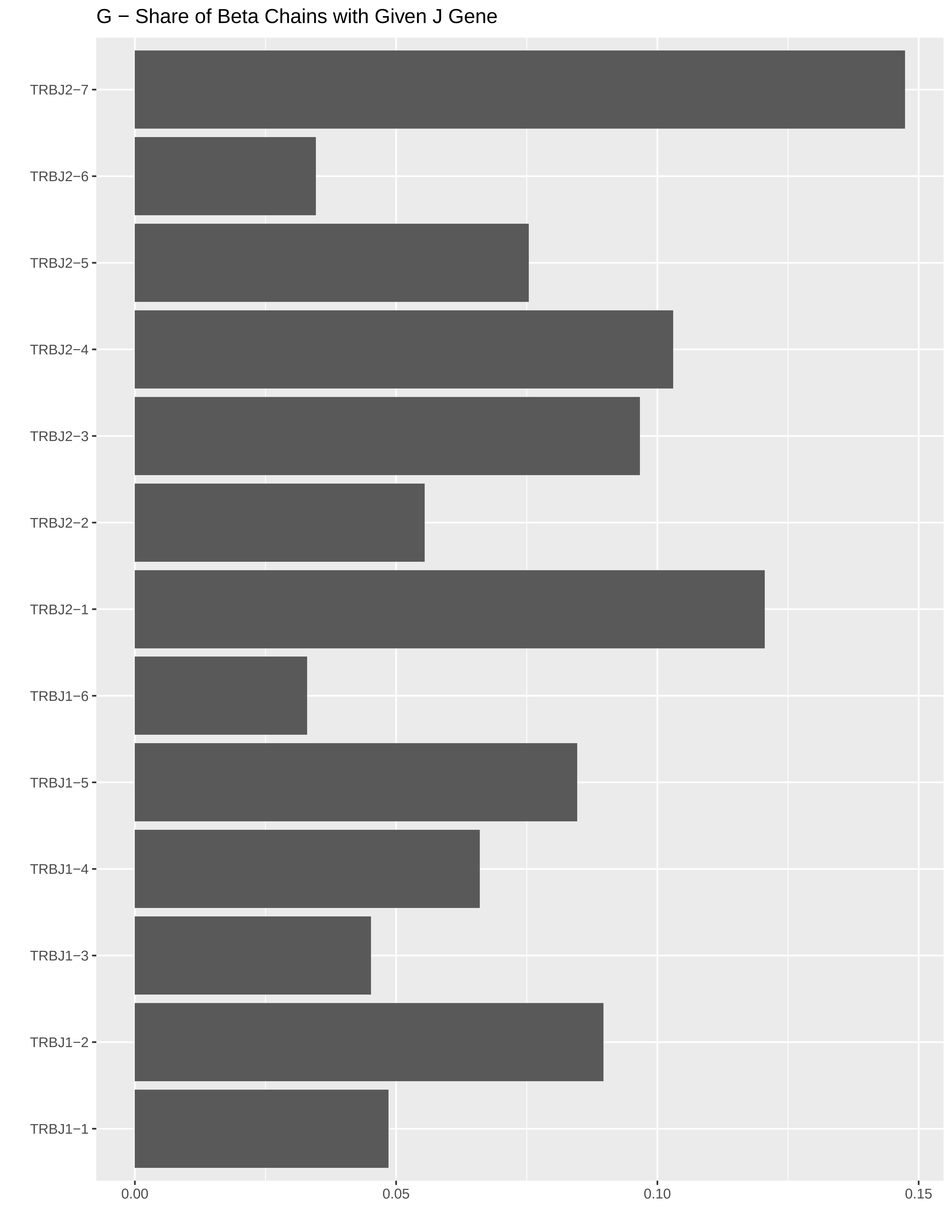
G − Share of Beta Chains with Given J Gene
TRBJ2−7
TRBJ2−6
TRBJ2−5
TRBJ2−4
TRBJ2−3
TRBJ2−2
TRBJ2−1
TRBJ1−6
TRBJ1−5
TRBJ1−4
TRBJ1−3
TRBJ1−2
TRBJ1−1
0.00
0.05
0.10
0.15

H − Share of Beta Chains with Given D Gene

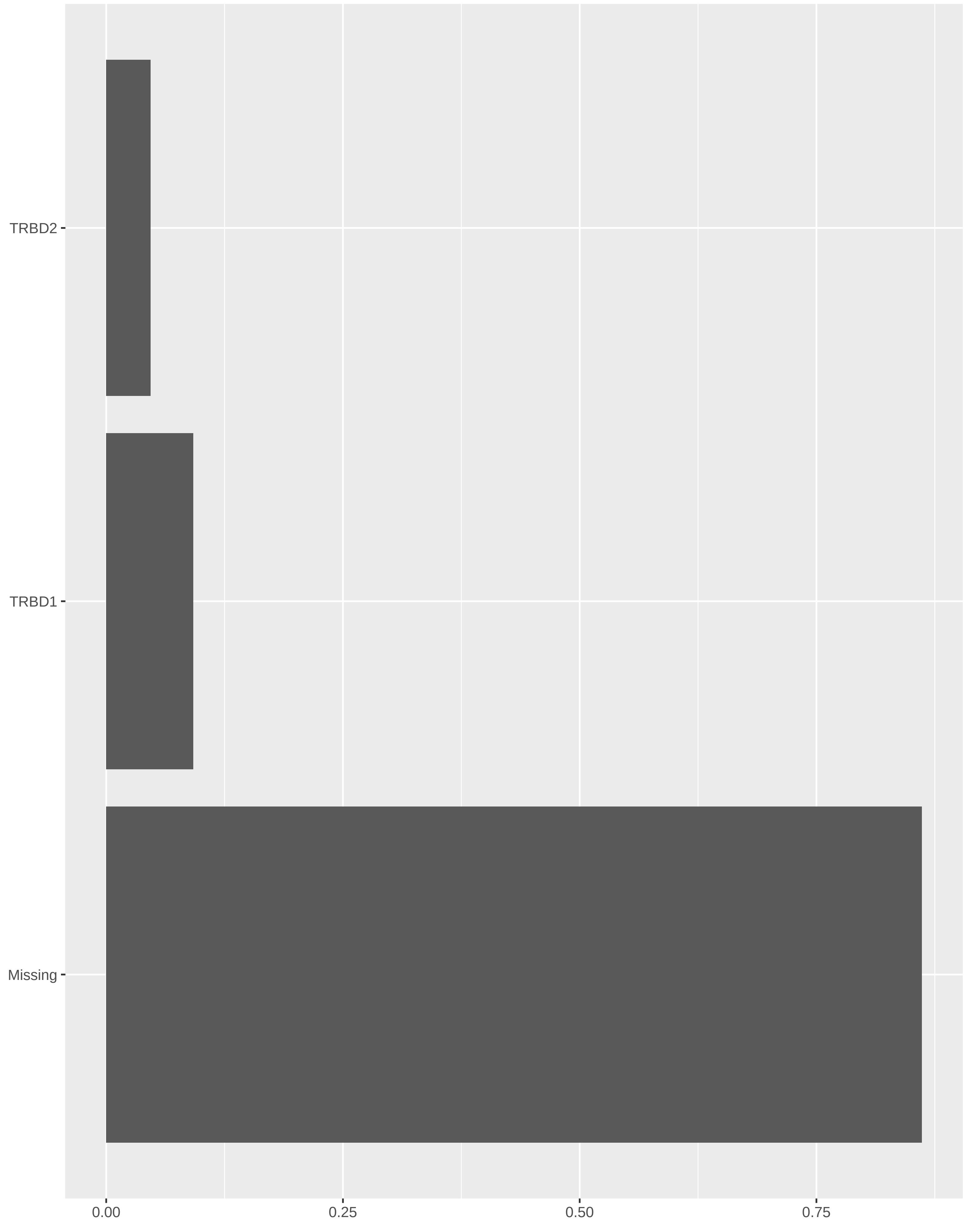

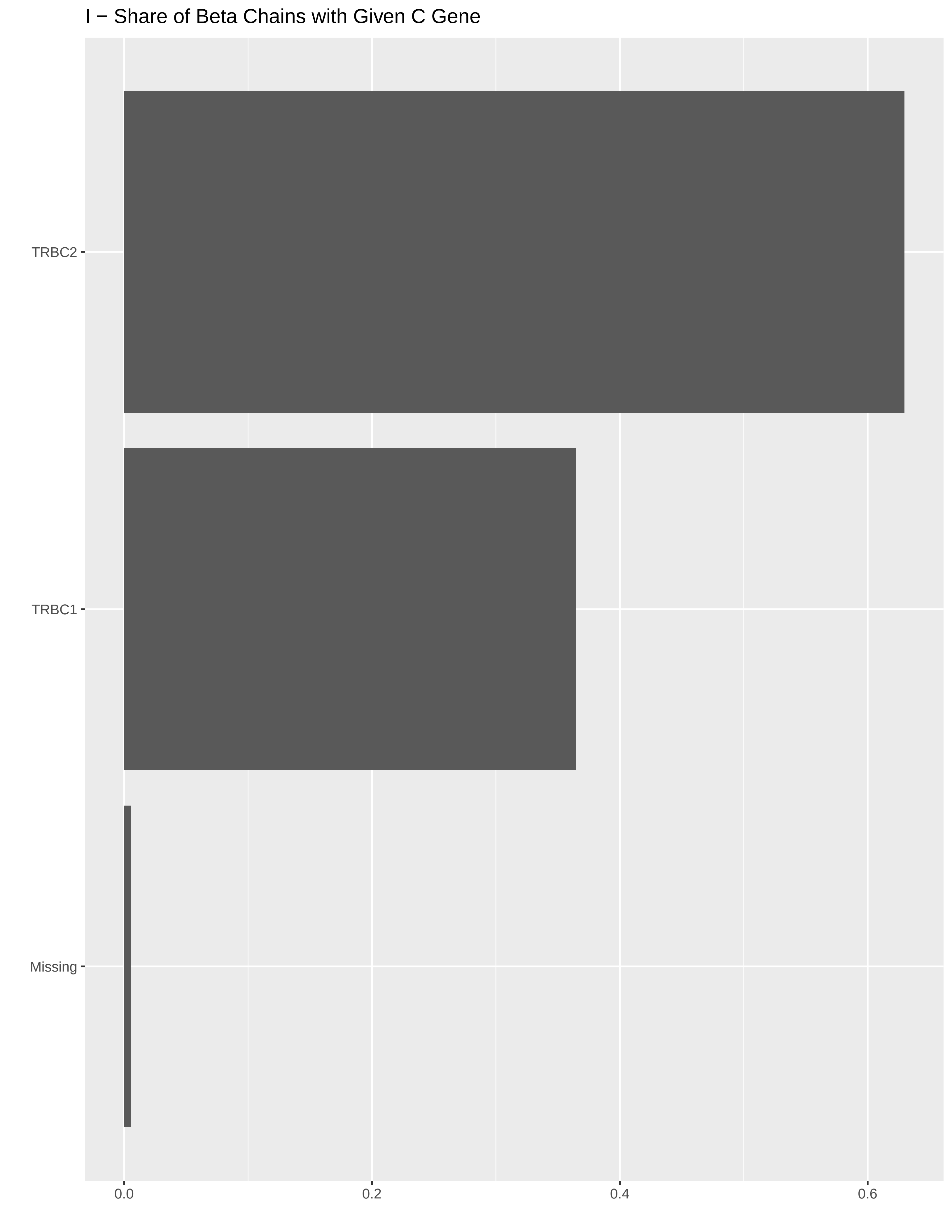

I − Share of Beta Chains with Given C Gene
TRBC2
TRBC1
Missing
0.0
0.2
0.4
0.6

Figure S1. **Summary Information for Alpha and Beta Chains Assembled from VDJ Samples.**

(A) Distribution of alpha chain and beta chain length among assembled chains in each VDJ sample. **(B)** Distribution of alpha chain and beta chain length among assembled chains in each VDJ sample, with 89 outliers (chain length > 1000bp) removed. **(C)** Distribution of alpha chain CDR3 length (amino acids), with three outliers (CDR3 > 30aa) removed. **(D)** Distribution of beta chain CDR3 length (amino acids). **(E)** Share of alpha chains with given V gene. **(F)** Share of alpha chains with given J gene. **(G)** - Share of alpha chains with given C gene. **(H)** − Share of beta chains with given V gene. **(I)** - Share of beta chains with given J gene. **(J)** - Share of beta chains with given D gene. **(K)** - Share of beta chains with given C gene.
